# Supplementary material for: A review and in silico screening of plant-derived snake venom/toxin inhibitors: ADMET, drug-likeness, and medicinal chemistry profiling
Source: PLoS Negl Trop Dis. 2025 Oct 9;19(10):e0013579. doi: 10.1371/journal.pntd.0013579 (PMC12527186; doi:10.1371/journal.pntd.0013579)
Supplement: S1 Fig — (DOCX) [file pntd.0013579.s001.docx]

**Figure S1: Chemical structures of Plants Derived Compounds with Antivenom Properties**

**Alkaloids**

**1**

**2** △^2^ dehydro, △^9^ dehydro, 22α, 23α

**3** △^2^ dehydro, △^9^ dehydro, 22α, 23α

**4** △^2^ dehydro, △^9^ dehydro, 22 β, 23 β

**5** △^2^ dehydro, △^9^ dehydro, 22 α, 23 β

**6** △^2^ dehydro, △^9^ dehydro, 22 β, 23 β

**7** E **8** Z  **9**

**10**  **11**   **12** R =OH

**13** R =Cl

**14**

**15** R_1_ = OH, R_2_ = R_4_ = H, R_3_ = OCH_3_

**16** R_1_ = R_3_ = R_4_ = H, R_2_ = OCH_3_

**17** R_1_ = R_4_ = H, R_2_ = R_3_ = OH

**Benzenoids**

**18** R_1_ = OH, R_2_ = OCH_3_, R_3_= R_4_ = H

**19** R_1_ = OH, R_2_ = OCH_3_, R_3_= R_4_ = H

**20** R_1_ = H, R_2_ = H, R_3_= OCH_3_, R_4_ = H

**21** R_1_ = OH, R_2_ =H, R_3_= H, R_4_ = H

**22** R_1_ = H, R_2_ = OH, R_3_= OH, R_4_ = OH

**23** R_1_ = H, R_2_ = OCH_3_, R_3_= OH, R_4_ = H

**24**  **25**

**26**

**Hydroxycinnamic Acids**

**27**

**28** R = OH

**29** R = H

**30** R = OCH_3_

**31**

**32**  **33**  **34** R = OH **35** R = OCH_3_

**Tannins**

**36**

**37**  **38**  **39**

**Coumarins**

**42**

**40** R_1_ = R_2_ = R_3_= H

**41** R_1_ =H, R_2_ = OCH_3_, R_3_= OH

**Flavonoids**

**43** R_1_ = R_3_ = H, R_2_= OH

**44** R_1_ =R_2_ = OH, R_3_= H

**45** R_1_ = OCH_3_

**46** R_1_ = OH

**47**

**48** R_1_ = R_2_ = OH, R_3_= H

**49** R_1_ = R_3_ = H, R_2_= OH

**50** R_1_ =H, R_2_ = R_3_= OH

**51** R_1_ = R_2_ = R_3_= OH

**52** **53** **54**

**55** **56** R_1_ =H; **57** R_1_=OH

**58**  **59**

**60**  **61**

**62** **63**  **64**

**65** **66** **67**  **68**

**69**  **70**

**71**  **72**

**73**  **74**

**75**

**Isoflavonoids**

**76**

**77**  **78**  **79**

**80**  **81**

**82**  **83**   **84**

**Modified Glycosides**

**85**

**86**  **87** **88**

**89** **90**

**91** **92**

**Polyketides**

**93** **94**  **95** **96**

**Terpenoids**

**97** R_1_=CH_3_; **98** R_1_=CH_2_OH

**99** R_1_=CH_3_; **100** R_1_=CH_2_OH **101**

**102** **103**

**104**  **105** **106**

**107**  **108**

**109**

**110** R_1_=OH,**111** R_1_=H

**112** **113**

**114**

**115** **116**  **117** **118**  **119** **120**

**121** **122** **123**

**124** **125** **126** **127** **128**

**129** **130****131**

**132**  **133**

**Saponins**

**134**

**135** **136** **137**

**138**

**139**  **140**

**141**  **142****143**

**Other Compounds**

**144** **143**

**144** **145**

**146**

**147** **148** **149**

**150**

**151** **152**  **153** **154**

**155** **156** **157** **158** **159****160****161**  **162** **163**

**164**  **165** **166**

**167** **168**

**169** **170**  **171** **172** **173** **174**

**175**  **176**

**201** R_1_ = OH, R_2_ = CH_3_

**202** R_1_ = OCH_3_, R_2_ = CH_3_

**203** R_1_ = OCH_3_, R_2_ = CH_3_, R_3_ = OCH_3_

**205** R_1_ = OCH_3_, R_2_ = H

**208**  **209** **210**

**211**  **212 213**
