# Supplementary material for: A review and in silico screening of plant-derived snake venom/toxin inhibitors: ADMET, drug-likeness, and medicinal chemistry profiling
Source: PLoS Negl Trop Dis. 2025 Oct 9;19(10):e0013579. doi: 10.1371/journal.pntd.0013579 (PMC12527186; doi:10.1371/journal.pntd.0013579)
Supplement: S1 Table — (DOCX) [file pntd.0013579.s002.docx]

**Table S1: Plants Derived Compounds with Antivenom Properties**

|  | **Compound** | **Plant Isolated** | **Suppressed Activities** | **Venom/toxin** | **Snake Type** | **Reference** |
| --- | --- | --- | --- | --- | --- | --- |
|  |  |  |  |  |  |  |
| **1** | Schumanniofoside | *Schumanniophyton magnificum^TS^* | lethality | Venom | *Naja melanoleuca* | (1) |
| **2** | 12-methoxy-4 methylvoachalotine | *Tabernaemontana catharinensis^RT^* | lethality,myotoxicity | Venom | *Crotalus durissus* | (2) |
| **3** | 22 α,23 α epoxy-solanida 1,4,9-trien-3-one | *Solanum campaniforme^L^* | myotoxicity, hemorrhagic and skinnecrosis | Venom | *Bothrops pauloensis* | (2) |
| **4** | 22 α,23 α-epoxy-solanida 1,4-dien-3-one | *S.companiforme^L^* | myotoxicity, hemorrhagic and skin necrosis | Venom | B.pauloensis | (2) |
| **5** | 22β,23 β -epoxy-solanida 1,4-dien-3-one | *S.companiforme^L^* | hemorrhagic, increase of creatine kinase | Venom | *B.pauloensis* | (2) |
| **6** | 22 α,23 α -epoxy-solanida-4 en-3-one | *S.companiforme^L^* | hemorrhagic, necrotic, increase of creatine kinase | Venom | *B.pauloensis* | (2) |
| **7** | 22β,23β -epoxy-solanida-4 en-3-one | *S.companiforme^L^* | necrotic, increase of creatine kinase | Venom | *B.pauloensis* | (2) |
| **8** | (E)-N-[8′(4-hydroxyphenyl) ethyl]-22 α,23 α -epoxy solanida-1,4,9-trien-3-imine | *S.companiforme^L^* | necrotic | Venom | *B.pauloensis* | (3) |
| **9** | (Z)-N-[8′(4-hydroxyphenyl) ethyl]-22 α,23 α -epoxy solanida-1,4-dien-3-imine | *S.companiforme^L^* | proteolytic, hemorrhagic, necrotic | Venom | *B.pauloensis* | (3) |
| **10** | Berberine | *Aristolochia indica^R^, A.sprucei^S^* | enzimatic, competitive inhibitor | PLA_2_ | *Daboia russelii* | (4) |
| **11** | Aristolochic acid | *A. indica^R^* | enzimatic, edematogenous, myotoxic, muscle damage, hemolytic | Venom, LAAO, HAase, PLA2, VRV PL-VI (PLA_2_), PrTX-I (PLA_2_) | *D. russelii, D. r.pulchella, Vıpera russelii,B. jararacussu, B.asper, B.pirajai,N.naja* | (4–7) |
| **12** | Hydroxyl aristolochic acid | *A. indica^R^* | enzymatic | LAAO | *D. russelii, N.naja* | (5) |
| **13** | Chloride aristolochic acid | *Hemidesmus indicus^R^* | enzymatic | LAAO | *D. russelii, N.naja* | (5) |
| **14** | 12-Methoxy-Nb-methyl-voachalotine | *Tabernaemontana ^R^ catharinensis* | lethality and myotoxic | Venom, PLA2 | *Crotalus durissus terrificus* | (8) |
| **15** | Piperine | *Piper longum ^L.^* | enzimatic, hemorrhagic, lethality | PLA2 | *Daboia russelii* | (9) |
| **16** | Atropine |  | inhibition of venom-induced neuromuscular blockade |  |  | (10) |
| **17** | Anisodamine |  |  | Venom, HAase and NNH1 |  | (11) |
|  |  |  |  |  |  |  |
|  |  |  |  |  |  |  |
| **18** | 2-hydroxy-4-methoxy benzoic acid | *-* | hemorrhagic, edematogenous, coagulant, lethality, defibrination, inflammation | Venom | *D. russelii, V. russelii, N.kaouthia, Ophiophagushannah, Echis carinatus* | (11–15) |
| **19** | 2-hydroxy-4-methoxy benzaldehyde | *Janakia arayalpatra^SS^* | enzimatic, hemorrhagic, lethality | Venom, PLA_2_ | *D. russelii, V. russelii, N.kaouthia* | (14,16) |
| **20** | 3-methoxy benzaldehyde | *J.arayalpatra^SS^* | lethality, enzimatic | Venom, PLA_2_ | *D. russelii, N.kaouthia* | (16) |
| **21** | 3,4-dihydroxy benzaldehyde | *J.arayalpatra^SS^* | lethality, hemorrhagic, enzimatic | Venom, PLA_2_ | *D. russelii, N.kaouthia* | (16) |
| **22** | 2-hydroxy-3-methoxy benzaldehyde | *J.arayalpatra^SS^* | lethality, hemorrhagic | Venom | *D. russelii, N.kaouthia* | (16) |
| **23** | 2-hydroxy-3 methoxybenzylalcohol | *J.arayalpatra^SS^* | enzimatic, desfibrogenation, coagulant, lethality | Venom | *D. russelii, N.kaouthia* | (16) |
| **24** | Anisic acid | *H. indicus^R^* | lethality, defibrinogenation, hemorrhagic, edematogenous | Venom, VRV-PL-VIIIa (PLA_2_) | *V. russelii, E.carinatus, N.kaouthia, O.hannah* | (15,17) |
| **25** | Salicylic acid | *H. indicus^R^* | hemorrhagic | Venom | *V. russelii, E.carinatus, N.kaouthia, O.hannah* | (15) |
| **26** | Gallic acid | **-** | proteolytic, hemorrhagic, edematogenous, | Venom | *D. russelii* | (18,19) |
| **27** | Vanillic acid | **-** | enzymatic, coagulant | 5’AMP | *N.naja* |  |
|  |  |  |  |  |  |  |
| **28** | Rosmarinic acid | *Cordia verbenacea^L^, Argusia argentea^L^* | enzimatic, edematogenous, myotoxicity, hemorrhagic, hydrolytic fibrogenolysis | Venom, BthTX-I, BthTX-II (PLA_2_), SVMP | *B. jararacussu, Trimeresurus flavoviridis, Gloydius blomhoffii, Bitisarietans, C.atrox, Agkistrodon bilineatus, Deinagkistrodon acutus,Protobothrops flavoviridis* | (20–22) |
| **29** | Caffeic acid | *-* | Reduction in plasma fibrogen, myotoxic, muscle damage, cytotoxicity | Venom, SVMP,PrTX-I (PLA_2_) | *B.pirajai,C.d.cumanensis* | (7,19) |
| **30** | Triacontylp-coumarate (PCT) | *Bombacopsis glabra^RB^* | Reduction in plasma fibrogen, coagulant, myotoxicity | Venom,SVMP | *Bothropoides pauloensis* | (23) |
| **31** | p-coumaricacid | *-* | enzimatic | PLA_2_ | *D. r.pulchella* | (24) |
| **32** | Chlorogenic acid | *Vernonia condensata* | lethality, enzimatic | Venom, PLA_2_ | *B. jararaca,D. russelii* | (25) |
| **33** | Cynarin | *Cynara scolymus* | lethality | Venom | *B. jararaca* | (25) |
| **34** | Ferulicacid | *Baccharis uncinella^A^* | enzimatic, edematogenous, cytotoxicity | PLA_2_ | *C.d. terrificus, C.d. cumanensis* | (19,26) |
| **35** | Propylgallate | *-* | enzimatic, cytotoxicity, myotoxicity | Venom | *C.d.cumanensis* | (19) |
|  |  |  |  |  |  |  |
| **36** | Tannic acid | *-* | enzimatic, hemorrhagic, lethality,creatine kinase reduction | Venom, HAase | *C.adamenteus* | (27) |
| **37** | Ellagic acid | *Casearia sylvestris^L^* | enzimatic, edematogenous, myotoxicity | Venom, PLA_2_ | *B. jararacussu* | (28) |
| **38** | 3`-O-methylellagic acid | *C.sylvestris^L^* | edematogenous, myotoxicity | Venom, PLA_2_ | *B. jararacussu* | (28) |
| **39** | Casuarictin | *Laguncularia racemosa^L^* | edematogenous, myonecrosis | PLA_2_ | *C.d. terrificus* | (29) |
| **40** | Pentagalloylglucopyranose | *Mangifera indica^SK^* | enzimatic | PLA_2_, HAase, LAAO | *Calloselasma rhodostoma, N.n.kaouthia* | (30) |
|  |  |  |  |  |  |  |
| **41** | Umbelliferone | *-* | edematogenous, inflammatory, platelet aggregation | Venom, PLA_2_ | *B.neuwiedi* | (31) |
| **42** | (+)-alternamin | *Murraya alternans^A^* | hemorrhagic | Venom | *T.flavoviridis* | (32) |
| **43** | Bergapten | *Dorstenia brasiliensis* | lethality | Venom | *B. jararaca* | (25) |
|  |  |  |  |  |  |  |
| **44** | Hesperetin | *Citrussinensis^P^* | enzimatic | SVSP | *C.simus* | (33,34) |
| **45** | Pinostrobin | *Renealmiaalpinia^L^* | enzimatic, myotoxicity, proteolytic, hemolytic, coagulant | Venom,  PLA_2_ | *C.d.cumanensis,B.asper* | (35) |
| **46** | Hesperidin | *C.sinensis* | enzimatic, hemorrhagic, lethality | Venom, HAase | *C.adamenteus* | (25) |
| **47** | Apigenin | *-* | enzimatic, hemorrhagic, lethality | Venom, HAase | *C.adamenteus,C.atrox,* *N.n. sputatrix* | (27,36) |
| **48** | Luteolin | *-* | enzimatic, hemorrhagic, lethality | Venom, HAase | *C.adamenteus ,C.atrox, N.n.sputatrix* | (36) |
| **49** | Pectolinarigenin | *A. integrifolia^L^* | enzimatic | PLA_2_, HAase | *B.atrox* | (37) |
| **50** | Hispidulin | *A. integrifolia^L^* | enzimatic | PLA_2_, HAase | *B.atrox* | (37) |
| **47** | Morelloflavone | *Garciniamadruno^A^* | enzimatic, coagulant, myotoxicity, edematogenous | PLA_2_ | *C.d.cumanensis* | (38) |
| **48** | Quercetin | *Phyllanthusklotzschianus^A^, Morusnigra^L^, Erythroxylum ovalifolium^L^, E.subsessile^S^* | enzimatic, edematogenous, proteolytic, lethality | Venom, HAase | *B. jararacussu, Lachesis muta,B. jararaca,N.naja* | (35,39–41) |
| **49** | Kaempferol | *-* | enzimatic, hemorrhagic, lethality | Venom, HAase | *C.adamanteus, C.atrox, N.n.sputatrix* | (27,36) |
| **50** | Fisetin | *-* | enzimatic | PLA_2_ | *C.atrox* | (42) |
| **51** | Myricetin | *-* | enzimatic, proteolytic, hemorrhagic | Venom, PLA_2_ | *C.atrox,B.atrox* | (42,43) |
| **52** | Quercitrin | *-* | enzimatic | PLA_2_ | *C.atrox* | (42) |
| **53** | Quercetin-3-O rhamnoside | *Euphorbiahirta^WP^* | enzimatic, hemolytic, lethality, edematogenous | Venom, PLA_2_, HAase | *N.naja* | (44) |
| **54** | Rutin | *E.ovalifolium^S^, E.subsessile^S^* | hemorrhagic | Venom | *L.muta* | (42,45) |
| **55** | Taxifolin | *-* | enzimatic | PLA_2_ | *C.atrox* | (42) |
| **56** | Catechin | *Scolopiachinensis^S^* | enzimatic | PDE-I | *-* | (46) |
| **57** | Gallocatechin | *Schizolobiumparahyba^L^* | hemorrhagic, fibrogenolytic, myotoxicity | Venom, SVMP, PLA_2_ | *B. jararacussu, B.neuwiedi, B.alternatus* | (47) |
| **58** | Epigallotechin gallate | *-* | enzimatic, cytotoxicity | PLA_2_ | *C.d.cumanensis* | (19) |
| **59** | Butein | *Buteamonosperma* | enzimatic | Daboxina (PLA_2_) | *D. russelii* | (48) |
| **60** | Primetin | *Primula denticulata^S^* |  | Toxin |  | (49) |
| **61** | Myricetin-3-O-Glucoside | *Schizolobium^L^*  *parahyba* | hemorrhagic, fibrogenolytic, myotoxicity | Venom |  | (50) |
| **62** | Hypolaetin-8-glucoside | *Sideritis mugronensis ^RB^* | morthality, myotoxicity, proteolytic, enzimatic | PLA_2_ |  | (51) |
| **63** | Isoquercitrin | *Solanum incanum^R^* | Enzymatic,edematogenous, myotoxicity | Venom |  | (52) |
| **64** | Patuletin-3-O-α-L-rhamnopyranosyl-7-O-α-L-rhamnopyranoside | *Bryophyllum pinnatum^L^* | Enzymatic,edematogenous, myotoxicity |  |  | (53) |
| **65** | Iridin |  |  | Venom |  | (54) |
| **66** | Quercetin-3-O-sophoroside | *Cissampelos pareira^A^* |  | Venom, PLA_2_ | *Bothrops diporus* | (55) |
|  |  |  |  |  |  |  |
| **67** | Harpalycin 2 | Harpalycebrasiliana^L^ | Enzymatic,edematogenous, myotoxicity | Venom, PLA_2_,PrTX III | *B.pirajai* | (56,57) |
| **68** | 7,8,3’-trihydroxy-4’ methoxyisoflavone | Dipteryxalata^S^ | myotoxicity, neuromuscular | Venom, BthTX-I | *B. jararacussu* | (58) |
| **69** | Edunol | Brongniartia podalyrioides^R^, H.brasiliana^R^ | morthality, myotoxicity, proteolytic, enzimatic | Venom, PLA_2_ | *B.atrox,B. jararacussu* | (59,60) |
| **70** | Bioisostere | H.brasiliana^SS^ | myotoxicity | Venom | *B. jararacussu* | (59) |
| **71** | CabenegrinsA-I | Annonacrassiflora^R^ | lethality | Venom | *B.atrox* | (61) |
| **72** | CabenegrinsA-II | A.crassiflora^R^ | lethality | Venom | *B.atrox* | (61) |
| **73** | Wedelolactone | E. cliptaprostrate^SY^ | proteolytic, myotoxicity | Venom, PLA_2_ | *B. jararacussu* | (62) |
| **74** | Analogue of wedelolactone | E.prostrate^SY^ | myotoxicity | Venom | *B. jararacussu* | (62) |
| **75** | Demethylwedelolactone | E. alba^R^ | myotoxicity | PLA_2_ | *C.d. terrificus, B. jararacussu* | (40) |
| **76** | Pterocarpan |  |  | Venom |  |  |
|  |  |  |  |  |  |  |
| **77** | 2-(6-benzoyl- β -glucopyranosyloxy)-7-(1 α,2 α,6 α -trihydroxy 5-oxocyclohex-3-enoyl)-5-hydroxybenzylalcohol | *Bennettiodendron leprosipes^B^, Flacourtia ramontchi ^BC^* | enzimatic | PDE-I | *-* | (63) |
| **78** | Homaloside D | *B. leprosipes^B^, F. ramontchi^BC^* | enzimatic | PDE-I | *-* | (63) |
| **79** | Itoside B | *Itoa orientalis^B^* | enzimatic | PDE-I | *-* | (63) |
| **80** | Itoside F | *I.orientalis^B,BC^* | enzimatic | PDE-I | *-* | (63) |
| **81** | Scolochinenoside C | *S.chinensis^S^* | enzimatic | PDE-I | *-* | (46) |
| **82** | Scoloposide C | *S.chinensis^S^* | enzimatic | PDE-I | *-* | (46) |
| **83** | Benzoylsalireposide | *Symplocos racemosa^WP^* | enzimatic | PDE-I | *-* | (64) |
| **84** | Salireposide | *S. racemosa^WP^* | enzimatic | PDE-I | *-* | (64) |
|  |  |  |  |  |  |  |
| **85** | Lapachol | **-** | enzimatic | Venom | *B. jararaca,B.atrox* | (65) |
| **86** | Analogueof lapachol | **-** | enzimatic | Venom | *B. jararaca,B.atrox* | (65) |
| **87** | Isohemigossypolone | *Pachiraaquatica^R^* | injury | Venom | *B.pauloensis,B.moojeni* | (66) |
| **88** | Ehretianone | *Ehretiabuxifolia^SB^* | morthality | Venom | *E.carinatus* | (67) |
| **89** | Melanin | *Thea sinensisLinn.^BT^* | enzimatic | Venom, PLA_2_ | *A.contortrix laticinctus* | (68) |
| **90** | 11-deoxoglycyrrhetinicacid | *Clematisgouriana^R^* |  | Venom, PLA_2_ | *,A. halys blomhoffii,C.atrox* | (68) |
|  |  |  |  |  |  |  |
| **91** | Ar-turmerone | *Curcumalonga^R^* | hemorrhagic, lethality, edematogenous, necrosis | Venom | *D. r.puchella, B. jararaca,C.d. terrificus* | (37,69) |
| **92** | (E)-17-ethyliden-labd-12-ene 15,16-dial (labdanedialdehyde) | *C.zedoaroides^RZ^* | lethality | Venom | *B.aspe rand B.atrox* | (70) |
| **93** | Labdanelactone | *C.antinaia^RZ^, C. contravenenum^RZ^, C. zedoaroides^RZ^* | lethality | Venom | *O.hannah* | (71) |
| **94** | Labdanetrialdehyde | *C.antinaia^RZ^, C. contravenenum^RZ^,C. zedoaroides^RZ^* | diaphragmatic, neurotoxic | Venom | *O.hannah* | (71) |
| **95** | Neo-clerodane | *B. trimera^A^* | hemorrhagic | Venom, SVMP | *O.hannah* | (72) |
| **96** | Lupeol acetate | *H. indicus^R^* | lethality, hemorrhagic, desfibrogenation, edematogenous, enzymatic, cardiotoxicity, neurotoxicity | Venom, PLA_2_ | *B.neuwiedi, B. jararacussu* | (73) |
| **97** | 11-deoxoglycyrrhetinicacid | *Clematisgouriana^R^* | enzimatic | PLA_2_ | *D. russelii, N.kaouthia* | (74) |
| **98** | SID249494135 | *C.gouriana^R^* | enzimatic | PLA_2_ | *D. russelii, N.kaouthia* | (74) |
| **99** | Oleanolicacid | *B.uncinella^A^* | enzimatic, proteolytic, hemorrhagic, edematogenous | PLA_2_, SVMP | *D.russelii, N.naja, B.atrox, C.d.terrificus* | (26,43,75) |
| **100** | Betulinicacid | *-* | proteolytic | Venom | *B.atrox* | (43) |
| **101** | Ursolic acid | *B.uncinella^A^* | proteolytic, enzimatic, edematogenous | Venom, PLA_2_ | *B.atrox,C.d. terrificus* | (76) |
| **102** | Quinovic acid | *Mitragyna stipulosa^B^* | enzimatic | PDE-I | *-* | (76) |
| **103** | Quinovinglycoside C | *M.stipulosa^B^* | enzimatic | PDE-I | *-* | (77) |
| **104** | Arjunolicacid | *Combretum leprosum^R^* | lethality,hemorrhagic,myotoxicity | Venom | *B. jararacussu, B. jararaca* | (45) |
| **105** | Friedelin | *E.ovalifolium^S^* | hemorrhagic | Venom | *L.muta* | (45) |
| **106** | Lupeol | *E.subsessile^S^* | proteolytic, hemolytic, hemorrhagic | Venom | *L.muta* | (78) |
| **107** | Botulin | *Dipteryx alata^B^* | neuromuscular blocked | Venom | *B. jararacussu* | (78) |
| **108** | Lupenone | *D.alata^B^* | Neuromuscular blocked | Venom | *B. jararacussu* | (78) |
| **109** | 28-OH-lupenona | *D.alata^B^* | Neuromuscular blocked | Venom | *B. jararacussu* | (25) |
| **110** | β -amyrin | *Apuleia leiocarpa* | lethality | Venom | *B. jararaca* | (79) |
| **112** | Ikshusterol3-O-glucoside | *C.gouriana^R^* | enzimatic | Venom, PLA_2_ | *N.naja* | (80) |
| **113** | β -sitosterol | *Plucheaindica^R^* | lethality, hemorrhagic, defibrogenation, cardiotoxicity, neurotoxicity, edematogenous, enzimatic | Venom, PLA_2_ | *D. russelii,* | (80) |
| **114** | Stigmasterol | *P. indica^R^* | ethality,hemorrhagic, defibrogenation, cardiotoxicity,neurotoxicity,edematogenous, enzimatic | PLA_2_ | *N.kaouthia* | (24) |
| **115** | Corticosterone | *-* | enzimatic | Venom, PLA_2_ | *D. russelii, N.kaouthia* | (48) |
| **116** | Bakuchiol | *Psoralea corylifolia* | enzimatic,coagulant | PLA_2_ | *D. russelii* | (48) |
| **117** | Bt-CD (7α-hydroxy-3,13-clerodadiene-16,15:18,19 diolide) | *Baccharis trimera*, | proteolytic, hemolytic, hemorrhagic | Venom | *Bothrops neuwiedi* and *Bothrops jararacussu* | (72) |
|  |  |  |  |  |  |  |
| **118** | Bredemeyeroside B | *Bredemeyera floribunda^R^* | lethality | Venom | *B. jararaca* | (81) |
| **119** | Bredemeyeroside D | *B.floribunda^R^* | lethality | Venom | *B. jararaca* | (82) |
| **120** | Glycyrrhizin | *Glycyrrhiza glabra^R^* | coagulation, hidrolytic, platelet aggregation | Venom | *B. jararaca* | (83) |
| **121** | Macrolobin A | *Pentaclethra macroloba^B^* | hemorrhagic,fibrogenolytic | Venom, SVMP | *B.neuwiedi,B. jararacussu* | (84) |
| **122** | Macrolobin B | *P.macroloba^B^* | hemorrhagic,fibrogenolytic | Venom, SVMP | *B.neuwiedi,B. jararacussu* | (84) |
| **123** | Quinovicacid-3-O- α -L-rhamnopyranoside | *Brideliandellensis^B^* | enzimatic | PDE-I | *-* | (85) |
| **124** | Quinovicacid-3-O- β -D-fucopyranoside | *B.ndellensis^B^* | enzimatic | PDE-I | *-* | (85) |
| **125** | Quinovicacid-3-O- β -D-glucopyranosyl (1!4)- β -D fucopyranoside | *B.ndellensis^B^* | enzimatic | PDE-I | *-* | (85) |
|  | Gymnemic acid | *Gymnema sylvestre^L^* |  | Venom | *Naja naja* | (86) |
|  |  |  |  |  |  |  |
| **126** | Mesozygin B | *M.mesozygia^L^* | enzimatic | PDE-I | *-* | (87) |
| **127** | ArtoninI | *M.mesozygia^L^* | enzimatic | PDE-I | *-* | (87) |
| **128** | Resveratrol | *-* | enzimatic | PLA_2_ | *D. r.puchella* | (24) |
| **129** | Gramine | *-* | enzimatic | PLA_2_ | *D. r.puchella* | (24) |
| **130** | Mimosine | *-* | enzimatic,myotoxicity | Venom, HAase | *D. russelii* | (48) |
| **131** | 2-Methylpropylphthalate | *Emblica officinalis^R^* | myotoxicity | Venom | *N.kaouthia,V. russelii* | (88) |
| **132** | Curcumine | *-* | enzimatic | HAase | *N.naja* | (41) |
| **133** | 4-nerolidylcatechol | *Piper umbellatumBC, P .peltatum^BC^* | enzimatic | PLA_2_,SVSP | *B.asper,* *B. jararacussu* | (89) |
| **134** | 1-hydroxytetratriacontan-4-one | *Leucas aspera^L^* | venomaction | Venom | *N.n.naja* | (90) |
| **135** | fatty alcohol 1-hydroxytetratriacontan-4-one | *Leucas aspera^L^* |  | Venom | *N. naja* | (91) |
| **136** | 12-Methoxy-4-Methylvoachalotine (MMV) | *Tabernaemontana catharinensis^RB^* |  | PLA_2_ | *Crotalus durissus terrificus* | (92) |
| **137** | Silybin (I) | *Silybum marianum^L^* |  | venom HAase |  | (93) |
| **138** | Phloretin |  |  | Venom, HAase | *N. naja* | (45) |
| **139** | *n*-Propyl Gallate |  |  | Venom, HAase | *N. naja* | (94) |
| **140** | Butylated Hydroxytoluene (BHT) |  |  | Venom, HAase |  | (95) |
| **141** | Methyl gallate | *Mangifera indica^SK^* |  | Venom | *Calloselasma rhodostoma* and *Naja naja kaouthia* | (96) |
| **142** | α-amyrin | *Pergularia daemia^RB^* |  | Venom |  | (43) |
| **143** | di-iso-butyl phthalate | *Emblica officinalis* (*Phyllanthus emblica*) ^RB^ | coagulation, hidrolytic, platelet aggregation | Venom | *Viper* and *cobra* | (91) |
| **144** | curcuma dialdehyde | *Curcuma zedoaria^L^* |  | Venom | *O hannah* | (97) |
| **145** | 2,4-dimethylhexane | *Tragia involucrata* |  | PLA_2_ |  | (98) |
| **146** | 2,6-dimethylheptane | *Tragia involucrata* |  | PLA_2_ |  | (99) |
| **147** | 2-methylnonane | *Tragia involucrata* |  | PLA_2_ | *Naja naja* | (80) |
| **148** | genistein | *Glycine max* |  | phospholipase-I and RV-PL-V |  | (100) |
| **149** | mimosine | *Mimosa pudica ^L^* |  | HAase | *Vipera russelli* | (101) |
| **150** | Leucasin | *Leucas aspera^L^* |  | PLA_2_ |  | (77) |
| **151** | amenthoflavone | *Byrsonima crassa* |  | venom | *Naja naja* | (41) |
| **152** | Myricetin |  | Hemorrhagic | venom | *Bothrops jararaca* | (102) |
| **153** | anisodamine | *Anisodus tanguticus* | venom-induced microcirculatory | Venom |  | (25) |
| **154** | AIPLAI | *Azadirachta indica* |  | PLA_2_ | *Naja kaouthia* and *Daboia russelii* | (99) |
| **155** | rhamnopyranoside | *Neocarya macrophylla^S^* |  | PLA_2_ | *Naja nigricollis* | (95) |
| **156** | Isoquercitrin | *Schizolobium parahyba^L^* |  |  | *B. jararacussu* and *B. neuwiedi* | (103) |
| **157** | furanoid diterpene lactone | *Aristolochia albida* |  | Venom | *Naja nigricollis* and *Bitis arietans* | (104) |
| **156** | Quinonoid xanthene ehretianone | *Ehretia buxifolia* |  | Venom |  | (105) |
| **158** | HI-RVIF | *Hemidesmus indicus^R^* | lethal, hemorrhagic, coagulant, and anticoagulant | venom | viper | (106) |
| **159** | Alpha-tocopherol |  |  | PLA_2_ |  | (107) |
| **160** | Cardiac Glycosides | *Costus afer^L^* | venom-induced toxicity | Venom |  | (108) |
| **162** | D-Mannitol | *Mimosa pudica* |  | Venom |  | (109) |
| **163** | Polyisoprenylated Benzophenones | *Clusia fluminensis^LFSRB^* |  |  | *Bothrops jararaca* | (8) |
| **164** | Patuletin-3-O-Glycoside | *Bryophyllum pinnatum* |  |  |  | (110) |
| **165** | 2-Hydroxy-4-Methoxy Benzoic Acid | *Hemidesmus indicus* |  |  |  | (111) |
| **166** | D-X-Pinene Camphene | *Citrus* spp. | Anticoagulant, lethality | PLA_2_ | *Naja naja karachiensis, Lachesis muta* | (51) |
| **167** | Ichangin 4-β-Glucopyranoside | *Citrus* spp. | Anticoagulant, lethality | PLA_2_ | *Naja naja karachiensis, Lachesis muta* | (112) |
| **168** | Nomilinic Acid | *Citrus* spp. | Anticoagulant, lethality | PLA_2_ | *Naja naja karachiensis, Lachesis muta* | (94) |
| **169** | Lignan (-)-Cubebin | *Aristolochia* spp^L^ | gelatinolytic, collagenase, peroxidase, and nuclease | PLA_2_ | *Naja naja* , *Bothrops atrox* and *Vipera russelli* | (113) |
| **170** | 2-Mercapto-L-Cysteine | *Allium sativum* L^B^ | hepatoprotective activity | PLA_2,_ | *Naja naja karachiensis, Naja naja karachiensis* | (95) |
| **171** | Sativin I | *Allium sativum* L |  | PLA_2,_ | *Naja naja karachiensis* | (114) (114) |
| **172** | Sativin II | *Allium sativum* L |  | PLA_2,_ | *Naja naja karachiensis* | (115) |
| **173** | 8-Methoxycoumestrol | *Medicago sativa* L | myotoxic | Venom | *Bothrops jararacussu* | (115) |
| **174** | 1-Hydroxytetratriacontane-4-One | *Leucas aspera* Linn |  | Venom | *Naja naja naja* | (116) |
| **175** | 3,4-Dihydroxyphenyllactic Acid | *Cordia verbenacea* | inflammatory and myotoxic | Venom | *Bothrops jararacussu* | (102) |
| **177** | 2-Hexenal | *Bidens pilosa* L |  | Venom | *Dendroaspis jamesoni* and *Echis ocellatus* | (117) |
| **178** | 2-Hexen-1-ol | *Bidens pilosa* L |  | PLA_2,_ | *Dendroaspis jamesoni* and *Echis ocellatus* | (9) |
| **179** | Elixene | *Bidens pilosa* L |  | PLA_2,_ | *Dendroaspis jamesoni* and *Echis ocellatus* | (118) |
| **180** | (−)-Globulol | *Bidens pilosa* L |  | PLA_2,_ | *Dendroaspis jamesoni* and *Echis ocellatus* | (49) |
| **181** | Thujopsene | *Bidens pilosa* L |  | PLA_2,_ | *Dendroaspis jamesoni* and *Echis ocellatus* | (119) |
| **182** | Caftaric Acid |  | neuromuscular blocking | Venom | *Bothrops jararacussu* | (120) |
| **183** | Chicoric Acid |  | neuromuscular block and muscle damage | Venom | *Bothrops jararacussu* | (98) |
| **14** | Triacontyl p-Coumarate |  | hemorrhagic | Venom | *Bothrops pauloensis* | (121) |
| **185** | 2-Hydroxy-4-methoxybenzaldehyde | *Janakia arayalpathra* |  | PLA_2_ |  | (107) |
| **186** | 2-Hydroxy-3-methoxybenzylalcohol |  | hemorrhagic |  |  | (106) |
| **187** | 2-OH-4-MeO Benzaldehyde | *Janakia arayalpathra* | hemorrhagic | PLA_2_ |  | (105) |
| **188** | Coumestrol | *Eclipta prostrata* |  | PLA_2_ | *Bothrops jararacussu* | (104) |
| **189** | Iso Butyl Phthalate |  | hemorrhagic | Venom |  | (108) |
| **190** | Dimercaprol |  | hemorrhagic | Venom |  | (122) |
| **191** | Manoalide | *Curcuma longa* | gelatinolytic, collagenase, peroxidase, and nuclease | PLA_2_ | Cobra | (123) |
| **192** | C20 Dialdehyde | *Curcuma zedoaroides* | hemorrhagic |  | King cobra | (123) |
| **193** | Protocatechuic acid |  | hemolytic | PLA_2_ | *Naja naja karachiensis* | (58) |
| **194** | d-α-pinene | *Citrus limon^R^* | coagulant effects | PLA_2_ | *N. naja karachiensis, Lachesis muta* | (118) |
| **197** | camphene | *Citrus limon^R^* | coagulant effects | PLA_2_ | *N. naja karachiensis, Lachesis muta* | (124) |
| **198** | d-Limonene | *Citrus limon* L | coagulant effects | PLA_2_ | *Naja naja karachiensis, Lachesis muta* | (120) |
| **199** | Ichangin-4-β-glucopyranoside | *C. limon* | hemorrhagic | PLA_2_ | *Naja naja karachiensis, Lachesis muta* | (125) |
| **200** | 7-α-H-Cyclopenta[a]cyclopropa[f]cycloundecene-2,4,7,7a,10,11-hexol | *Cynodon dactylon* | hemolytic activity | Venom | *Naja naja* | (112) |
| **201** | α-D-Glucopyranoside, O-α-D-glucopyranosyl-(1→3)-α-D-fructofuranosyl | *C. dactylon^R^* | hemolytic activity | Venom | *N. naja* | (111) |
| **202** | 9-Octadecenoic Acid (2-phenyl-1,3-dioxolan-4-yl)methyl Ester | *C. dactylon* | hemolytic activity | Venom | *N. naja* | (126) |
| **203** | 9,10-Secocholesta-5,7,10(19)-triene-1,3-diol,25-[(trimethylsilyl)oxy] (3α,5Z,7E) | *C. dactylon^R^* | hemolytic activity | Venom | *N. naja* | (110) |
| **204** | Octasiloxane, 1,1,3,3,5,5,7,7,9,9,11,11,13,13,15,15-hexadecamethyl | *C. dactylon^R^* | hemolytic activity | Venom | *N. naja* | (93) |
| **205** | Oximino-2,7-diethoxyfluorene | *C. dactylon^R^* | hemolytic activity | Venom | *N. naja* | (127) |
| **206** | Hexadecanoic acid, 1-(hydroxymethyl)-1,2-ethanediyl ester | *C. dactylon^R^* | hemolytic activity | Venom | *N. naja* | (25) |
| **207** | Hexasiloxane, 1,1,3,3,5,5,7,7,9,9,11,11-dodecamethyl | *C. dactylon^R^* | hemolytic activity | Venom | *N. naja* | (128) |
| **208** | Heptasiloxane, 1,1,3,3,5,5,7,7,9,9,11,11,13,13-tetradecamethyl | *C. dactylon^R^* | neuromuscular block and muscle damage | Venom |  | (129) |
| **209** | Estra-1,3,5(10)-trien-17-α-ol | *C. dactylon^R^* | hemorrhagic | Venom |  | (121) |
| **210** | 3,3′,5′-Trimethoxy-4,5-methylenedioxy dihydrostilbene | *Indigofera capitata* | hemorrhagic | PLA2 | *Naja nigricollis* | (130) |
| **211** | 3,5-Dimethoxy-4-hydroxyphenylacetic acid | *A. parvifolia* | hemorrhagic | PLA_2_ |  | (7) |
| **212** | Acetylmarinobufogenin |  | hemorrhagic | PLA_2_ |  | (131) |
| **213** | γ-Sitosterol |  | neuromuscular block and muscle damage | PLA2 |  | (132) |

L, leaves; TS, tendershoots; R, roots; S,stem; SS, semi synthetic; RB, rootbark;A,aerial;SK,seedkernels, A,aerial;P,peels;SY,synthesis,

SB,stembark;R, roots;S,stem;WP,wholeplant;B,bark;BC,branches;BT,blacktea RZ, rhizomes

Hyaluronidase: HAase; Laminoacidoxidase: LAAO;PhospholipaseA2:PLA2;5’nucleotidase:5’AMP;;Metalloproteinase:SVMP. L, PhosphodiesteraseI,PDE-I; Serineprotease,SVSP.

**References**

1. Batina Adelia C.O.; Veronese, Elen L.G.; Lavrador, Marco A.S.; Giglio, José R; Pereira, Paulo S; Dias, Diones A; França, Suzelei C; Sampaio, Suely V M de FCC. Inhibition of the Lethal and Myotoxic Activities of Crotalus durissus terrificus Venom by Tabernaemontana catharinensis: Identification of One of the Active Components. Planta Med [Internet]. 2000;66(05):424–8. Available from: http://www.thieme-connect.com/products/ejournals/abstract/10.1055/s-2000-8577

2. Silveira ER, Pessoa L, Jeane R, Jorge B, Ximenes RM, Serra H, et al. Antiophidic Solanidane Steroidal Alkaloids from Solanum campaniforme. 2011;

3. Torres MCM, Jorge RJB, Ximenes RM, Alves NTQ, Santos JV de A, Marinho AD, et al. Solanidane and iminosolanidane alkaloids from Solanum campaniforme. Phytochemistry. 2013;96:457–64.

4. Chandra DN, Prasanth GK, Singh N, Kumar S, Jithesh O, Sadasivan C, et al. Identification of a novel and potent inhibitor of phospholipase A2 in a medicinal plant: crystal structure at 1.93 Å and Surface Plasmon Resonance analysis of phospholipase A2 complexed with berberine. Biochim Biophys Acta (BBA)-Proteins Proteomics. 2011;1814(5):657–63.

5. Bhattacharjee P, Bera I, Chakraborty S, Ghoshal N, Bhattacharyya D. Aristolochic acid and its derivatives as inhibitors of snake venom L-amino acid oxidase. Toxicon. 2017;138:1–17.

6. Dhananjaya BL, Gowda T V, JM D ‘Souza C. Evidence for existence of venom 5′ nucleotidase in multiple forms through inhibition of concanavalin A. Cell Biochem Funct. 2010;28(7):620–2.

7. Fernandes CAH, Cardoso FF, Cavalcante WGL, Soares AM, Dal-Pai M, Gallacci M, et al. Structural basis for the inhibition of a phospholipase A2-like toxin by caffeic and aristolochic acids. PLoS One. 2015;10(7):e0133370.

8. Gomes A, Das R, Sarkhel S, Mishra R, Mukherjee S, Bhattacharya S, et al. Herbs and herbal constituents active against snake bite. 2010;

9. Shenoy PA, Nipate SS, Sonpetkar JM, Salvi NC, Waghmare AB, Chaudhari PD. Anti-snake venom activities of ethanolic extract of fruits of Piper longum L.(Piperaceae) against Russell’s viper venom: characterization of piperine as active principle. J Ethnopharmacol. 2013;147(2):373–82.

10. Kadir MF, Karmoker JR, Alam R, Jahan SR, Mahbub S, Mia MMK. Ethnopharmacological Survey of Medicinal Plants Used by Traditional Healers and Indigenous People in Chittagong Hill Tracts , Bangladesh , for the Treatment of Snakebite. 2015;2015.

11. Alam MI, Gomes A. Viper venom-induced inflammation and inhibition of free radical formation by pure compound (2-hydroxy-4-methoxy benzoic acid) isolated and purified from anantamul (Hemidesmus indicus R. BR) root extract. Toxicon. 1998;36(1):207–15.

12. Alam MI, Auddy B, Gomes A. Isolation, purification and partial characterization of viper venom inhibiting factor from the root extract of the Indian medicinal plant sarsaparilla (Hemidesmus indicus R. Br.). Toxicon. 1994;32(12):1551–7.

13. Shuaib M, Samad A, Alam S, Siddiqui ST. Why adopting cloud is still a challenge?—a review on issues and challenges for cloud migration in organizations. Ambient Commun Comput Syst RACCCS-2018. 2019;387–99.

14. Nargotra A, Sharma S, Alam MI, Ahmed Z, Bhagat A, Taneja SC, et al. In silico identification of viper phospholipaseA2 inhibitors: validation by in vitro, in vivo studies. J Mol Model. 2011;17:3063–73.

15. Alam MI, Gomes A. An experimental study on evaluation of chemical antagonists induced snake venom neutralization. Indian J Med Res. 1998;107:142.

16. Alam MI, Alam MA, Alam O, Nargotra A, Taneja SC, Koul S. Molecular modeling and snake venom phospholipase A2 inhibition by phenolic compounds: Structure–activity relationship. Eur J Med Chem. 2016;114:209–19.

17. Uma B, Gowda TV. Molecular mechanism of lung hemorrhage induction by VRV-PL-VIIIa from Russell’s viper (Vipera russelli) venom. Toxicon. 2000;38(8):1129–47.

18. H Mahadeswaraswamy Y, S Kumar M, J Gowtham Y, Nagaraju S, S Girish K, Kemparaju K. The polyphenol 3, 4, 5-tri-hydroxy benzoic acid inhibits Indian Daboia russelli venom and its hemorrhagic complex induced local toxicity. Curr Top Med Chem. 2011;11(20):2520–30.

19. Pereanez JA, Nunez V, Patino AC, Londono M, Quintana JC. Inhibitory effects of plant phenolic compounds on enzymatic and cytotoxic activities induced by a snake venom phospholipase A2. Vitae. 2011;18(3):295–304.

20. Aung HT, Furukawa T, Nikai T, Niwa M, Takaya Y. Contribution of cinnamic acid analogues in rosmarinic acid to inhibition of snake venom induced hemorrhage. Bioorg Med Chem. 2011;19(7):2392–6.

21. Aung HT, Nikai T, Komori Y, Nonogaki T, Niwa M, Takaya Y. Biological and pathological studies of rosmarinic acid as an inhibitor of hemorrhagic Trimeresurus flavoviridis (habu) venom. Toxins (Basel). 2010;2(10):2478–89.

22. Aung HT, Nikai T, Niwa M, Takaya Y. Rosmarinic acid in Argusia argentea inhibits snake venom-induced hemorrhage. J Nat Med. 2010;64:482–6.

23. Mendes MM, Vieira S, Gomes MSR, Paula VF, Alcântara TM, Homsi-Brandeburgo MI, et al. Triacontyl p-coumarate: An inhibitor of snake venom metalloproteinases. Phytochemistry. 2013;86:72–82.

24. Shukla PK, Gautam L, Sinha M, Kaur P, Sharma S, Singh TP. Structures and binding studies of the complexes of phospholipase A2 with five inhibitors. Biochim Biophys Acta (BBA)-Proteins Proteomics. 2015;1854(4):269–77.

25. Pereira NA, Pereira BMR, Nascimento MC, Parente JP, Mors WB. Pharmacological screening of plants recommended by folk medicine as snake venom antidotes; IV. Protection against Jararaca venom by isolated constituents1. Planta Med. 1994;60(02):99–100.

26. Zalewski CA, Passero LFD, Melo ASRB, Corbett CEP, Laurenti MD, Toyama MH, et al. Evaluation of anti-inflammatory activity of derivatives from aerial parts of Baccharis uncinella. Pharm Biol. 2011;49(6):602–7.

27. Kuppusamy UR, Das NP. Protective effects of tannic acid and related natural compounds on Crotalus adamenteus subcutaneous poisoning in mice. Pharmacol Toxicol. 1993;72(4–5):290–5.

28. Da Silva SL, Calgarotto AK, Chaar JS, Marangoni S. Isolation and characterization of ellagic acid derivatives isolated from Casearia sylvestris SW aqueous extract with anti-PLA2 activity. Toxicon. 2008;52(6):655–66.

29. Rodrigues CFB, Ferreira MJP, Belchor MN, Costa CRC, Novaes DP, dos Santos Junior AB, et al. Evaluation of the inhibitory potential of casuarictin, an ellagitannin isolated from white mangrove (Laguncularia racemosa) leaves, on snake venom secretory phospholipase A2. Mar Drugs. 2019;17(7):403.

30. Leanpolchareanchai J, Pithayanukul P, Bavovada R, Saparpakorn P. Molecular docking studies and anti-enzymatic activities of Thai mango seed kernel extract against snake venoms. Molecules. 2009;14(4):1404–22.

31. de Oliveira Toyama D, dos Santos Diz Filho EB, Cavada BS, da Rocha BAM, de Oliveira SCB, Cotrim CA, et al. Umbelliferone induces changes in the structure and pharmacological activities of Bn IV, a phospholipase A2 isoform isolated from Bothrops neuwiedi. Toxicon. 2011;57(6):851–60.

32. Min HM, Aye M, Taniguchi T, Miura N, Monde K, Ohzawa K, et al. A structure and an absolute configuration of (+)-alternamin, a new coumarin from Murraya alternans having antidote activity against snake venom. Tetrahedron Lett. 2007;48(35):6155–8.

33. Vander dos Santos R, Grillo G, Fonseca H, Stanisic D, Tasic L. Hesperetin as an inhibitor of the snake venom serine protease from Bothrops jararaca. Toxicon. 2021;198:64–72.

34. Vander dos Santos R, Villalta-Romero F, Stanisic D, Borro L, Neshich G, Tasic L. Citrus bioflavonoid, hesperetin, as inhibitor of two thrombin-like snake venom serine proteases isolated from Crotalus simus. Toxicon. 2018;143:36–43.

35. Gómez-Betancur I, Pereañez JA, Patiño AC, Benjumea D. Inhibitory effect of pinostrobin from Renealmia alpinia, on the enzymatic and biological activities of a PLA2. Int J Biol Macromol. 2016;89:35–42.

36. Kuppusamy UR, Das NP. Inhibitory effects of flavonoids on several venom hyaluronidases. Experientia. 1991;47:1196–200.

37. Nascimento LS, Nogueira-Souza PD, Rocha-Junior JRS, Monteiro-Machado M, Strauch MA, Prado SAL, et al. Phytochemical composition, antisnake venom and antibacterial activities of ethanolic extract of Aegiphila integrifolia (Jacq) Moldenke leaves. Toxicon. 2021;198:121–31.

38. Pereañez JA, Patiño AC, Núñez V, Osorio E. The biflavonoid morelloflavone inhibits the enzymatic and biological activities of a snake venom phospholipase A2. Chem Biol Interact. 2014;220:94–101.

39. Ribeiro AEAS, Soares JMD, Silva HAL, de Souza Wanderley CW, Moura CA, de Oliveira-Junior RG, et al. Inhibitory effects of Morus nigra L.(Moraceae) against local paw edema and mechanical hypernociception induced by Bothrops jararacussu snake venom in mice. Biomed Pharmacother. 2019;111:1046–56.

40. Diogo LC, Fernandes RS, Marcussi S, Menaldo DL, Roberto PG, Matrangulo PVF, et al. Inhibition of snake venoms and phospholipases A2 by extracts from native and genetically modified Eclipta alba: isolation of active coumestans. Basic Clin Pharmacol Toxicol. 2009;104(4):293–9.

41. Girish KS, Kemparaju K. Inhibition of Naja naja venom hyaluronidase by plant-derived bioactive components and polysaccharides. Biochem. 2005;70:948–52.

42. Lindahl M, Tagesson C. Flavonoids as phospholipase A 2 inhibitors: importance of their structure for selective inhibition of group II phospholipase A 2. Inflammation. 1997;21:347–56.

43. Preciado LM, Rey-Suárez P, Henao IC, Pereañez JA. Betulinic, oleanolic and ursolic acids inhibit the enzymatic and biological effects induced by a PI snake venom metalloproteinase. Chem Biol Interact. 2018;279:219–26.

44. Gopi K, Anbarasu K, Renu K, Jayanthi S, Vishwanath BS, Jayaraman G. Quercetin-3-O-rhamnoside from Euphorbia hirta protects against snake Venom induced toxicity. Biochim Biophys Acta (BBA)-General Subj. 2016;1860(7):1528–40.

45. Coriolano de Oliveira E, Alves Soares Cruz R, de Mello Amorim N, Guerra Santos M, Carlos Simas Pereira Junior L, Flores Sanchez EO, et al. Protective effect of the plant extracts of erythroxylum sp. against toxic effects induced by the venom of lachesis muta snake. Molecules. 2016;21(10):1350.

46. Lu Y-N, Chai X-Y, Xu Z-R, Bi D, Ren H-Y, Zhao M, et al. Three new phenolic glycosides and a new triterpenoid from the stems of Scolopia chinensis. Planta Med. 2010;76(04):358–61.

47. Vale HF, M Mendes M, S Fernandes R, R Costa T, IS Hage-Melim L, A Sousa M, et al. Protective effect of Schizolobium parahyba flavonoids against snake venoms and isolated toxins. Curr Top Med Chem. 2011;11(20):2566–77.

48. Devi A, Namsa ND, Doley R. In silico and in vitro neutralization of PLA2 activity of Daboxin P by butein, mimosine and bakuchiol. Int J Biol Macromol. 2020;165:1066–78.

49. Tijjani H, Egbuna C. Medicinal Plants with Antivenom Activities. In: Phytochemistry. Apple Academic Press; 2018. p. 207–56.

50. Ribeiro FF, Junior FJBM, da Silva MS, Scotti MT, Scotti L. Computational and Investigative Study of Flavonoids Active against Trypanosoma cruzi and Leishmania spp. Nat Prod Commun. 2015;10(6).

51. Alcaraz MJ, Hoult JR. Effects of hypolaetin-8-glucoside and related flavonoids on soybean lipoxygenase and snake venom phospholipase A2. Arch Int Pharmacodyn théRapie. 1985;278(1):4–12.

52. Wansi JD, Devkota KP, Tshikalange E, Kuete V. Alkaloids from the Medicinal Plants of Africa [Internet]. Medicinal Plant Research in Africa: Pharmacology and Chemistry. Elsevier Inc.; 2013. 557–605 p. Available from: http://dx.doi.org/10.1016/B978-0-12-405927-6.00014-X

53. Okumu M, Mbaria J, Gikunju J, Mbuthia P, Madadi V, Ochola F. Exploring nature’s antidote: unveiling the inhibitory potential of selected medicinal plants from Kisumu, Kenya against venom from some snakes of medical significance in sub-Saharan Africa. Front Pharmacol. 2024;15:1369768.

54. Ahmed B, Al-Rehaily AJ, Al-Howiriny TA, El-Sayed KA, Ahmad MS. Scropolioside-D2 and harpagoside-B: two new iridoid glycosides from Scrophularia deserti and their antidiabetic and antiinflammatory activity. Biol Pharm Bull. 2003;26(4):462–7.

55. Gimenes SNC, Sachett JAG, Colombini M, Freitas-de-Sousa LA, Ibiapina HNS, Costa AG, et al. Observation of bothrops atrox snake envenoming blister formation from five patients: Pathophysiological insights. Toxins (Basel). 2021;13(11):800.

56. Ximenes RM, Alves RS, Pereira TP, Araújo RM, Silveira ER, Rabello MM, et al. Harpalycin 2 inhibits the enzymatic and platelet aggregation activities of PrTX-III, a D49 phospholipase A 2 from Bothrops pirajai venom. BMC Complement Altern Med. 2012;12:1–10.

57. Ximenes RM, Rabello MM, Araujo RM, Silveira ER, Fagundes FHR, Diz-Filho EBS, et al. Inhibition of neurotoxic secretory phospholipases A2 enzymatic, edematogenic, and myotoxic activities by harpalycin 2, an isoflavone isolated from Harpalyce brasiliana benth. Evidence‐Based Complement Altern Med. 2012;2012(1):987517.

58. Ferraz MC, Yoshida EH, Tavares RVS, Cogo JC, Cintra ACO, Dal Belo CA, et al. An isoflavone from Dipteryx alata Vogel is active against the in vitro neuromuscular paralysis of Bothrops jararacussu snake venom and bothropstoxin I, and prevents venom-induced myonecrosis. Molecules. 2014;19(5):5790–805.

59. da Silva AJM, Coelho AL, Simas ABC, Moraes RAM, Pinheiro DA, Fernandes FFA, et al. Synthesis and pharmacological evaluation of prenylated and benzylated pterocarpans against snake venom. Bioorg Med Chem Lett. 2004;14(2):431–5.

60. Reyes-Chilpa R, Gómez-Garibay F, Quijano L, Magos-Guerrero GA, Ríos T. Preliminary results on the protective effect of (-)-edunol, a pterocarpan from Brongniartia podalyrioides (Leguminosae), against Bothrops atrox venom in mice. J Ethnopharmacol. 1994;42(3):199–203.

61. Nakagawa M, Nakanishi K, Darko LL, Vick JA. Structures of cabenegrins AI and A-II, potent anti-snake venoms. Tetrahedron Lett. 1982;23(38):3855–8.

62. da Silva Jr NJ, Aird SD. Prey specificity, comparative lethality and compositional differences of coral snake venoms. Comp Biochem Physiol Part C Toxicol Pharmacol. 2001;128(3):425–56.

63. Chai X-Y, Ren H-Y, Xu Z-R, Bai C-C, Zhou F-R, Ling S-K, et al. Investigation of two Flacourtiaceae plants: Bennettiodendron leprosipes and Flacourtia ramontchi. Planta Med. 2009;75(11):1246–52.

64. Ahmad VU, Abbasi MA, Hussain H, Akhtar MN, Farooq U, Fatima N, et al. Phenolic glycosides from Symplocos racemosa: natural inhibitors of phosphodiesterase I. Phytochemistry. 2003;63(2):217–20.

65. Strauch MA, Tomaz MA, Monteiro-Machado M, Cons BL, Patrao-Neto FC, Teixeira-Cruz J da M, et al. Lapachol and synthetic derivatives: in vitro and in vivo activities against Bothrops snake venoms. PLoS One. 2019;14(1):e0211229.

66. Vieira SAPB, Dos Santos BM, Júnior CDS, de Paula VF, Gomes MSR, Ferreira GM, et al. Isohemigossypolone: Antiophidic properties of a naphthoquinone isolated from Pachira aquatica Aubl. Comp Biochem Physiol Part C Toxicol Pharmacol. 2021;245:109028.

67. Selvanayagam ZE, Gnanavendhan SG, Balakrishna K, Rao RB, Sivaraman J, Subramanian K, et al. Ehretianone, a novel quinonoid xanthene from Ehretia buxifolia with antisnake venom activity. J Nat Prod. 1996;59(7):664–7.

68. Hung Y-C, Sava V, Hong M-Y, Huang GS. Inhibitory effects on phospholipase A2 and antivenin activity of melanin extracted from Thea sinensis Linn. Life Sci. 2004;74(16):2037–47.

69. Ferreira LAF, Henriques OB, Andreoni AAS, Vital GRF, Campos MMC, Habermehl GG, et al. Antivenom and biological effects of ar-turmerone isolated from Curcuma longa (Zingiberaceae). Toxicon. 1992;30(10):1211–8.

70. Lattmann E, Sattayasai J, Sattayasai N, Staaf A, Phimmasone S, Schwalbe CH, et al. In-vitro and in-vivo antivenin activity of 2-[2-(5, 5, 8a-trimethyl-2-methylene-decahydro-naphthalen-1-yl)-ethylidene]-succinaldehyde against Ophiophagus hannah venom. J Pharm Pharmacol. 2010;62(2):257–62.

71. Salama R, Sattayasai J, Gande AK, Sattayasai N, Davis M, Lattmann E. Identification and evaluation of agents isolated from traditionally used herbs against Ophiophagus hannah venom. Drug Discov Ther. 2012;6(1):18–23.

72. Januário AH, Santos SL, Marcussi S, Mazzi M V, Pietro RCLR, Sato DN, et al. Neo-clerodane diterpenoid, a new metalloprotease snake venom inhibitor from Baccharis trimera (Asteraceae): anti-proteolytic and anti-hemorrhagic properties. Chem Biol Interact. 2004;150(3):243–51.

73. Chatterjee I, Chakravarty AK, Gomes A. Daboia russellii and Naja kaouthia venom neutralization by lupeol acetate isolated from the root extract of Indian sarsaparilla Hemidesmus indicus R. Br. J Ethnopharmacol. 2006;106(1):38–43.

74. Muthusamy K, Chinnasamy S, Nagarajan S, Sivaraman T, Chinnasamy S. Isolation and characterization of bioactive compounds of Clematis gouriana Roxb. ex DC against snake venom phospholipase A2 (PLA2) computational and in vitro insights. J Biomol Struct Dyn. 2017;35(9):1936–49.

75. Dharmappa KK, Kumar RV, Nataraju A, Mohamed R, Shivaprasad H V, Vishwanath BS. Anti-inflammatory activity of oleanolic acid by inhibition of secretory phospholipase A2. Planta Med. 2009;75(03):211–5.

76. Fatima N, Tapondjou LA, Lontsi D, Sondengam BL, Atta-Ur-Rahman, Choudhary MI. Quinovic acid glycosides from Mitragyna stipulosa-first examples of natural inhibitors of snake venom phosphodiesterase I. Nat Prod Lett. 2002;16(6):389–93.

77. Fernandes FFA, Tomaz MA, El-Kik CZ, Monteiro-Machado M, Strauch MA, Cons BL, et al. Counteraction of Bothrops snake venoms by Combretum leprosum root extract and arjunolic acid. J Ethnopharmacol. 2014;155(1):552–62.

78. Cristina Ferraz M, Aparecida Celestino Parrilha L, Silvia Duarte Moraes M, Amaral Filho J, Carlos Cogo J, Galdino dos Santos M, et al. The effect of lupane triterpenoids (Dipteryx alata Vogel) in the in vitro neuromuscular blockade and myotoxicity of two snake venoms. Curr Org Chem. 2012;16(22):2717–23.

79. Muthusamy K, Chinnasamy S, Nagarajan S, Sivaraman T. Computational and in vitro insights on snake venom phospholipase A2 inhibitor of phytocompound ikshusterol3-O-glucoside of Clematis gouriana Roxb. ex DC. J Biomol Struct Dyn. 2018;36(16):4197–208.

80. Gomes A, Saha A, Chatterjee I, Chakravarty AK. Viper and cobra venom neutralization by β-sitosterol and stigmasterol isolated from the root extract of Pluchea indica Less.(Asteraceae). Phytomedicine. 2007;14(9):637–43.

81. do Rosário Daros M, de Abreu Matos FJ, Parente JP. A new triterpenoid saponin, Bredemeyeroside B, from the roots of Bredemeyera floribunda. Planta Med. 1996;62(06):523–7.

82. Pereira BMR, Daros M do R, Parente JP, Matos FJ de A. Bredemeyeroside D, a novel triterpenoid saponin from Bredemeyera floribunda: a potent snake venom antidote activity on mice. Phyther Res. 1996;10(8):666–9.

83. Assafim M, Ferreira MS, Frattani FS, Guimarães JA, Monteiro RQ, Zingali RB. Counteracting effect of glycyrrhizin on the hemostatic abnormalities induced by Bothrops jararaca snake venom. Br J Pharmacol. 2006;148(6):807.

84. da Silva JO, Fernandes RS, Ticli FK, Oliveira CZ, Mazzi M V, Franco JJ, et al. Triterpenoid saponins, new metalloprotease snake venom inhibitors isolated from Pentaclethra macroloba. Toxicon. 2007;50(2):283–91.

85. Mostafa M, Nahar N, Mosihuzzaman M, Sokeng SD, Fatima N, Atta-ur-Rahman, et al. Phosphodiesterase-I inhibitor quinovic acid glycosides from Bridelia ndellensis. Nat Prod Res. 2006;20(7):686–92.

86. Okot DF, Anywar G, Namukobe J, Byamukama R. Medicinal plants species used by herbalists in the treatment of snakebite envenomation in Uganda. Trop Med Health. 2020;48:1–14.

87. Fozing CDA, Ali Z, Ngadjui BT, Choudhary MI, Kapche GDWF, Abegaz BM, et al. Phosphodiesterase I-inhibiting Diels-Alder adducts from the leaves of Morus mesozygia. Planta Med. 2012;78(02):154–9.

88. Sarkhel S, Chakravarty AK, Das R, Gomes A, Gomes A. Snake venom neutralising factor from the root extract of Emblica officinalis Linn. Orient Pharm Exp Med. 2011;11:25–33.

89. Núñez V, Castro V, Murillo R, Ponce-Soto LA, Merfort I, Lomonte B. Inhibitory effects of Piper umbellatum and Piper peltatum extracts towards myotoxic phospholipases A2 from Bothrops snake venoms: isolation of 4-nerolidylcatechol as active principle. Phytochemistry. 2005;66(9):1017–25.

90. Venkatesan C, Sarathi M, Balasubramanian G, Thomas J, Balachander V, Babu VS, et al. Antivenom activity of triterpenoid (C34H68O2) from Leucas aspera Linn. against Naja naja naja venom induced toxicity: Antioxidant and histological study in mice. Hum Exp Toxicol. 2014;33(4):336–59.

91. Maria Gutierrez J, León G, Lomonte B, Angulo Y. Antivenoms for snakebite envenomings. Inflamm Allergy-Drug Targets (Formerly Curr Drug Targets-Inflammation Allergy)(Discontinued). 2011;10(5):369–80.

92. Adamu M, Uttu AJ, Ajala A, Obansa RM, Madumelu M. A short review on plants used as anti-snake venom. J Chem Rev. 2023;5(3):341–52.

93. Sajevic T, Leonardi A, Križaj I. Haemostatically active proteins in snake venoms. Toxicon. 2011;57(5):627–45.

94. Halilu EM, October N, Ugwah-Oguejiofor CJ, Jega AY, Nefai MS. Anti-snake venom and analgesic activities of extracts and betulinic and oleanolic acids isolated from Parinari curatellifolia. J Med plants Econ Dev. 2020;4(1):1–8.

95. Omale S, Auta A, Amagon KI, Ighagbon M V. Anti-snake venom activity of flavonoids from the root bark extract of Parinari curatellifolia in Mice. IJPR. 1955;

96. Gómez-Betancur I, Gogineni V, Salazar-Ospina A, León F. Perspective on the therapeutics of anti-snake venom. Molecules. 2019;24(18):3276.

97. Omara T, Kagoya S, Openy A, Omute T, Ssebulime S, Kiplagat KM, et al. Antivenin plants used for treatment of snakebites in Uganda: ethnobotanical reports and pharmacological evidences. Trop Med Health. 2020;48:1–16.

98. Soares AM, Ticli FK, Marcussi S, Lourenco M V, Januario AH, Sampaio S V, et al. Medicinal plants with inhibitory properties against snake venoms. Curr Med Chem. 2005;12(22):2625–41.

99. Fox JW, Serrano SMT. Insights into and speculations about snake venom metalloproteinase (SVMP) synthesis, folding and disulfide bond formation and their contribution to venom complexity. FEBS J. 2008;275(12):3016–30.

100. Bickler PE. Amplification of snake venom toxicity by endogenous signaling pathways. Toxins (Basel). 2020;12(2):68.

101. Makhija IK, Khamar D. Anti-snake venom properties of medicinal plants. Der Pharm Lett. 2010;2(5):399–411.

102. H Mahadeswaraswamy Y, Manjula B, Devaraja S, S Girish K, Kemparaju K. Daboia russelli venom hyaluronidase: purification, characterization and inhibition by β-3-(3-hydroxy-4-oxopyridyl) α-amino-propionic Acid. Curr Top Med Chem. 2011;11(20):2556–65.

103. Lauridsen LP, Laustsen AH, Lomonte B, Gutiérrez JM. Toxicovenomics and antivenom profiling of the Eastern green mamba snake (Dendroaspis angusticeps). J Proteomics. 2016;136:248–61.

104. Dauda MM, Dauda G, Abdullahi SM, Sada H, Sule MI, Hassan HS, et al. Isolation and characterization of a novel dihydrostilbene from Indigofera capitata kotschy with antivenin activity against Naja nigricollis venom. Discov Chem. 2025;2(1):43.

105. Maria TAN, CARRANZA M, MALABED R, REYES Y, FRANCO F, LINIS V, et al. Antioxidant, cytotoxic, and anti-venom activity of Alstonia parvifolia Merr. Bark. Asian Pac J Trop Biomed. 2021;460–8.

106. Xiao H, Pan H, Liao K, Yang M, Huang C. Snake Venom PLA2, a Promising Target for Broad‐Spectrum Antivenom Drug Development. Biomed Res Int. 2017;2017(1):6592820.

107. Jorge RJB, Martins RD, Araújo RM, da Silva MA, Monteiro HSA, Ximenes RM. Plants and phytocompounds active against Bothrops venoms. Curr Top Med Chem. 2019;19(22):2003–31.

108. de Sousa Ferreira S, da Silva DP, Torres-Rêgo M, da Silva-Júnior AA, de Freitas Fernandes-Pedrosa M. The potential of phenolic acids in therapy against snakebites: A review. Toxicon. 2022;208:1–12.

109. Singh P, Yasir M, Hazarika R, Sugunan S, Shrivastava R. A review on venom enzymes neutralizing ability of secondary metabolites from medicinal plants. J pharmacopuncture. 2017;20(3):173.

110. Gbolade AA. Nigerian medicinal plants with anti-snake venom activity—A review. J Malar Res Phytomedicine. 2021;1(1).

111. Samy RP, Gopalakrishnakone P, Chow VTK. Therapeutic application of natural inhibitors against snake venom phospholipase A2. Bioinformation. 2012;8(1):48.

112. Yusuf AJ, Aleku GA, Bello UR, Liman DU. Prospects and Challenges of Developing Plant‐Derived Snake Antivenin Natural Products: A Focus on West Africa. ChemMedChem. 2021;16(24):3635–48.

113. Nishijima CM, Rodrigues CM, Silva MA, Lopes-Ferreira M, Vilegas W, Hiruma-Lima CA. Anti-hemorrhagic activity of four Brazilian vegetable species against Bothrops jararaca venom. Molecules. 2009;14(3):1072–80.

114. Sakthivel G, Dey A, Nongalleima K, Chavali M, Rimal Isaac RS, Singh NS, et al. In vitro and in vivo evaluation of polyherbal formulation against Russell’s viper and cobra venom and screening of bioactive components by docking studies. Evidence‐Based Complement Altern Med. 2013;2013(1):781216.

115. Kakegawa H, Matsumoto H, Satoh T. Inhibitory effects of hydrangenol derivatives on the activation of hyaluronidase and their antiallergic activities. Planta Med. 1988;54(05):385–9.

116. MS V, More VS, Zameer F, Muddapur U, More SS. Ethnomedicinal plants and isolated compounds against Snake venom activity: A review. 2021;

117. Dharmappa KK, Mohamed R, Shivaprasad H V, Vishwanath BS. Genistein, a potent inhibitor of secretory phospholipase A 2: a new insight in down regulation of inflammation. Inflammopharmacology. 2010;18:25–31.

118. Raghavamma ST V, Rao NR, Rao GD. Inhibitory potential of important phytochemicals from Pergularia daemia (Forsk.) chiov., on snake venom (Naja naja). J Genet Eng Biotechnol. 2016;14(1):211–7.

119. Bhattacharjee P, Bhattacharyya D. Medicinal plants as snake venom antidotes. J Exp Appl Anim Sci. 2013;1(1):156–81.

120. Sebastin Santhosh M, Hemshekhar M, Sunitha K, M Thushara R, Jnaneshwari S, Kemparaju K, et al. Snake venom induced local toxicities: plant secondary metabolites as an auxiliary therapy. Mini Rev Med Chem. 2013;13(1):106–23.

121. Cedro RCA, Menaldo DL, Costa TR, Zoccal KF, Sartim MA, Santos-Filho NA, et al. Cytotoxic and inflammatory potential of a phospholipase A 2 from Bothrops jararaca snake venom. J Venom Anim Toxins Incl Trop Dis. 2018;24:33.

122. Molander M, Nielsen L, Søgaard S, Staerk D, Rønsted N, Diallo D, et al. Hyaluronidase, phospholipase A2 and protease inhibitory activity of plants used in traditional treatment of snakebite-induced tissue necrosis in Mali, DR Congo and South Africa. J Ethnopharmacol. 2014;157:171–80.

123. Ticli FK, Hage LIS, Cambraia RS, Pereira PS, Magro ÂJ, Fontes MRM, et al. Rosmarinic acid, a new snake venom phospholipase A2 inhibitor from Cordia verbenacea (Boraginaceae): antiserum action potentiation and molecular interaction. Toxicon. 2005;46(3):318–27.

124. Sanz L, Pla D, Pérez A, Rodríguez Y, Zavaleta A, Salas M, et al. Venomic analysis of the poorly studied desert coral snake, Micrurus tschudii tschudii, supports the 3FTx/PLA2 dichotomy across Micrurus venoms. Toxins (Basel). 2016;8(6):178.

125. Bolon I, Durso AM, Botero Mesa S, Ray N, Alcoba G, Chappuis F, et al. Identifying the snake: First scoping review on practices of communities and healthcare providers confronted with snakebite across the world. PLoS One. 2020;15(3):e0229989.

126. Ferraz CR, Arrahman A, Xie C, Casewell NR, Lewis RJ, Kool J, et al. Multifunctional toxins in snake venoms and therapeutic implications: from pain to hemorrhage and necrosis. Front Ecol Evol. 2019;7:218.

127. Oliveira ICF, de Paula MO, Lastra HCB, Alves B de B, Moreno DAN, Yoshida EH, et al. Activity of silver nanoparticles on prokaryotic cells and Bothrops jararacussu snake venom. Drug Chem Toxicol. 2019;42(1):60–4.

128. Tasoulis T, Isbister GK. A review and database of snake venom proteomes. Toxins (Basel). 2017;9(9):290.

129. Chippaux J-P. Snakebite envenomation turns again into a neglected tropical disease! J Venom Anim Toxins Incl Trop Dis. 2017;23:38.

130. Kang TS, Georgieva D, Genov N, Murakami MT, Sinha M, Kumar RP, et al. Enzymatic toxins from snake venom: structural characterization and mechanism of catalysis. FEBS J. 2011;278(23):4544–76.

131. Boldrini-França J, Cologna CT, Pucca MB, Bordon K de CF, Amorim FG, Anjolette FAP, et al. Minor snake venom proteins: Structure, function and potential applications. Biochim Biophys Acta (BBA)-General Subj. 2017;1861(4):824–38.

132. Laustsen AH, Lohse B, Lomonte B, Engmark M, Gutiérrez JM. Selecting key toxins for focused development of elapid snake antivenoms and inhibitors guided by a Toxicity Score. Toxicon. 2015;104:43–5.
