# Supplementary material for: A review and in silico screening of plant-derived snake venom/toxin inhibitors: ADMET, drug-likeness, and medicinal chemistry profiling
Source: PLoS Negl Trop Dis. 2025 Oct 9;19(10):e0013579. doi: 10.1371/journal.pntd.0013579 (PMC12527186; doi:10.1371/journal.pntd.0013579)
Supplement: Attachment — Submitted filename: Review comments.docx [file pntd.0013579.s003.docx]

Prince Ojuka et al., have systematically executed the job of compiling the plant compounds exhibiting snake venom/toxins inhibiting/neutralizing property. It is an elaborate study highlighting the drug-likeness of compounds such as coumarins, benzenoids, polyketides, and isoflavanoids for treating venomous snake bite. The manuscript is well written, however may be accepted with minor modifications

**Comments**

**f**= mention the author`s affiliation.

**Short title**: It is not appropriate, require modification suitably.

**Results and Discussion**

The analysis revealed 11distinct chemical classes (Figure 2), with Terpenoids (37), Flavonoids (32), Benzenoids (9), Alkaloids (17), Isoflavonoids (10), Hydroxycinnamic Acids (8), Saponins (10), Modified Glycosides (8), Polyketides (4), Tannins (5), and coumarins (3). Additionally, other identified compounds were 70.

**Comment:** In addition to Figure 2, assigning the plant compounds presented in Figure S1 under the above mentioned respective categories appear more meaningful and convincing.

**Conclusion**

(Lines 1127 and 1128) The results underscore the 1128 relevance of natural products as promising sources for antivenom agents and provide valuable leads for the development of new antivenom treatments.

Line 1133, antivenom agents

**Comment:** Though the word antivenom may be used, it is better to use a different word, may be therapeutic agents to avoid confusions.

The authors have to specify in their conclusion, whether to use compounds 23, 97, and 98 independently/together in what ratios, and further, compounds alone or as auxiliary agents along with antivenom to treat fatal snake bite.
