# Supplementary material for: A review and in silico screening of plant-derived snake venom/toxin inhibitors: ADMET, drug-likeness, and medicinal chemistry profiling
Source: PLoS Negl Trop Dis. 2025 Oct 9;19(10):e0013579. doi: 10.1371/journal.pntd.0013579 (PMC12527186; doi:10.1371/journal.pntd.0013579)
Supplement: Attachment — Submitted filename: Review Letter - Manuscript ID- PNTD-D-25-00699 .docx [file pntd.0013579.s004.docx]

Manuscript Title: A Review and In Silico Screening of Plant-Derived Snake Venom/Toxin Inhibitors: ADMET, Drug-Likeness, and Medicinal Chemistry Profiling
Manuscript ID: PNTD-D-25-00699

**Reviewer Comments:**

Dear Editors,

The manuscript addresses an important subject in snakebite envenomation. The review manuscript aims to identify and evaluate plant-derived compounds with inhibitory effects on snake venom toxins, using systematic review and in silico ADMET profiling tools.

While the manuscript presents valuable data and applies modern computational tools, I recommend **major revisions** before the manuscript can be considered for publication.

**Strengths:**

- The topic is timely and directly relevant to the PLOS NTD audience.
- The authors have compiled a large dataset (213 phytocompounds) and assessed them using comprehensive computational models.
- The discussion on ADMET and drug-likeness brings translational value to the findings.

**Key Concerns and Recommendations:**

1. **Manuscript Type Misclassification**: The paper should be reclassified as a "Systematic Review," not an original research article.
2. **Mechanistic organization**: Toxins and their inhibitors should be discussed based on molecular targets (e.g., PLA2, SVMPs, neurotoxins) to enhance clarity and mechanistic insight. Also, the underlying mechanisms, pharmacokinetics, and pharmacodynamics were not discussed and these are highlighted by authors in the justification as the major gaps in previous reviews
3. **Lack of regional diversity**: Authors presented plethora of compounds from medicinal plants against snake venoms mainly from Asia and South America continents. However, there are several researches from other continents on phytocompounds against snake venom toxins that meet the criteria set by the authors. It will make a more balanced and globally representative review if studies from other regions are also included to reflect regional diversity.
4. ***In silico* overinterpretation**: The assertion that some compounds are ready for drug development is premature and should be rephrased. Authors should emphasize the predictive not confirmatory nature of the computational analysis.
5. **References**: Reference formatting is inconsistent. Please standardize references and conform with the journal format.

**Conclusion:** This study has the potential to make a significant contribution to venom research and natural antivenom discovery. However, substantial revisions are necessary to enhance the manuscript's scientific rigor, global balance, and structural clarity. I recommend a **major revision**.

Sincerely,

Adeyi Akindele
