# Supplementary material for: A review and in silico screening of plant-derived snake venom/toxin inhibitors: ADMET, drug-likeness, and medicinal chemistry profiling
Source: PLoS Negl Trop Dis. 2025 Oct 9;19(10):e0013579. doi: 10.1371/journal.pntd.0013579 (PMC12527186; doi:10.1371/journal.pntd.0013579)
Supplement: Attachment — Submitted filename: PLOS Neglected Tropical Diseases - Reviewed.docx [file pntd.0013579.s005.docx]

PLOS Neglected Tropical Diseases

**A Review and In Silico Screening of Plant-Derived Snake Venom/Toxin Inhibitors: ADMET, Drug-Likeness, and Medicinal Chemistry Profiling**

--Manuscript Draft--

| **Manuscript Number:** | PNTD-D-25-00699 |
| --- | --- |
| **Full Title:** | A Review and In Silico Screening of Plant-Derived Snake Venom/Toxin Inhibitors: ADMET, Drug-Likeness, and Medicinal Chemistry Profiling |
| **Short Title:** | Phytocompounds with snakebite envenomation potential |
| **Article Type:** | Research Article |
| **Keywords:** | Snakebite envenomations; venom; Phytochemicals; toxins; ADMET |
| **Abstract:** | Snakebite envenomation remains a pressing global health issue, contributing to high rates of illness and death. It also imposes significant socio-economic burdens on affected communities. Recent conservative estimates suggest that approximately 5.4 million snakebite incidents occur annually, leading to nearly 138,000 fatalities. In Africa alone, as many as 500,000 cases are documented each year. This research seeks to explore phytochemicals reported to exhibit in vitro and in vivo inhibitory effects on snake venom and toxin targets. A systematic review was conducted utilizing seven electronic databases for literature searches, including Google Scholar, Web of Science, ScienceDirect, Scopus, Springer, and PubMed. This process identified 213 phytocompounds with inhibitory activity against the specified targets. Computational tools such as SwissADME, pkCSM, ADMETlab, ProTox3, Toxtree, and DataWarrior were employed to assess the absorption, distribution, metabolism, excretion, and toxicity (ADMET) characteristics, alongside other medicinal chemistry properties of these compounds. The results indicate that several plant-derived molecules effectively inhibit snake venom/toxin targets in vitro and in vivo; however, only a few appear suitable for drug development without further modifications. Among the analyzed compounds, the terpenes labdane lactone and labdane trialdehyde, along with the benzenoid anisic acid, exhibited strong antivenom potential. Notably, anisic acid achieved 100% neutralization of lethality and defibrinogenation caused by N. kaouthia,  D. russelii, O. hannah, and E. carinatus venoms in both in vivo and in vitro studies. Labdane lactone and labdane trialdehyde, isolated from C. antinaia and C. zedoaroides, respectively, exhibited significant venom inhibition at a concentration of 10 µg/mL. Specifically, labdane lactone demonstrated an 83% inhibition rate, while labdane trialdehyde showed a 62% inhibition rate against O. hannah venom, along with favorable in silico drug-likeness and ADMET profiles. |
| **Additional Information:** |  |
| **Question** | **Response** |
| **Government Employee**  Are you or any of the contributing authors an employee of the United States government?  Manuscripts authored by one or more US Government employees are not copyrighted, but are licensed under a [CC0](https://creativecommons.org/about/cc0) [Public Domain Dedication](https://creativecommons.org/about/cc0), which allows unlimited distribution and reuse of the article for any lawful purpose. This is a legal requirement for US Government employees. | No - No authors are employees of the U.S. government. |

| This will be typeset if the manuscript is accepted for publication. |  |
| --- | --- |
| **Financial Disclosure**  Enter a financial disclosure statement that describes the sources of funding for the work included in this submission. Review the [submission guidelines](http://journals.plos.org/plosntds/s/submission-guidelines#loc-financial-disclosure-statement) for detailed requirements. View published research articles from [*PLOS Neglected Tropical*](https://journals.plos.org/plosntds/) [*Diseases*](https://journals.plos.org/plosntds/) for specific examples.  This statement is required for submission and **will appear in the published article** if the submission is accepted. Please make sure it is accurate. | The author(s) received no specific funding for this work. |
| **Funded studies**  Enter a statement with the following details:   - Initials of the authors who received each award - Grant numbers awarded to each author - The full name of each funder - URL of each funder website - Did the sponsors or funders play any role in the study design, data collection and analysis, decision to publish, or preparation of the manuscript? |  |
| Did you receive funding for this work? |  |
| **Competing Interests**  On behalf of all authors, disclose any competing interests that could be perceived to bias this work.  This statement will be typeset if the manuscript is accepted for publication.  Review the instructions link below and PLOS NTDs’ [competing interests](https://journals.plos.org/plosntds/s/competing-interests#loc-what-represents-a-competing-interest) policy to determine what information must be disclosed at submission. | The authors affirm that there are no conflicts of interest. |

| **Data Availability**  Provide a **Data Availability Statement** in the box below. This statement should detail where the data used in this submission can be accessed. This statement will be typeset if the manuscript is accepted for publication.  Before publication, authors are required to make all data underlying their findings fully available, without restriction. Review our [PLOS Data Policy](https://journals.plos.org/plosntds/s/data-availability) page for detailed information on this policy. Instructions for writing your Data Availability statement can be accessed via the Instructions link below. | All relevant data are within the manuscript and its Supporting Information files. |
| --- | --- |

Manuscript	[Click here to access/download;Manuscript;Draft Manuscript-](https://www2.cloud.editorialmanager.com/pntd/download.aspx?id=1433970&guid=059e3b71-b79e-4f2f-995b-663e4e74b612&scheme=1)[<https://www2.cloud.editorialmanager.com/pntd/download.aspx?id=1433970&guid=059e3b71-b79e-4f2f-995b-663e4e74b612&scheme=1> nmk-240425 .docx](https://www2.cloud.editorialmanager.com/pntd/download.aspx?id=1433970&guid=059e3b71-b79e-4f2f-995b-663e4e74b612&scheme=1)

## 1 A Review and *In Silico* Screening of Plant-Derived Snake Venom/Toxin Inhibitors:

2 **ADMET, Drug-Likeness, and Medicinal Chemistry Profiling**
3 Prince Ojuka^a^, George S Nyamato^a^, Lucy Ochola^b^, George Omondi^b^, Cleydson B.R. Santos^d,e^, Njogu M. Kimani^a,c,*^
4 ^a^Department of Physical Sciences, University of Embu, Embu Kenya
5 ^b^Kenya Institute of Primate Research, Nairobi Kenya
6 ^c^Natural Product Chemistry and Computational Drug Discovery Laboratory, Embu, Kenya
7 ^e^ Laboratory of Modeling and Computational Chemistry, Department of Biological Sciences and Health, Federal
8 University of Amapá, Macapá, Amapá, Brazil
9 ^f^ Graduate Program in Medicinal Chemistry and Molecular Modelling, Health Science Institute, Federal University
10 of Pará, Belém 66075-110, PA, Brazil
11 *Correspondence Address: [njogu.mark@embuni.ac.ke](mailto:njogu.mark@embuni.ac.ke)

## 12 Abstract

13 Snakebite envenomation remains a pressing global health issue, contributing to high rates of illness
14 and death. It also imposes significant socio-economic burdens on affected communities. Recent
15 conservative estimates suggest that approximately 5.4 million snakebite incidents occur annually,
16 leading to nearly 138,000 fatalities. In Africa alone, as many as 500,000 cases are documented
17 each year. This research seeks to explore phytochemicals reported to exhibit *in vitro* and *in vivo*
18 inhibitory effects on snake venom and toxin targets. A systematic review was conducted utilizing
19 seven electronic databases for literature searches, including Google Scholar, Web of Science,
20 ScienceDirect, Scopus, Springer, and PubMed. This process identified 213 phytocompounds with
21 inhibitory activity against the specified targets. Computational tools such as SwissADME,
22 pkCSM, ADMETlab, ProTox3, Toxtree, and DataWarrior were employed to assess the absorption,
23 distribution, metabolism, excretion, and toxicity (ADMET) characteristics, alongside other
24 medicinal chemistry properties of these compounds. The results indicate that several plant-derived
25 molecules effectively inhibit snake venom/toxin targets *in vitro* and *in vivo*; however, only a few
26 appear suitable for drug development without further modifications. Among the analyzed
27 compounds, the terpenes labdane lactone and labdane trialdehyde, along with the benzenoid anisic
28 acid, exhibited strong antivenom potential. Notably, anisic acid achieved 100% neutralization of
29 lethality and defibrinogenation caused by *N. kaouthia*, *D. russelii*, *O. hannah*, and *E. carinatus*
30 venoms in both *in vivo* and *in vitro* studies. Labdane lactone and labdane trialdehyde, isolated from
31 *C. antinaia* and *C. zedoaroides*, respectively, exhibited significant venom inhibition at a
32 concentration of 10 µg/mL. Specifically, labdane lactone demonstrated an 83% inhibition rate,
33 while labdane trialdehyde showed a 62% inhibition rate against *O. hannah* venom, along with
34 favorable *in silico* drug-likeness and ADMET profiles.
35 **Keywords:** Snakebite envenomations, venom, Phytochemicals, toxins, ADMET

36

## 37 GRAPHICAL ABSTRACT

38

39

## 40 1. Introduction

41 Snakebite envenomations (SBEs) are a significant but often neglected public health concern,
42 affecting populations globally, particularly in tropical and subtropical developing regions.(1,2)
43 High cases in these areas are partly attributed to the preservation of forests and biodiversity.
44 However, human expansion and urbanization into these biodiverse regions have led to increased
45 human-snake interactions, particularly in Latin America, Sub-Saharan Africa, and Asia.(2–4)
46 Annually, an estimated over five million snakebites occur worldwide, resulting in approximately
47 138,000 deaths and leaving around 400,000 individuals with lasting physical disabilities.(5,6) Asia
48 reports the highest number of cases, accounting for 73% of global incidents (two million), with
49 India alone recording over forty six thousand deaths in 2020.(1) Africa and the Middle East follow,
50 with about five hundred and eighty thousand cases (21%), including seven thousand to thirty two
51 thousand deaths in Sub-Saharan Africa.(1,7) Latin America and the Caribbean report
52 approximately one hundred and fifty thousand cases (5%) and five thousand deaths, with most
53 incidents occurring in South America. Brazil encounters twenty six thousand to twenty nine
54 thousand cases annually, roughly one-third of which take place in the Amazon region.(6,8)
55 These estimates likely underrepresent the true number of SBE cases, as many incidents go
56 unreported.(4) Underreporting is common because SBEs often occur in remote rural areas where
57 access to healthcare services is limited.(4,9) Despite significant advancements in healthcare,
58 managing snakebites remains a challenge. While antivenom therapy can reduce mortality, it does
59 not address local tissue damage (10). Furthermore, the availability of antivenom is scarce in many
60 remote regions.(2) Due to these treatment challenges, some communities have developed
61 traditional methods to mitigate snakebite effects.(8,9) Traditional healers worldwide use medicinal
62 plants for treating snakebites, and it is common in these regions to use extracts or teas made from
63 leaves, roots, or bark.(1,4,8,9) However, the unscientific and unsafe application of such remedies
64 often leads to adverse clinical outcomes, delaying or complicating effective treatment by
65 healthcare professionals.(1)
66 Currently, numerous medicinal plants are being assessed through *in vivo* and *in vitro* studies to
67 validate their therapeutic benefits. Previous phytochemical analyses of bioactive plant extracts
68 have led to the identification of several bioactive compounds, including terpenoids, alkaloids,
69 steroids, flavonoids, and quinones. (11) Furthermore, secondary metabolites from plants that were
70 not previously recognized for their pharmacological potential are now being explored as sources
71 of new medicinal agents. These bioactive compounds serve as valuable lead molecules in the
72 process of discovering and developing new drugs.(12) Various computational approaches have
73 been applied across different stages of discovering and developing new drugs. In recent years, *in*
74 *silico* ADMET modeling has gained considerable recognition as a valuable tool in rational drug
75 design. (13–15) The effectiveness and affordability of these models have enhanced the drug
76 development process by enabling the prediction of compound bioavailability, aiding in hit
77 identification, and optimizing molecular structures.(16) Recently, there has been a notable rise in
78 research articles examining the *in silico* evaluation of plant-derived compounds. Additionally,
79 computational screening of natural product databases has expanded rapidly.(17) However,
80 systematic reviews specifically addressing the molecular targets of plant metabolites with anti-
81 snake venom properties have been scarce. Although some recent reviews have examined plant-
82 based antivenom activities, most lack detailed discussions on the underlying mechanisms,
83 pharmacokinetics, and pharmacodynamics.(1) Another study reviewed plant-based inhibitors but
84 focused solely on bothropic venom.(18) Unlike such compilations, this study offers a
85 comprehensive and critical evaluation of bioactive plant-derived compounds for their therapeutic
86 potential against snakebite envenomation. Additionally, it explores new possibilities for their
87 application in the development of novel snakebite treatments through ADMET and medicinal
88 chemistry profiling of plant compounds as snake toxin inhibitors.

89

## 90 2. Methods

91 **2.1 Study Design**
92 This systematic review was conducted in accordance with the PRISMA (Preferred Reporting Items
93 for Systematic Reviews and Meta-Analyses) guidelines (19). The research question was structured
94 using the PICO framework (Problem, Intervention, Control, Outcome), as recommended by
95 PRISMA: "In *in vitro* and *in vivo* studies (I) using venom-related target models, what is the
96 potential of plant-derived compounds (P) in neutralizing venom activity (O) compared to control
97 groups and standard antivenom treatments (C)?"

## 98 2.2 Search strategy

99 An extensive review of the literature from various scientific sources, including Google Scholar,
100 ScienceDirect, Web of Science, Scopus, Springer, and PubMed was conducted. The study
101 databases comprised original research articles from peer-reviewed journals, books, dissertations,
102 theses, and patents. Scientific data written in or translated into English and published up to
103 December 2024 were included in the review. The search involved using keywords such as
104 “snakebites,” “snake envenomation,” “snake venom,” “natural inhibitors,” “antivenom activity,”
105 “toxins,”	“plants,”	“phospholipase	inhibitors,”	and	“metalloprotease	inhibitors,”	either
106 individually or in combination. Studies presenting bioactivity data of plant-derived compounds
107 tested in both *in vitro* and *in vivo* models against snake venoms, their specific toxins, or the
108 biological effects induced by those toxins were considered relevant and included.

## 109 2.3 Inclusion Criteria

110 Studies reporting data on the efficacy of plant-derived compounds in neutralizing snake venom
111 through *in vitro* and *in vivo* experiments were considered for inclusion.

## 112 2.4 Exclusion Criteria

113 Studies focusing solely on compounds from non-plant sources were excluded. Additionally,
114 studies evaluating the activity of crude extracts, fractions, or compound mixtures were not
115 considered.

## 116 2.5 Data Collection and Extraction

117 To organize the materials for this systematic review, we compiled all relevant articles. Duplicates
118 were removed, and a new file containing all the relevant material and its consolidated data was
119 generated. Data extraction included various factors: the name of each plant-derived compound, its
120 chemical structure (which was drawn using ChemDraw v.12), the chemical class of the compound,
121 the target venom models investigated, and the specific *in vitro* or *in vivo* assays employed.
122 Additionally, information on the concentrations of plant compounds tested, numerical results of
123 inhibitory activity, and units of measurement for both concentration and assay results was
124 recorded. We also noted the types of snakes used in the studies and any observed suppression of
125 venom activity. This extracted information underwent a thorough verification process to ensure
126 accuracy and completeness. Any inconsistencies were addressed through discussion and resolved
127 by consensus, with arbitration used when necessary.

## 128 2.6 Risk of Bias

129 This study identified several potential sources of bias, such as the criteria for inclusion and
130 exclusion, the selection of databases, language barriers, and variations among the studies. The
131 quality of the studies was evaluated based on their publication in peer-reviewed journals. To
132 minimize bias, protocols such as PICO and PRISMA were rigorously adhered to. The biases
133 considered included factors like the inclusion/exclusion criteria, missing data, database selection,
134 language differences, publication dates, and the effect of missing primary data. Additionally, the
135 types of articles included in the review were assessed to maintain methodological rigor.

## 136 2.7 Methodology Summary

137 We calculated all numerical data collected during the review process. This involved determining
138 the number of articles retrieved from each database using specific search terms, the total number
139 of articles in the compiled file, and those removed due to duplication. We also documented the
140 number of articles that were either included or excluded based on independent evaluations, as
141 depicted in **Figure 1**.

142

**Identification of articles via databases**

143

144

Records removed *before screening*:

Duplicate records removed (n

= 1740)

Records removed for other reasons (n = 320)

Records identified from Databases (n =3201)

**Identification**

145

146

147

148

149

150

151

152

153

Records excluded (n =350)

Records screened (Title, abstract, keywords)

(n =1141)

154

155

156

Articles considered valid (n = 134)

Full-text screened (n = 791)

**Screening**

157

158

Reports excluded (n =657)

159

160

161

162

163

**Included**

164

165

166

167

168 **Figure 1.** PRISMA flow chart of the screening process.
169 **2.8 *In Silico* Analysis**
170 To assess ADMET properties, drug-likeness, and other medicinal chemistry parameters, a
171 combination	of	computational	tools	was	utilized,	including	SwissADME
172 (<http://www.swissadme.ch/>),	pkCSM	(<http://biosig.unimelb.edu.au/pkcsm/>),	ADMETlab
173 (<http://admet.scbdd.com/>),	ProTox3	(<https://tox-new.charite.de/protox_III/>),	Toxtree
174 (<https://toxtree.sourceforge.net/>), and DataWarrior (<http://www.openmolecules.org/datawarrior/>).
175 Molecular structures were generated, validated, and saved in SMILES format using ChemDraw v.
176 12 (<https://revvitysignals.com/chemdraw>) before being submitted to the selected software and
177 platforms for analysis. Each parameter, including drug-likeness (DL), absorption (A), distribution
178 (D), metabolism (M), excretion (E), toxicity (T), and medicinal chemistry (MC) was evaluated
179 using at least two different platforms. The obtained data were cross-verified, and only the
180 parameters with matching results were considered in the screening process to enhance the accuracy
181 and reliability of predictions. A sequential filtering approach was applied, progressively reducing
182 the number of compounds based on predefined cutoff criteria. (20–22) This refinement process
183 enabled the selection of molecules with the highest pharmacokinetic and pharmacological
184 potential.

## 185 3. Results and Discussion

186 After extracting data from the reviewed and validated studies, a total of 213 plant-derived
187 compounds were identified as inhibitors of snake toxins and venom. The analysis revealed 11
188 distinct chemical classes (**Figure 2**), with Terpenoids (37), Flavonoids (32), Benzenoids (9),
189 Alkaloids (17), Isoflavonoids (10), Hydroxycinnamic Acids (8), Saponins (10), Modified
190 Glycosides (8), Polyketides (4), Tannins (5), and coumarins (3). Additionally, other identified
191 compounds were 70

**80**

**Number of Compounds**

**60**

**40**

**20**

**0**

**Other Compounds**

**Terpenoids**

**Flavonoids**

**Alkaloids**

**Isoflavonoids**

**Saponins**

**Benzenoids**

**Modified Glycosides**

**Tannins**

**Polyketides**

**coumarins**

**Classes of Compounds**

**Hydroxycinnamic Acids**

192

193 **Figure 2.** Classes of compounds, and the number of compounds tested against targets of snake
194 venom and toxins

## 195 3.1 Venom Toxins

196 Over 250,000 species utilize venoms for various purposes, including capturing prey, deterring
197 rivals, or defending against predators. Their evolutionary success is demonstrated by the presence
198 of venomous organisms in every ecosystem.(23) In animals like snakes, envenomation involves
199 delivering venom into another organism's tissue through specialized fangs. This venom, a toxin-
200 rich secretion, aids in immobilizing and digesting prey, although it also serves as a defense
201 mechanism.(1,24) Genetic mutations and natural selection have driven the diversification of the
202 venom proteome across snake families, resulting in species-specific toxicities.(25) Snake venoms
203 mainly consist of proteins and peptides, making up 90–95% of their composition, which may or
204 may not exhibit enzymatic activity. Key constituents include metalloproteases (SVMPs),
205 phospholipases A2 (PLA2s), serine proteases (SVSPs), L-amino acid oxidases (LAAOs),
206 hyaluronidases (HAases), phosphodiesterases (PDEs), acetylcholinesterases (AchEs), nucleases,
207 disintegrins, three-finger toxins (3-FTxs), C-type lectins (CTLs), and cysteine-rich secretory
208 proteins (22). Not all venoms contain every toxin type, as synthesis and secretion vary across
209 production cycles, resulting in compositional differences.(26,27)
210 Despite containing over 20 protein families, most snake venom effects stem from four primary
211 families: PLA2s, SVMPs, SVSPs, and 3-FTxs. These proteins target multiple physiological
212 systems, causing various pathological effects.(1,28) Elapid venoms, rich in neurotoxic PLA2s and
213 3-FTxs, primarily induce neurotoxicity, which can escalate to respiratory failure, a potentially fatal
214 condition. They typically cause minimal local effects.(6) Conversely, viper venoms are rich in
215 proteases, leading to local and systemic effects, including pain, swelling, blistering, bleeding, and
216 ecchymosis. Severe cases may result in complications such as bacterial infections, compartment
217 syndrome, tissue necrosis, and amputations. Systemic effects like acute kidney injury are also
218 commonly observed.(6,29) The composition and concentration of venom toxins can vary based on
219 the snake’s age, sex, diet, and geographic location, influencing the severity and type of
220 envenomation outcomes.(30,31)

## 221 3.2 Plant Compounds as Antivenom Agents

222 Natural compound-based binding inhibitors offer a promising and safe treatment approach for
223 snakebite envenomation. As a result, these plant-derived therapies could serve as effective
224 alternatives, especially in cases where specific antivenom treatments are unavailable (1). This
225 review discusses the latest research findings on compounds with anti-snake venom properties and
226 provides a summary of their specific molecular targets (**Supplementary File; Table S1 and**

## 227 Figure S1).

228 **3.2.1 Alkaloids**
229 Alkaloids exhibit a wide range of biological activities, yet only a limited number of studies have
230 reported their inhibitory effects on snake venom enzymes (**Supplementary File; Table S1 and**
231 **Figure S1**). One such compound, Schumanniofoside (**1**), a glycosylated benzopyranylpiperidinone
232 alkaloid derived from the stem bark of *Schumanniophyton magnificum* (Rubiaceae) in Nigeria,
233 demonstrated the ability to reduce the lethal effects of *Naja melanoleuca* venom *in vivo* at varying
234 doses (10–100 mg/kg), with an 80 mg/kg dosage decreasing mouse mortality by 15%. (32)
235 A different study emphasized the *in vivo* potential of 12-methoxy-4-methylvoachalotine (**2**), an
236 indolic	alkaloid	featuring	an	iboga-type	skeleton.	Extracted	from	the	root	bark	of
237 *Tabernaemontana catharinensis* (Apocynaceae) in São Paulo, Brazil, this compound fully
238 counteracted the lethal effects of *Crotalus durissus* venom when administered at a dose of 1.7
239 mg/100g, 20 seconds after injecting mice with two times the mean lethal doses (LD50). (33)
240 Furthermore, two steroidal alkaloids, 22α,23α-epoxy-solanida-1,4,9-trien-3-one (**3**) and 22α,23α-
241 epoxy-solanida-1,4-dien-3-one (**4**), extracted from *Solanum campaniforme* leaves (Solanaceae) in
242 Ceará, Brazil, were evaluated for their activity against *Bothrops pauloensis* venom.
243 Their anti-myotoxic, anti-hemorrhagic, and anti-necrotizing properties were evaluated following
244 *in vitro* venom incubation. The presence of these alkaloids significantly reduced necrotic areas
245 (~27.0 and 32.0 mm², respectively) and minimized hemorrhagic damage.(34) Further investigation
246 into the same plant species led to the identification of four steroidal alkaloids exhibiting biological
247 activity against *B. pauloensis* venom, including: (E)-N-[8′(4-hydroxyphenyl)ethyl]-22α,23α-
248 epoxy-solanida-1,4,9-trien-3-imine (**8**), 22β,23β-epoxy-solanida-4-en-3-one (**7**), 22α,23α-epoxy-
249 solanida-4-en-3-one (**6**), (Z)-N-[8′(4-hydroxyphenyl)ethyl]-22α,23α-epoxy-solanida-1,4-dien-3-
250 imine (**9**), and 22β,23β-epoxy-solanida-1,4-dien-3-one (**5**). Compounds **3**, **5**, **6**, and **9** demonstrated
251 anti-hemorrhagic activity, whereas compounds **6**, **7**, **8**, and **9** exhibited anti-necrotic properties.(35)
252 Berberine (**10**), derived from *Cardiospermum halicacabum* (Sapindaceae) in India, was identified
253 as a phospholipase A2 (PLA2) inhibitor in *Daboia russelii* venom. The compound’s inhibitory
254 activity was confirmed through co-crystallization experiments, which revealed its potential as an
255 anti-inflammatory agent for snakebite treatment. (36)
256 Another potential antivenom agent is aristolochic acid (**11**), a nitrophenanthrene carboxylic acid
257 found in *Aristolochia* species (Aristolochiaceae) from India. It demonstrated *in vitro* inhibitory
258 effects against L-amino acid oxidase (LAAO) in *D. russelii* venom (19% inhibition, IC50 = 33.6
259 µM) (37) and suppressed the venom-induced edema by 50% at a concentration of 0.16 µM (146).
260 Additionally, pre-incubation with this compound (13.7 µg/mL) significantly decreased the
261 myotoxic effects of piratoxin-I (PrTX-I) from *Bothrops pirajai* venom. (38) However, aristolochic
262 acid is known to interact with DNA, leading to carcinogenic effects, rendering it unsuitable for
263 therapeutic use. (37)
264 To overcome this limitation, semi-synthetic derivatives of aristolochic acid were developed by
265 substituting the nitro group with either a chlorine atom (**12**) or a hydroxyl group (**13**). These
266 modifications eliminated DNA binding while preserving LAAO inhibitory activity in *D. russelii*
267 venom. Furthermore, compound **12** demonstrated superior inhibition of LAAO-induced reactive
268 oxygen species (ROS) production in human embryonic kidney (HEK293) cells (76%) and human
269 hepatocellular carcinoma (HepG2) cells (68%), while compound **12** also significantly reduced
270 ROS levels. (37) 12-Methoxy-Nb-methyl-voachalotine (**13**), extracted from *Tabernaemontana*
271 *catharinensis* (Apocynaceae), exhibited strong inhibitory effects against the lethality and myotoxic
272 activities induced by *Crotalus durissus terrificus* venom. This compound effectively neutralized
273 crotoxin, the primary toxin in the venom (39). Shenoy et al. (2013) identified piperine (**14**) as a
274 constituent of the ethanolic fruit extract of *Piper longum L.* Piperine was found to interact with
275 phospholipase A2 from *Daboia russelii* venom.(40) Atropine (**15**), a well-known tropane alkaloid,
276 was found to exhibit potential antivenom activity. Its pharmacological effects, including
277 modulation of acetylcholine receptors and inhibition of venom-induced neuromuscular blockade,
278 contribute to its protective role in envenomation scenarios. These properties suggest that atropine
279 could be a valuable adjunct in snakebite treatment strategies.(41) Ajmaline **(16)**, known for its
280 pharmacological activities, has demonstrated partial inhibition of *Naja naja* venom hyaluronidase
281 (HAase) and NNH1 activity. Although not a complete inhibitor, its observed effects suggest that it
282 may be beneficial in reducing venom spread through enzymatic neutralization.(42) Similarly,
283 anisodamine **(17)** from *Anisodus tanguticus* (*Zang Qie*) relieved venom-induced microcirculatory
284 issues and associated renal failure.(39)

## 285 3.2.2 Benzenoids

286 Several simple compounds containing substituted benzene nuclei have been identified in certain
287 plants and may serve as potential inhibitors of snake venom (**Supplementary File; Table S1 and**
288 **Figure S1**). One such plant, *Hemidesmus indicus* (Apocynaceae), native to India, has been the
289 subject of studies investigating the venom-neutralizing potential of 2-hydroxy-4-methoxybenzoic
290 acid (**18**). This compound demonstrated inhibitory effects against the venoms of *Daboia russelii*,
291 *Naja kaouthia*, *Ophiophagus hannah*, and *Echis carinatus*. (43–45) It effectively counteracted the
292 venom’s toxic effects, reducing lethality, hemorrhagic activity, and coagulation disturbances, while
293 also mitigating the inflammation induced by *D. russelii* venom in rodents.
294 An additional compound derived from the same plant, 2-hydroxy-4-methoxybenzaldehyde (**19**),
295 exhibited PLA2 inhibition (*in vitro*) and significantly reduced both lethality and hemorrhagic
296 activity caused by *D. russelii* venom.(46) This compound was also extracted in substantial amounts
297 from *Janakia arayalpatra* (Periplocaceae) in Jammu, India. This led researchers to synthesize
298 semi-synthetic derivatives (**20-22**), which displayed venom-neutralizing activity against *D.*
299 *russelii*. In *in vivo* experiments, these derivatives effectively counteracted hemorrhagic effects,
300 lethality, and PLA2-induced toxicity. Additionally, all of the synthesized compounds neutralized
301 the hemorrhagic and lethal effects of *N. kaouthia* venom. (47)
302 Further studies revealed the venom-inhibitory properties of anisic acid (**23**) and salicylic acid (**24**),
303 also derived from *H. indicus*. These compounds were found to counteract the venoms of *D.*
304 *russelii*, *E. carinatus*, *N. kaouthia*, and *O. hannah*. Compound **23** completely neutralized the lethal
305 effects and defibrinogenation induced by the venom in both *in vitro* and *in vivo* studies, while
306 compound **24** entirely inhibited venom-induced hemorrhagic activity.(43) However, the precise
307 mechanisms through which these compounds exert their venom-neutralizing effects remain
308 unclear. (43,48)
309 Gallic acid (**25**) was found to suppress the proteolytic activity of *D. russelii* venom in vitro (IC50

310	= 0.58 µM), though it did not suppress the PLA2 activity of the same venom. (49) Nevertheless, a

311 study by Pereañez et al (2011) demonstrated that gallic acid inhibited PLA2 enzymatic activity by
312 63% and reduced PLA2-induced cytotoxicity by approximately 78% in *Crotalus durissus* venom.
313 (50) Additionally, this compound, which was extracted from *Anacardium humile* (Anacardiaceae)
314 in Minas Gerais, Brazil, exhibited strong myotoxic inhibition against the venom of *Bothrops*
315 *jararacussu* and its principal myotoxins, bothropstoxin I and II (BthTX-I and BthTX-II).
316 Furthermore, gallic acid significantly reduced hemorrhagic activity (IC50 = 0.2 µM) and oedema
317 formation (IC50 = 2 µM) in in vivo experiments. (50)
318 Vanillic acid (**26**), on the other hand, displayed selective and specific inhibition of the enzymatic
319 activity of 5'-nucleotidase (5’AMP), a venom enzyme known to affect hemostasis by preventing
320 platelet aggregation. Found in the venom of *Naja naja*, this enzyme contributes to anticoagulant
321 effects. In a dose-dependent manner, vanillic acid reduced the anticoagulant effect of *N. naja*
322 venom by up to 40%. Research indicates that compound **26** interacts with one or more intrinsic
323 blood clotting factors, disrupting the anticoagulant properties of the venom.(51)

## 324 3.2.3 Hydroxycinnamic Acids

325 Rosmarinic acid (**27**), an esterified hydroxycinnamic acid found in various plants, is well known
326 for its antivenom properties. When extracted from *Cordia verbenacea* (Boraginaceae), this
327 compound was shown to inhibit both oedema and myotoxic activity caused by the basic PLA2s,
328 BthTX-I and BthTX-II. It was particularly effective in almost completely neutralizing the
329 myotoxic effects and partially reducing edema triggered by these enzymes, suggesting a
330 dissociation between their catalytic and pharmacological domains. (52) Additionally, rosmarinic
331 acid has been effective against the venoms of *Agkistrodon bilineatus, Deinagkistrodon acutus*,
332 *Crotalus atrox, Gloydius blomhoffii*, *Bitis arietans*, and *Trimeresurus flavoviridis* (53–55)

## 333 (Supplementary File; Table S1 and Figure S1).

334 Caffeic acid (**28**) has also demonstrated venom-neutralizing potential. When complexed with
335 piratoxin-I, a PLA2 containing a lysine residue at position 49 (PLA2s-Lys49) in *Bothrops pirajai*
336 venom, it led to partial inhibition of PrTX-I's myotoxic activity (38). Furthermore, this compound
337 suppressed 41% of the cytotoxic activity induced by PLA2s from *Crotalus durissus*. (50)
338 A modified derivative, triacontyl *p*-coumarate (**29**), isolated from *Bombacopsis glabra*
339 (Bombacaceae) in Bahia, Brazil, showed promising effects against the toxicity of *Bothrops*
340 *pauloensis* venom, as well as isolated SVMPs (jararhagin) and PLA2s (BnSp-6). (56) Compound
341 **29** significantly reduced fibrinogenolytic activity and plasma fibrinogen depletion (53%) induced
342 by the venom or its isolated toxins. It also effectively inhibited hemorrhagic activity (3 MDH) and
343 hemorrhagic effects caused by jararhagin (56) (**Supplementary File; Table S1 and Figure S1**).
344 The compound *p*-coumaric acid (**30**) formed a complex with PLA2s from *Daboia russelii* and
345 demonstrated strong catalytic inhibition, with an IC50 value of 38.0 µM.(57) Chlorogenic acid (**31**)
346 and cynarin (**32**) were also tested for their ability to inhibit venom-induced mortality in mice, with
347 chlorogenic acid showing a high inhibition rate (90%) against *Bothrops jararaca* venom, while
348 cynarin displayed a weaker effect (30%). (58)
349 Ferulic acid (**33**) exhibited significant PLA2 inhibitory activity and effectively reduced oedema
350 formation caused by PLA2s from *Crotalus durissus*. (59) Another study reported that this
351 compound inhibited PLA2 enzymatic activity by 17% and reduced PLA2-induced cytotoxicity by
352 37% in *C. durissus* venom. (50) In the same study, propyl gallate (**34**) was also shown to inhibit
353 the enzymatic (51%) and cytotoxic (94%) activity of PLA2s from this species (**Supplementary**

## 354 File; Table S1 and Figure S1).

355 **3.2.4 Tannins**
356 Several research studies have highlighted the role of some tannins as bioactive compounds with potential antivenom properties. Gallotannin tannic acid (**35**), which occurs naturally in various plants but is also
357 commercially available, effectively inhibited hyaluronidases (HAases) and hemorrhagic effects
358 while reducing the lethal impact of *Crotalus adamanteus* venom *in vivo*. This inhibition
359 significantly increased the survival time of test mice. (60)
360 Ellagitannins, extracted from the leaves of *Casearia sylvestris* (Salicaceae) in São Paulo, Brazil,
361 were tested for their venom-neutralizing properties. Two compounds, ellagic acid (**36**) and 3’-O-
362 methyl ellagic acid (**37**), were evaluated for their effects on venom and PLA2s (Asp 49 BthTX-II)
363 from *Bothrops jararacussu*. The inhibition constant (Ki) values for enzymatic activity were
364 approximately 3 nM for 27 and 7 nM for 28. Additionally, their IC50 values for reducing
365 oedematogenic and myotoxic activities were 23.8 µM and 34 µM, respectively (61)

## 366 (Supplementary File; Table S1 and Figure S1).

367 Casuarictin (**38**), another ellagitannin, was sourced from the leaves of Laguncularia racemosa
368 (Combretaceae) in São Paulo, Brazil. This compound formed a stable protein complex with PLA2s
369 isolated from *Crotalus durissus* venom. Molecular interactions between casuarictin and PLA2s
370 effectively neutralized the native oedematogenic effects and prevented toxin-induced
371 myonecrosis when administered 10 minutes after PLA2s injection. As a result, casuarictin shows
372 promise as a potential anti-inflammatory agent for treating PLA2s-induced oedema and
373 myonecrosis (62). Another study investigated the ethanolic extract of *Mangifera indica*
374 (Anacardiaceae) seed grains, particularly its primary active component, 1,2,3,4,6-pentagalloyl
375 glucopyranose (**39**). This compound exhibited inhibitory effects on the enzymatic activities of
376 PLA2s, HAases, and L-amino acid oxidase (LAAO) from *Calloselasma rhodostoma* and *Naja naja*
377 venoms in *in vitro* assays (63) (**Supplementary File; Table S1 and Figure S1**).

## 378 3.2.5 Coumarins

379 The incubation of BnIV, a Lys49 PLA2 from *Bothrops neuwiedi* venom, with coumarin
380 umbelliferone (**40**), a compound abundant in *Citrus* species, nearly prevented platelet clumping,
381 oedema (IC50 0.2 µM), and myotoxicity caused by BnIV while also reducing its inflammatory
382 effects. Compound **40** interacted with Asp and Lys residues in the catalytic site of PLA2s, which
383 are crucial for the toxin’s activity. (64)
384 Among coumarins, the dihydrofuranocoumarin (+)-alternamin (**41**), a newly identified compound
385 isolated from the aerial components of *Murraya alternans* (Rutaceae) in Myanmar, inhibited
386 venom-induced bleeding by *Trimeresurus flavoviridis* by 24% at a concentration of 250 µg/mL
387 compared to the control. (65) Additionally, bergapten (**42**), derived from *Dorstenia brasiliensis*
388 (Moraceae) in Brazil (Rio de Janeiro state), showed a modest effect in reducing the lethality of
389 *Bothrops jararaca* venom in mice, decreasing lethality by 20% (58) (**Supplementary File; Table**

## 390 S1 and Figure S1).

391 **3.2.6 Flavonoids**
392 Flavonoids exhibit a wide range of biological activities, with anti-inflammatory and antioxidant
393 properties being particularly notable. These characteristics make several flavonoids promising
394 candidates as antivenom agents. In this context, two thrombin-like serine proteases (SP1 and SP2)
395 from *Crotalus simus* venom were isolated and incubated *in vitro* with the flavanone aglycone
396 hesperetin (**43**), commonly found in *Rutaceae* species. The results showed that hesperetin acts as
397 a potent non-competitive inhibitor against SP1 and a mixed inhibitor against SP2. This naturally
398 occurring flavone, which can be easily extracted from oranges, presents a cost-effective option for
399 inhibiting the studied snake venom proteases. (66) Additionally, hesperetin **43** inhibits
400 phospholipase A2 (PLA2) activity in *Crotalus atrox* venom. (64) Furthermore, this compound,
401 obtained from orange peels, functioned as a reversible inhibitor of serine proteases isolated from
402 *Bothrops jararaca* venom (67).
403 The flavanone pinostrobin (**44**), extracted from the leaves of *Renealmia alpinia* (Zingiberaceae) in
404 Colombia, a plant traditionally used in folk medicine for snakebite treatment, was evaluated for its
405 effectiveness against the venoms of *Crotalus durissus* and *Bothrops asper*. Pinostrobin exhibited
406 an IC50 of 1.76 µM against PLA2 activity in *C. durissus* venom. In experiments where mice were
407 injected with PLA2s and treated with pinostrobin at concentrations of 0.4, 2.0, and 4.0 µM,
408 myotoxic activity was inhibited by up to 87% (68). This compound also demonstrated the ability
409 to inhibit the proteolytic effects (22%) induced by *B. asper* venom and reduced hemolytic activity
410 by 21%. The same study found that *R. alpinia* extract inhibited the indirect hemolytic, coagulant,
411 and proteolytic activities of *B. asper* venom following in vitro pre-incubation.
412 The glycosylated flavanone hesperidin (**45**), isolated from *Citrus sinensis* (*Rutaceae*), exhibited
413 moderate inhibitory activity against the lethality induced by *B. jararaca* venom. (58) Synthetic
414 flavones, including the aglycones apigenin (**46**) and luteolin (**47**), inhibited the hyaluronidase and
415 hemorrhagic actions of *Crotalus adamanteus* venom and reduced its lethality.(60,69) Additionally,
416 synthetic apigenin (**46**) was found to inhibit PLA2 activity in *C. atrox* venom.(70) The flavones
417 pectolinarigenin (**48**) and hispidulin (**49**), extracted from *Aegiphila integrifolia* (Verbenaceae) in
418 Brazil (Roraima state), partially inhibited PLA2 activity in *Bothrops atrox* venom by 20% and 15%,
419 respectively, while also reducing hyaluronidase activity by 60% and 40%, respectively (70).
420 Another flavone, morelloflavone (**50**), a dimeric flavone isolated from *Garcinia*
421 *madruno* (Clusiaceae) in Colombia, exhibited IC50 values of 0.48 µM and 0.38 µM in PLA2
422 enzymatic activity for aggregate and monodisperse substrates, respectively.
423 Flavonols have been extensively studied due to their frequent occurrence and accessibility through
424 phytochemical approaches. The aglycone quercetin (**51**), isolated from *Morus nigra* (Moraceae)
425 (117),	*Phyllanthus	klotzschianus*	(Phyllanthaceae)	(58),	and	*Erythroxylum	ovalifolium*
426 (Erythroxylaceae) (71), demonstrated potent inhibition of oedema, proteolytic activity, and
427 lethality caused by the venoms of *Bothrops jararacussu*, *Lachesis muta*, and *B. jararaca*,
428 respectively. Synthetic quercetin (**52**) also inhibited PLA2 activity in *Daboia russelii* venom (IC50
429 2 µM) and *Naja naja* venom, achieving a maximum inhibition of 40%.(72) Additionally, research
430 by Cotrim et. Al., (2011) highlighted quercetin’s potential to inhibit PLA2 activity in *D. russelii*
431 venom and its ability to prevent platelet aggregation and myotoxic effects induced by *C. durissus*
432 venom by approximately 40%.(73) Quercetin also completely inhibited purified hyaluronidase
433 activity in *N. naja* venom (74,75).
434 Kaempferol (**53**) has demonstrated antivenom potential (60), as have the synthetic flavonoids
435 fisetin (**54**) and myricetin (**55**), which exhibited inhibitory effects against PLA2 activity in *C. atrox*
436 venom. Additionally, myricetin **55** effectively inhibited the proteolytic and hemorrhagic activities
437 of *B. atrox* venom, with IC50 values of 150 µM and 1 µM, respectively. (76) The same study
438 identified quercitrin (**56**), a synthetic glycosylated flavonol, as an effective inhibitor of *C. atrox*
439 venom PLA2s. Furthermore, quercitrin **56**, extracted from the leaves of *Brownea rosa-de-monte*
440 (Fabaceae) in Panama, significantly inhibited the coagulant and hemorrhagic effects of *B. asper*
441 venom. At a concentration of 0.1 µM, quercitrin extended plasma coagulation time by two to six
442 times.(77)
443 Another glycosylated flavonol, quercetin-3-O-rhamnoside (**57**), isolated from *Euphorbia hirta*
444 (Euphorbiaceae) in India, showed strong inhibition (93%) of *N. naja* PLA2 activity in vitro using
445 egg yolk as a substrate and also reduced hyaluronidase and hemolytic activity.(78) Furthermore,
446 quercetin-3-O-rhamnoside reduced edema and lethality, extending the survival of mice.(78) Rutin
447 (**58**) exhibited a 28% inhibition of *L. muta* venom-induced hemorrhagic activity *in vivo* (71) and
448 demonstrated a 40% inhibitory effect against PLA2 activity in *C. atrox* venom (72).
449 Taxifolin (**59**), a synthetic flavanonol, showed potential for inhibiting PLA2 enzymatic activity in
450 *C. atrox* venom (72). Several studies have also identified flavan-3-ols as potential antivenom
451 agents. Catechin (**60**), extracted from the stem of *Scolopia chinensis* (Salicaceae) in China,
452 demonstrated inhibitory activity (16%) against snake venom phosphodiesterase I (PDE I). (79)
453 This compound also showed PLA2 inhibition in *C. atrox* venom and has been synthesized.(72)
454 The flavan-3-ol gallocatechin (**61**), obtained from the leaf extract of *Schizolobium parahyba*
455 (Fabaceae) in Brazil (Minas Gerais state), neutralized the biological and enzymatic effects of
456 venoms and toxins from *B. jararacussu* and *B. neuwiedi*.(80) Gallocatechin exhibited significant
457 inhibition of hemorrhagic and fibrinogenolytic activity of isolated SVMPs (Bjussu-MP-I, Bjussu-
458 MP-II) and also reduced myotoxic effects induced by *Bothrops alternatus* venom and BnSP-6 (a
459 Lys49 PLA2 from *B. neuwiedi*). (80)
460 Epigallocatechin gallate (**62**), a major compound in *Ilex paraguariensis* (yerba mate), reduced the
461 cytotoxic effects of myotoxic PLA2s from *C. durissus* venom, achieving an IC50 of 0.38 µM,
462 indicating its potential as an antimyotoxic agent. (50) The chalcone butein (**63**), isolated from
463 *Butea monosperma* (Fabaceae), inhibited the activity of daboxin P, a PLA2, with an IC50 of 541
464 µM. Moreover, it completely inhibited PLA2 activity in *N. naja* venom (100%) and reduced
465 activity in *Bungarus caeruleus* (49%), *D. russelii* (72%), and *Echis carinatus* (47%) at 1,200 µM
466 (81) Primetin (**64**), a flavonoid isolated from *Primula denticulata* (Primulaceae), was found to
467 inhibit toxins from snake venom (39). Vale et al. (2011) investigated the protective effects of
468 Myricetin-3-O-Glucoside (**65**) from the leaf extract of *Schizolobium parahyba* (*Leguminosae*)
469 against snake venoms.(80) The flavonoids hypolaetin-8-glucoside (**66**) was reported to have
470 phospholipase A2 activities. Hypolaetin-8-glucoside was isolated from *Sideritis mugronensis*
471 (Lamiaceae).(82) Isoquercitrin (**67**), a flavonoid extracted from the root of *Solanum incanum*,
472 demonstrated inhibitory effects on acetylcholine responses. Additionally, it significantly reduced
473 intestinal motility in mice, suggesting a potential neuromodulatory role in venom-induced
474 physiological	disturbances.(83)	A	structurally	complex	flavonoid,	patuletin-3-O-α-L-
475 rhamnopyranosyl-7-O-α-L-rhamnopyranoside (**68**), was identified in *Bryophyllum pinnatum*
476 leaves and exhibited notable antivenom activity. It demonstrated potential by modulating venom-
477 induced oxidative stress and enzymatic activity.(84) Iridin (**69**), a flavonoid, demonstrates the
478 ability to bind to biological polymers, inhibiting PLA2 activity. Flavonoids like iridin, along with
479 other members of the class such as quercetin and luteolin, show potential as anti-snake venom
480 agents due to their structural features that allow for effective interaction with venom
481 components.(85) Quercetin-3-O-sophoroside (**70**), isolated from ethanolic extracts of *Cissampelos*
482 *pareira* aerial parts, demonstrated protective effects against proteolytic activity of *Bothrops*
483 *diporus* venom. (10) Quercetin derivatives are known to inhibit lipoxygenase, and although
484 specific PLA2 inhibition was not confirmed for this glycoside, its structural features suggest
485 potential to modulate venom-induced inflammation. Nishijima et al. (2009) identified
486 amenthoflavone **(71)** as active principles in *Byrsonima crassa* (Malpighiaceae) against *N. naja*
487 venom. Myricetin (**72)** was potent against the hemorrhagic effects of *Bothrops jararaca*. (86)
488 Isoquercitrin **(73)** from *Schizolobium parahyba* (leaves) demonstrated anti-hemorrhagic and anti-
489 fibrinogenolytic activities against *Bothrops* venoms, including *B. jararacussu* and *B. neuwiedi*, at
490 concentrations of 100–1250 μM. (87) Patuletin-3-O-Glycoside **(74)**, isolated from the leaves of
491 *Bryophyllum pinnatum*, was characterized as a bioactive compound with antivenom properties.
492 Using HPLC-DAD-MS, researchers confirmed its presence and potential role in counteracting
493 venom-induced toxicity. Its mechanism of action is likely related to flavonoid-mediated enzyme
494 inhibition and antioxidant effects, contributing to venom neutralization.(84) (**Supplementary**

## 495 File; Table S1 and Figure S1).

496 **3.2.7 Isoflavonoids**
497 The isoflavone harpalycin 2 (**75**), extracted from the leaves of *Harpalyce brasiliana* (Fabaceae) in
498 Ceará, Brazil, has been traditionally used in folk medicine as an anti-inflammatory remedy for
499 snakebites. Research on its effects revealed promising biological activity. Compound **75**
500 demonstrated inhibitory effects on the enzymatic activity, oedema formation, and myotoxic effects
501 of phospholipase A2s (PLA2s) from *Bothrops pirajai*, *Crotalus durissus*, and *Naja naja* venoms.
502 Specifically, harpalycin 2 (Har2) inhibited Piratoxin 3 (PrTX-III) from *B. pirajai* by 59%, *C.*
503 *durissus* venom by 79%, and *N. naja* venom by 88%. Additionally, oedema induced in mouse paws
504 by PLA2s was significantly reduced in the early phase upon administration of Har2. The compound
505 also mitigated the myotoxic effects of these PLA2s. (88,89)
506 Another isoflavone, 7,8,3’-trihydroxy-4’-methoxyisoflavone (**76**), isolated from *Dipteryx alata*
507 (Fabaceae) in Tocantins, Brazil, demonstrated the ability to counteract neurotoxicity and
508 myotoxicity in *B. jararacussu* venom, as observed in mouse phrenic nerve-diaphragm
509 experiments. (90) When venom was pre-incubated with 55 (200 µg/mL), it reduced neuromuscular
510 blockade by 84%. It also attenuated the effects of BthTX-I, the primary myotoxic PLA2s in this
511 venom. Histological evaluation of the diaphragm muscle treated with **76** showed that 91% of
512 muscle fibers remained intact, compared to 50% damage in venom-exposed samples.(90)
513 Beyond isoflavonoids, derivatives such as pterocarpans and coumestans have also demonstrated
514 antivenom properties. Edunol (**77**), a pterocarpan isolated from the roots of *Brongniartia*
515 *podalyrioides* (Fabaceae) in Mexico, decreased the lethality of *B. atrox* venom by 30% in mice
516 that had received an LD50 dose of venom and were subsequently treated with 3.1 mg/kg of the
517 compound (91). This substance was also found in the roots of *Harpalyce brasiliana* in Brazil and
518 was later synthesized to yield greater quantities for further testing. Synthetic edunol exhibited
519 significant antimyotoxic, antiproteolytic, and anti-PLA2s properties. (92) The potency of these
520 effects was further enhanced by modifying edunol into its bioisostere derivative (**78**), where the
521 prenyl group was replaced by a benzyl group. Pre-incubation of venom with **78** *in vitro* resulted in
522 complete inhibition of myotoxic activity, with an IC50 of 9.97 µM. At a concentration of 100 mM,
523 this pterocarpan also inhibited 65% of the phospholipase activity of *B. jararacussu* venom and
524 over 80% of its proteolytic activity. (92)
525 Additional prenylated pterocarpans, particularly those with modifications in ring A, have shown
526 strong activity against *B. atrox* venom. Notably, cabenegrins A-I (**79**) and A-II (**80**), isolated from
527 *Annona crassiflora* (Annonaceae), a medicinal plant widely used in northeastern Brazil for treating
528 snakebites, emerged as promising lead compounds.(93) The coumestan wedelolactone (**81**),
529 identified in *Eclipta alba* and *E. prostrata* (Asteraceae) from São Paulo, Brazil, has also
530 demonstrated antivenom activity.(94–96) A study by Cedro et al. (2018) found that it effectively
531 inhibited the myotoxic activity of PLA2s from *C. durissus* and *B. jararacussu* (BthTX-I and
532 II).(97) Additionally, compound **81** significantly reduced the proteolytic, PLA2s, and myotoxic
533 activities of *B. jararacussu* venom, with an IC50 of 1 µM.(98)
534 An analogue of wedelolactone (**82**), synthesized with modified oxygenation patterns in rings A
535 and D, exhibited activity at 30 µM. This compound effectively antagonized the release of creatine
536 kinase (CK) induced by *B. jararacussu* venom in skeletal muscle. Its inhibitory effect on venom-
537 induced myotoxicity (IC50 = 1 µM) was comparable to that of wedelolactone. (98) Moreover, **82**
538 showed a lower affinity for benzodiazepine receptors, suggesting a reduced likelihood of central
539 nervous system side effects.(98) These findings indicate that such compounds could be valuable
540 in snakebite therapy and the treatment of coagulation disorders.(98) Furthermore, another study
541 confirmed the inhibitory potential of demethylwedelolactone (**83**), also derived from *E. alba*, in
542 neutralizing myotoxicity induced by isolated PLA2s (BthTX-I and II) from *C. durissus* and *B.*
543 *jararacussu* venom.(96) Pterocarpan (**84**), exhibited promising antivenom potential. Its
544 pharmacological effects included modulation of inflammatory responses, inhibition of venom
545 enzymes, and neutralization of oxidative stress induced by envenomation. These findings highlight
546 its significance in the search for plant-based antivenom agents.(41) (**Supplementary File; Table**

## 547 S1 and Figure S1).

548 **3.2.8 Modified Glycosides**
549 The	compound	2-(6-benzoyl-β-glucopyranosyloxy)-7-(1α,2α,6α-trihydroxy-5-oxocyclohex-3-
550 enoyl)-5-hydroxybenzyl alcohol **(85)** was isolated from the bark and branches of *Bennettiodendron*
551 *leprosipes* and *Flacourtia ramontchi* (Salicaceae), both traditionally used in folk medicine. This
552 compound exhibited 14% inhibition against PDE-I. (99) In the same study, homaloside D **(86)**
553 displayed a similar effect, inhibiting PDE-I by 13%. Additionally, itoside B **(87)** and itoside F **(88)**,
554 extracted from *Itoa orientalis* (Salicaceae), demonstrated inhibition levels of 21% and 13%,
555 respectively, against PDE-I (99).
556 Although these modified glycosides exhibited activity, their potency was lower compared to other
557 natural product classes. Two new phenolic glycosides, scoloquinenoside C **(89)** and scoloposide C
558 **(90)** were isolated from the stem of *Scolopia chinensis* (Salicaceae) in China. Phenolic glycosides,
559 in general, have demonstrated inhibitory effects against PDE-I derived from snake venom. (100)
560 Another study also confirmed PDE-I inhibition from snake venom, where two newly identified
561 phenolic glycosides, benzoylsalreposide **(91)** and salireposide **(92)**, were evaluated. Both
562 compounds, isolated from *Symplocos racemosa* (Symplocaceae) in Pakistan, exhibited an IC50
563 value of 171 µM (98,101) (**Supplementary File;Table S1 and Figure S1**).

## 564 3.2.9 Polyketides

565 The natural naphthoquinone lapachol **(93)** was extracted from *Tabebuia impetiginosa*
566 (Bignoniaceae) in Brazil (state of Rio de Janeiro) and has served as a precursor for developing new
567 bioactive quinones. (102) In the same study, an analogue of this compound, **93**, exhibited the
568 ability to counteract the proteolytic activity (3–100 µM) and collagenase activity (10–100 µM) of
569 *Bothrops atrox* venom. Furthermore, *in vivo* pre-incubation of the venom with **93** at doses of 1
570 mg/kg and 3 mg/kg prevented hemorrhage caused by *B. atrox* venom. Against *B. jararaca* venom,
571 the inhibition exceeded 70% when treated with 10 mg/kg of the compound. The authors suggested
572 that **93**’s protective effect on the skin results from its ability to inhibit proteolytic and collagenase
573 activity, making it a potential candidate for preventing vascular degradation. (102)
574 Another naphthoquinone, isohemigossypolone **(94)**, was isolated from the roots of *Pachira*
575 *aquatica* (Malvaceae) in Brazil (state of Bahia). This compound significantly reduced the *in vitro*
576 coagulant activity of *Bothrops pauloensis* venom. *In vivo* tests further demonstrated its ability to
577 inhibit the venom-induced myotoxic effects and neutralize 70% of the metalloproteinase activity
578 of whole venom, while reducing the activity of the isolated BthMP enzyme by 40% (103).
579 Selvanayagam et al. identified a quinonoid xanthene from the root bark of *Ehretia buxifolia*
580 (Boraginaceae) in India (Tamil Nadu), a plant traditionally used as an antidote for *Echis carinatus*
581 envenomation. (104) The isolated compound, ehretianone **(95)**, was obtained from the methanolic
582 extract of the bark and tested for antivenom properties against *E. carinatus* venom in mice. In a
583 prophylactic treatment, administering 3.75 mg/kg of the compound 30 minutes before venom
584 injection reduced mortality by 35% compared to untreated controls. In a curative study, the same
585 dosage provided significant protection when given within 5 minutes after venom injection.
586 Melanin **(96)**, extracted from black tea, is a non-hydrolyzed complex of tea polyphenols that has
587 been investigated for its effects on the venoms of *Agkistrodon contortrix*, *Agkistrodon halys*, and
588 *Crotalus atrox*. *In vitro* assays showed that it reduced the specific activity of the PLA2s enzyme by
589 43%, while in vivo experiments demonstrated a notable increase in the survival time of mice
590 following administration of venom from these three snake species. (105)(**Supplementary File;**

## 591 Table S1 and Figure S1)

592 **3.2.10 Terpenoids**
593 Salama and colleagues isolated, labdane lactone **(97**), and labdane trialdehyde **(98**), from various
594 *Curcuma* species (family Zingiberaceae) in Thailand. Compounds **97** and **98**, obtained from *C.*
595 *antinaia* and *C. zedoaroides*, demonstrated venom inhibition rates of 83% and 62%, respectively,
596 at a concentration of 10 µg/mL against *Ophiophagus hannah* venom. Notably, compound **98**
597 preserved diaphragmatic contraction almost entirely, offering 99% protection. This makes it the
598 most promising candidate for antivenom and anti-neurotoxic therapy.(106)
599 The sesquiterpene α-turmerone (**99**), extracted from *C. longa* (Zingiberaceae) in Brazil, neutralized
600 hemorrhagic activity in *Bothrops jararaca* venom and reduced *Crotalus durissus* venom lethality
601 by 70% in rats. (107) It also decreased hemorrhage and oedema from *B. alternatus* venom,
602 reversing necrosis in 96 hours.(108) The diterpene (E)-17-ethyliden-labd-12-ene-15,16-dial (**100**),
603 from *C. zedoaroides* (Zingiberaceae) in Thailand, fully neutralized *Ophiophagus hannah* venom
604 *in vitro* at 32 µg/mL and increased survival by >80% in mice (109). It guided dialdehyde to the
605 venom's peptide target, preventing neurotoxic effects. Further studies on diterpenes **101–103** (from
606 *Curcuma* species) showed significant antivenom activity, with compound **103** offering the highest
607 protection (99%).
608 The neo-clerodane (**104**) from *Baccharis trimera* (Asteraceae) in Brazil inhibited hemorrhagic and
609 proteolytic activities in *Bothrops* venoms, with over 80% inhibition of SVMPs.(109) Among
610 triterpenes, lupeol acetate (**105**) from *Hemidesmus indicus* neutralized lethal, hemorrhagic, and
611 neurotoxic effects of *Daboia russelii* and *Naja kaouthia* venoms. (110) Compounds **106–107** from
612 *Clematis gouriana* (Ranunculaceae) in India effectively inhibited PLA2s. Oleanolic acid (**108**)
613 from medicinal plants inhibited PLA2s in *D. russelii* and *N. naja* venoms (>90%), reducing venom-
614 induced oedema and hemorrhagic effects. (106)
615 Betulinic (**109**) and ursolic acids (**110**) inhibited proteolytic and hemorrhagic activities in *Bothrops*
616 *atrox* venom, showing strong antivenom properties. (76) Quinovic acid (**111**) (from *Mitragyna*
617 *stipulosa* (Rubiaceae) inhibited venom PDE-I.(111) Arjunolic acid (**112**) from *Combretum*
618 *leprosum* (Combretaceae) in Brazil reduced lethality (>80%) and inhibited multiple enzymatic
619 activities in *Bothrops* venoms.(112)
620 Friedelin (**113**) and lupeol (**114**) from *Erythroxylum* species in Brazil showed modest antivenom
621 activity.(71) Triterpenoids from *D. alata* (Fabaceae) countered *B. jararacussu* neuromuscular
622 blockade, with compounds **115–117** offering 45–70% protection. β-amyrin (**118**) from *Apuleia*
623 *leiocarpa* (Fabaceae) improved survival against *B. jararaca* venom.(94)
624 Among steroids, ikshusterol 3-O-glucoside (**119**) from *C. gouriana* neutralized *N. naja* venom
625 moderately. β-sitosterol (**120**) and stigmasterol (**121**) from *Pluchea indica* protected against *D.*
626 *russelii* and *N. kaouthia* venom effects. The synthetic corticosteroid corticosterone (**122**) inhibited
627 PLA2s in *D. russelii* venom. Bakuchiol (**123**), a synthetic meroterpenoid, inhibited the main PLA2
628 enzyme *daboxin P.*(57,81,113) Bt-CD (7α-hydroxy-3,13-clerodadiene-16,15:18,19 diolide) (**124**),
629 a clerodane diterpenoid isolated from *Baccharis trimera*, displayed anti-proteolytic and anti-
630 hemorrhagic properties against Bothrops venom. It effectively neutralized hemorrhagic and
631 fibrinogenolytic activities of P-I and P-III metalloproteinases from *Bothrops neuwiedi* and
632 *Bothrops jararacussu* venoms (39)*.* The furanoid diterpene lactone **(125)** from *Aristolochia albida*
633 (rhizome) provided *in vivo* protection from venom-induced mortality caused by *Naja nigricollis*
634 and *Bitis arietans*, with an effective dosage range of 5–80 mg/kg.(87) 2-Hexenal **(126),** 2-Hexen-
635 1-ol **(127),** Elixene **(128) ,** (−)-Globulol **(129) ,** and Thujopsene **(130)** isolated from *Bidens pilosa*
636 L. leaves and whole plant extracts exhibited significant inhibitory activity against venoms from
637 *Dendroaspis jamesoni* and *Echis ocellatus*.(83)
638 d-α-pinene **(131)** and camphene **(132)**, isolated from *Citrus limon* root and ripe fruit methanolic
639 extracts, exhibited *in vitro* neutralization of *N. naja karachiensis* venom-induced coagulant effects,
640 achieving 64% inhibition(10). Furthermore, an ED₅₀ of 710 µg/mouse was reported against the
641 lethal effects of *Lachesis muta* venom. These compounds may disrupt enzymatic venom
642 components or enhance host defense mechanisms via membrane stabilization. d-Limonene **(133)**,
643 isolated from the methanolic extracts of *Citrus limon* L. Burm. f. (roots and ripe fruits),
644 demonstrated promising antivenom activity. It effectively neutralized the coagulant effect of weak
645 phospholipase A₂ (PLA₂) enzymes found in *Naja naja karachiensis* venom, achieving 64%
646 inhibition with a coagulation time of 109 ± 1.00 seconds. Furthermore, it exhibited *in vitro*
647 inhibitory effects against the lethal action of *Lachesis muta* venom, with an ED₅₀ of 710 µg of
648 extract per mouse, indicating its potential utility as a broad-spectrum antivenom compound(10).

## 649 (Supplementary File; Table S1 and Figure S1)

650 **3.2.11 Saponins**
651 Certain saponins have also been identified as inhibitors of snake venom. The triterpene saponin
652 bredemeyeroside B (**134**), derived from the roots of *Bredemeyera floribunda* (Polygalaceae) found
653 in the Brazilian state of Ceará, demonstrated inhibitory effects against the lethal action of *Bothrops*
654 *jararaca* venom when administered orally at a dose of 100 mg/kg. This resulted in a 90% survival
655 rate among the tested animals.(114) Another compound from the same plant species,
656 bredemeyeroside D (**135**), also exhibited venom lethality inhibition, with a 100% survival rate of
657 mice after six hours under the same experimental conditions as the previous study.(115)
658 Glycyrrhizin (**136**), obtained from the roots of *Glycyrrhiza glabra* (Fabaceae) in São Paulo, Brazil,
659 demonstrated *in vitro* inhibition of human fibrinogen coagulation triggered by *B. jararaca* venom,
660 with an IC50 of 1.2 mM. Additionally, it showed hydrolytic activity *in vitro* (IC50 0.47 mM) and
661 inhibitory effects on platelet aggregation (IC50 0.33 mM) (**137**). In *in vivo* tests, compound 98
662 significantly reduced thrombus weight by 86% against venom-induced effects and successfully
663 prevented venom-related bleeding when co-administered with antivenom (114).
664 Macrolobin A (**138**) and macrolobin B (**139**), isolated from the bark of *Pentaclethra macroloba*
665 (Fabaceae) in Amapá, Brazil, effectively inhibited the hemorrhagic and fibrogenolytic activities of
666 *Bothrops* venoms and the SVMP Bjussu-MP-I from *B. jararacussu* venom in a dose-dependent
667 manner.(116) Compound **139** exhibited the most promising results, with inhibition of up to 90%
668 of the raw venom’s proteolytic activity and 80% of fibrin SVMPs, particularly classes I and III. In
669 terms of coagulation activity, compound **139** completely inhibited the effects of *B. jararacussu*
670 venom and the thrombin-like enzyme Bjussu-SP-I after incubation for one hour and 30 minutes,
671 respectively.(116)
672 Glycosidic derivatives of quinovic acid, initially isolated from the bark of *Bridelia ndellensis*
673 (Euphorbiaceae)	in	Ngaoundéré,	Cameroon,	displayed	inhibitory	activity	against
674 phosphodiesterase-I (PDE-I). The compounds quinovic acid-3-O-α-L-rhamnopyranoside (**140**),
675 quinovic acid-3-O-β-D-fucopyranoside (**141**), and quinovic acid-3-O-β-D-glucopyranosyl (1 →
676 4)-β-D-fucopyranoside (**142**) significantly inhibited the enzyme, with IC50 values of 85 µM, 85
677 µM, and 75 µM, respectively (117) A triterpenoid saponin, the potassium salt of gymnemic acid
678 **(143)**, was isolated from *Gymnema sylvestre*. This compound inhibited ATPase activity induced
679 by *Naja naja* venom.(118) (**Supplementary File; Table S1 and Figure S1**).

## 680 3.2.12 Other Compounds

681 Fozing et al. (2012) demonstrated that certain compounds extracted from the leaves of *Morus*
682 *mesozygia* (Moraceae) inhibit the phosphodiesterase I enzyme of commercial origin. Among them,
683 mesozygin B (**144**) and artonine I (**145**) exhibited the strongest activity, with IC50 values of 8.9
684 µM and 15.4 µM, respectively.(119) Other compounds have also been shown to effectively inhibit
685 enzymes found in snake venom. The stilbene resveratrol (**146**) and the aliphatic derivative
686 polyamine gramine {3-[3-(dimethylaminomethyl)-1H-indol-7-YL] propan-1-ol} (**147**) were
687 tested for their ability to inhibit the PLA2s enzyme from *Daboia russelii* venom, with IC50 values
688 of 43.4 µM and 50.4 µM, respectively. (57)
689 In *in vitro* assays, the amino acid mimosine [β-3-(3-hydroxy-4-oxopyridyl) α-amino-propionic
690 acid] (**148**) inhibited HAases (DRHyal-II) in a dose-dependent manner, achieving complete
691 inhibition at 24 µM and an IC50 of 12 µM. Additionally, compound **148** neutralized the
692 hyaluronidase activity of *D. russelii* venom, which was entirely suppressed at 1000 µM, with an
693 IC50 of 500 µM. In *in vivo* experiments, **148** also reduced the myotoxic effects of the myotoxin
694 VRV-PL-VIII (a myotoxic PLA2s), which was potentiated by DRHyal-II.(120) According to a
695 study by Devi et al. (2020), this synthetic compound inhibited the PLA2s enzyme activity of raw
696 venom (5 µg/ml) from *Naja naja* (47.0%), *Echis carinatus* (47.0%), and *D. russelii* (27%) at a
697 concentration of 1,200 µM. Furthermore, compound **148** inhibited the PLA2s activity of daboxin
698 P with an IC50 of 674.3 µM, indicating its anticoagulant potential.(81)
699 Another study confirmed the medicinal value of diisobutyl phthalate, 2-methylpropyl phthalate
700 (**149**), isolated from the root of *Emblica officinalis* (Phyllanthaceae) in India, which counteracted
701 the myotoxicity induced by *D. russelii* venom. This was evidenced by a reduction in the levels of
702 myotoxicity marker enzymes CPK and LDH.(121) Similarly, the bioactive polyphenol curcumin
703 (**150**) exhibited strong inhibition of hyaluronidase (HAases) activity purified from *N. naja* venom,
704 showing a 91% reduction.(75)
705 Additionally, plant extracts commonly used in traditional medicine were tested for their ability to
706 inhibit enzymatic activity in myotoxin I and PLA2s from *Bothrops asper* venom. The compound
707 4-nerolidylcatechol (**151**), obtained from *Piper umbellatum* and *P. peltatum* (Piperaceae) in Costa
708 Rica (Upala and Guápiles), inhibited the PLA2s activity of myotoxin I from *B. asper* and *B. atrox*
709 in a time- and dose-dependent manner (IC50 = 1 mM). This compound was also effective against
710 PLA2s activity in group I pseudexin and *Micrurus mipartitus* venom, as well as group II *Bothrops*
711 toxins.(122) Pre-incubation of **151** with myotoxins from venoms of these snake species reduced myotoxic
712 activity by approximately 50% and significantly decreased the inflammatory response in both
713 cases.(122) Additionally, in *in vivo* studies, pre-treatment with 0.4 mg of **151** up to one hour before
714 toxin administration resulted in a one-third reduction in venom-induced oedema. Research by
715 Nuñez et al. also demonstrated that this compound inhibited the proteolytic activity of trypsin on
716 casein by 75% and eliminated the procoagulant action of an SVSP enzyme isolated from *B.*
717 *jararacussu* venom in *in vitro* tests (122) (**Supplementary File; Table S1 and Figure S1)**.
718 The fatty alcohol 1-hydroxytetratriacontan-4-one (**152**), extracted from the leaves of *Leucas*
719 *aspera* (Lamiaceae), exhibited strong venom-neutralizing effects against *N. naja* in *in vivo* tests.
720 It had a median effective dose (ED50) of 34.47 mg and a fully effective dose (ED100) of 68.93 mg
721 per animal. A 100% survival rate was achieved when envenomed mice were treated simultaneously
722 with **152** at a dose of 75 mg per animal. Additionally, it significantly reduced venom-induced
723 oxidative stress and lipid peroxidase activity in multiple organs.(123) 12-Methoxy-4-
724 Methylvoachalotine (MMV) **(153)** a natural product from *Tabernaemontana catharinensis*, has
725 demonstrated inhibition of myotoxic PLA2 activity and reduction in the lethality of *Crotalus*
726 *durissus terrificus* venom. Its activity further supports the role of alkaloid-rich plant extracts in
727 mitigating venom-induced toxicities (42).
728 Silybin (I) **(154),** isolated from *Silybum marianum*, has been reported for its potential anti-venom
729 properties. However, further investigations are needed to establish its mechanism of action in
730 venom neutralization.(42) Phloretin **(155)**, classified as a dihydrochalcone, has been identified
731 with inhibitory activity against SVHYs (snake venom hyaluronidases).
732 *n*-Propyl Gallate **(156),** a known antioxidant, exhibited inhibitory potential against *Naja naja*
733 venom HAase.(42) Butylated Hydroxytoluene (BHT) **(157)**, a synthetic antioxidant, has been
734 studied for its potential to inhibit venom HAases. While it demonstrated some inhibitory activity,
735 its efficacy was lower than that of NDGA and curcumin, suggesting a limited role in snakebite
736 treatment.(42) Methyl gallate **(158)**, isolated from *Mangifera indica* seed kernel extract, exhibited
737 inhibitory effects against the proteolytic activity of *Calloselasma rhodostoma* and *Naja naja*
738 *kaouthia* venoms. The presence of pentagalloylglucopyranose (tannic acid) in this extract
739 significantly enhanced its activity, suggesting synergistic effects in venom neutralization.(39)
740 Raghavamma et al. (2016) reported the antivenom activiy of α-amyrin **(159)** from the leaf extract
741 of *Pergularia daemia* (Forsk.) Chiov. (124)
742 Sarkhel et al. (2011) isolated di-iso-butyl phthalate **(160)** from the root extract of *Emblica*
743 *officinalis* (*Phyllanthus emblica*), a species in the Euphorbiaceae family. This compound
744 demonstrated activities against viper and cobra venoms, neutralizing venom-induced lethality,
745 coagulant activity, defibrinogenation, fibrinolysis, free radical generation, hemorrhage, and
746 myotoxic effects.(121)
747 Lattmann et al. (2010) isolated curcuma dialdehyde **(161)** from *Curcuma zedoaria*. This C20
748 dialdehyde exhibited significant *in vitro* and *in vivo* antivenom activity against *Ophiophagus*
749 *hannah* (King cobra) venom. The compound antagonized the venom’s effects on neuromuscular
750 transmission in isolated rat phrenic nerve-hemidiaphragm preparations.(109) Short-chain
751 hydrocarbons 2,4-dimethylhexane (**162**), 2,6-dimethylheptane **(163)**, and 2-methylnonane (**164**)
752 were isolated from *Tragia involucrata*. These compounds demonstrated PLA2 neutralization
753 activities in mice, reducing venom-induced toxicity.(39) Dharmappa et al. (2010) extracted
754 genistein **(165)** from *Glycine max*. The compound exhibited strong inhibitory effects against *Naja*
755 *naja* phospholipase-I and RV-PL-V, significantly reducing venom-induced paw edema in
756 mice.(125) Mahadeswaraswamy et al. (2011) isolated mimosine, a β-3-(3-hydroxy-4-oxopyridyl)
757 α-amino propionic acid (**166**), from *Mimosa pudica*. The compound exhibited significant *in vitro*
758 inhibition of hyaluronidase activity induced by *Vipera russelli* venom.(126)
759 Leucasin **(167),** the methanolic extract of *Leucas aspera* (Lamiaceae) leaves exhibited activity
760 against venom protease and hyaluronidase activities. Studies identified leucasin in *L. aspera*
761 extract as the active component. Leucasin neutralized PLA2 type I enzyme from snake venom and
762 demonstrated a dose-dependent antioxidant activity.(127)	AIPLAI **(168)**, isolated from
763 *Azadirachta indica*, exhibited anti-PLA2 activity against *Naja kaouthia* and *Daboia russelii*.(128)
764 Catechin-3’-O-rhamnopyranoside **(169)**, found in the stem of *Neocarya macrophylla*, inhibited
765 PLA2 activity in *Naja nigricollis* venom at concentrations of 1–10 mg/kg.(129)
766 Quinonoid xanthene ehretianone **(170)**, isolated from *Ehretia buxifolia*, exhibited anti-snake
767 venom activity. The LD50 of the venom used was 6.65 mg/kg, and the compound did not show any
768 toxic effects up to 100 mg/kg.(87) An organic acid (designated HI-RVIF) (**171)**, isolated from the
769 root extract of *Hemidesmus indicus* (Indian sarsaparilla), displayed inhibitory activity against viper
770 venom-induced lethal, hemorrhagic, coagulant, and anticoagulant effects in rodents. Spectral
771 analysis confirmed the presence of a benzene ring, methoxy group, and hydroxyl group. Alpha-
772 tocopherol **(172)**, the most active form of vitamin E, inhibited PLA2 activity selectively toward
773 lamellar fluid membranes, thereby protecting phospholipids from enzymatic degradation. This
774 compound reduced both the initial rate and overall extent of hydrolysis. The inhibition was non-
775 competitive, suggesting that alpha-tocopherol mainly affected the substrate (membrane) rather
776 than the enzyme itself.(130)
777 Cardiac Glycosides **(173)** isolated from the leaves of *Costus afer* demonstrated significant *in vivo*
778 protection against snake venom-induced toxicity. This compound exhibited potential antivenom
779 properties, through their ability to inhibit venom enzymes and modulate physiological responses
780 to envenomation. Their efficacy suggests a promising avenue for developing plant-based
781 treatments for snakebite management.(131) D-Mannitol **(174)**, a naturally occurring sugar alcohol
782 extracted from *Mimosa pudica*, was found to possess significant antivenom properties. It
783 functioned by counteracting the toxic effects of venom components, potentially through
784 osmoregulatory mechanisms and interaction with venom enzymes. Its bioactivity suggests that it
785 may serve as a complementary agent in snakebite treatment strategies.(131)
786 Polyisoprenylated Benzophenones **(175)** isolated from the leaves, stems, flowers, and fruits of
787 *Clusia fluminensis* exhibited *in vivo* venom-neutralizing activity against *Bothrops jararaca* venom.
788 This compound demonstrated potential by inhibiting enzymatic pathways involved in venom
789 toxicity, thereby reducing the systemic damage caused by envenomation. Their pharmacological
790 potential underscores their significance in natural antivenom research.(131)
791 2-Hydroxy-4-Methoxy Benzoic Acid **(176)**, isolated from *Hemidesmus indicus*, exhibited strong
792 bioactive properties that contributed to its antivenom potential. This phenolic compound likely
793 exerts its protective effects through enzyme inhibition and antioxidant mechanisms, reducing
794 venom-induced tissue damage and systemic toxicity.(41)
795 D-X-Pinene Camphene **(177)** extracted from *Citrus* spp. (*C. limon L. Burm. F*) roots and ripe fruits
796 using methanol, this compound effectively neutralized the anticoagulant effect induced by weak
797 PLA2 enzymes from *Naja naja karachiensis* venom, achieving 64% inhibition with a coagulation
798 time of 109 ± 1.00 s. Additionally, it demonstrated *in vitro* inhibition of the lethal effects of
799 *Lachesis muta* venom, with an effective dose (ED50) of 710 μg extract per mouse.(83)
800 Ichangin 4-β-Glucopyranoside **(178)** derived from *Citrus* spp. (*C. limon L. Burm. F*) root and ripe
801 fruit extracts using methanol, this compound exhibited anticoagulant neutralization against weak
802 PLA2 enzymes in *Naja naja karachiensis* venom (64% inhibition, coagulation time of 109 ± 1.00
803 s). It also displayed inhibitory potential against *Lachesis muta* venom lethality, with an effective
804 dose of 710 μg extract per mouse.(83)
805 Nomilinic Acid **(179)** isolated from *Citrus* spp. (*C. limon L. Burm. F*) root and ripe fruit extracts
806 in methanol, nomilinic acid significantly neutralized anticoagulant effects caused by weak PLA2
807 enzymes in *Naja naja karachiensis* venom (64% inhibition, coagulation time of 109 ± 1.00 s) and
808 demonstrated *in vitro* inhibition of *Lachesis muta* venom lethality at an effective dose of 710 μg
809 extract per mouse.(83)
810 Lignan (-)-Cubebin **(180)** derived from *Aristolochia* spp. (*A. indica*, *A. odoratissima*) leaf extracts
811 using methanol, ethanol, pentane, and water, (-)-cubebin inhibited PLA2 and hyaluronidase
812 enzymes from *Naja naja* and *Vipera russelli* venoms. It also exhibited strong gelatinolytic,
813 collagenase, peroxidase, and nuclease inhibitory activities and protected mice from lethal effects
814 of *Bothrops atrox* venom at higher doses of 8 and 16 mg/kg.(83) 2-Mercapto-L-Cysteine **(181)**
815 extracted from *Allium sativum* L. bulbs using methanol. The compound provided 50% protection
816 against *Naja naja karachiensis* PLA2, evidenced by an increase in pH of an egg yolk suspension.
817 It also neutralized the anticoagulant effect induced by weak phospholipase A enzymes in *Naja naja*
818 *karachiensis* venom, achieving 40% inhibition with a coagulation time of 115 ± 1.52 s .(83)
819 Sativin I **(182)** extracted from *Allium sativum* L. bulbs using methanol, Sativin I exhibited
820 hepatoprotective activity, as assessed through AST and ALT enzyme levels. It provided 50%
821 protection against *Naja naja karachiensis* PLA2 by increasing the pH of an egg yolk suspension
822 and neutralizing its anticoagulant effects (40% inhibition, coagulation time of 115 ± 1.52 s).(83)
823 Sativin II **(183)** derived from *Allium sativum* L. bulbs using methanol, Sativin II displayed
824 hepatoprotective properties, as observed in AST and ALT enzymatic evaluations. It offered 50%
825 protection against *Naja naja karachiensis* PLA2, resulting in increased pH of an egg yolk
826 suspension and a 40% inhibition of the anticoagulant effect, with a coagulation time of 115 ± 1.52
827 s.(83)
828 8-Methoxycoumestrol **(184)**, present in low concentrations in *Medicago sativa* L., was prepared
829 as a sodium salt derivative. It mimicked wedelolactone by exhibiting antimyotoxic activity and
830 inhibiting edema, hemorrhagic effects, and cardiotoxicity induced by *Bothrops jararacussu* crude
831 venom.(132) 1-Hydroxytetratriacontane-4-One **(185)** isolated from *Leucas aspera* Linn.
832 methanolic leaf extract, this compound significantly neutralized the lethal activity of *Naja naja*
833 *naja* venom in a mouse model.(132) 3,4-Dihydroxyphenyllactic Acid **(186)**, a derivative of
834 rosmarinic acid, was first reported in *Cordia verbenacea*. It exhibited anti-inflammatory and
835 antimyotoxic properties against *Bothrops jararacussu* venom. It also inhibited the myotoxic and
836 oedema-inducing activities of Lys49 PLA2 and Asp49 isoforms from *B. jararacussu* venom.(132)
837 Caftaric Acid **(187)** exhibited neuromuscular blocking activity and inhibition of muscle damage
838 induced by Lys49-PLA2 (*BthTX-I*) from *Bothrops jararacussu* venom.(133) Chicoric Acid **(188)**
839 has demonstrated significant *in vitro* inhibitory effects on neuromuscular block and muscle
840 damage, as evidenced by structural and calorimetrical assays. It was analyzed in complex with
841 Lys49-PLA2 (BthTX-I) from *Bothrops jararacussu* venom through small angle X-ray scattering
842 and dynamic light scattering, providing insights into the oligomeric behavior of the PLA2 and acid
843 complex. The compound shows promise as a potential therapeutic agent in mitigating the effects
844 of snakebite toxins.(133)
845 Triacontyl p-Coumarate **(189)** as a derivative of p-Coumaric acid, triacontyl p-Coumarate (PCT)
846 demonstrated a 45% inhibition of the coagulant activity of *Bothrops pauloensis* venom in bovine
847 plasma. PCT effectively prevented fibrinogen degradation *in vitro* by SVMPs such as Jararhagin
848 and BleucMP, providing promising evidence of its potential in mitigating the harmful effects of
849 snake venom, particularly hemorrhagic activity.(133) 2-Hydroxy-4-methoxybenzaldehyde **(190)**,
850 isolated from *Janakia arayalpathra*, a plant used as an antidote in Western India, exhibits anti-
851 snake venom activity. It targets phospholipase A2 (PLA2), a major component of snake venom, and
852 holds potential as a therapeutic agent in treating venom-induced pathophysiological changes. Its
853 application could be valuable in developing new anti-snake venom treatments.(47)
854 2-Hydroxy-3-methoxybenzylalcohol **(191)** was found to have significant anti-venom activity,
855 including anti-hemorrhagic and PLA2 inhibitory effects, in *in vivo* and *in vitro* models. The
856 compound's activity highlights its potential in treating venom-induced pathophysiological
857 changes. Its structure, featuring a formyl group and a phenolic group, suggests it could be a
858 valuable lead in anti-snake venom drug development.(47)
859 2-OH-4-MeO Benzaldehyde **(192)**, also isolated from *Janakia arayalpathra*, displays inhibitory
860 activity against PLA2 enzymes found in snake venom. Its promising antivenom properties make
861 it a candidate for further investigation as a therapeutic agent against venom-induced
862 envenomations.(47) Coumestrol **(193)**, a coumestan derived from *Eclipta prostrata*, exhibits anti-
863 snake venom properties, particularly against the venom of *Bothrops jararacussu*. While less potent
864 than wedelolactone, coumestrol's antagonistic effects on creatine kinase release induced by venom
865 suggest it could serve as a potential therapeutic in snakebite treatment.(85)
866 Iso Butyl Phthalate **(194)**, although not detailed in its exact mechanism, has been identified as a
867 component with antivenom activities in various medicinal plants. It contributes to the broader class
868 of molecules that inhibit the toxic effects of snake venoms.(85) Dimercaprol **(195)**, part of a group
869 of small-molecule snake toxin inhibitors, has shown potential in clinical settings, particularly when
870 combined with other inhibitors like varespladib. The compound’s efficacy in neutralizing venom
871 toxins and its progression to Phase II clinical trials suggest that it may be a valuable therapeutic in
872 snakebite management.(85) Manoalide **(196)**, isolated from *Curcuma longa*, exhibits potent anti-
873 cobra PLA2 activity. Its therapeutic potential as a natural venom inhibitor positions it as a key
874 component in developing treatments for snakebite envenomations, particularly in cases involving
875 cobras.(134)
876 C20 Dialdehyde **(197)**, isolated from *Curcuma zedoaroides*, has demonstrated *in vitro* and *in vivo*
877 activity against King cobra venom. The compound effectively inhibits neuromuscular
878 transmission, offering significant promise for the treatment of venom-induced paralysis and other
879 physiological disruptions caused by snake venom.(109) Protocatechuic acid **(198)** was reported
880 for its cardioprotective and antihemolytic activities in the context of *Naja naja karachiensis* venom
881 exposure. Extracts containing this compound significantly stabilized red blood cell membranes
882 and inhibited venom phospholipase A2 (PLA2) activity, resulting in 76% coagulation
883 inhibition(10). Its antioxidant properties are believed to underlie its capacity to neutralize oxidative
884 and inflammatory components of venom, although cytotoxicity was noted at higher concentrations
885 (>160 µg/mL).
886 Ichangin-4-β-glucopyranoside **(199)**, a glycoside compound identified in the methanolic extract of
887 *C. limon* roots and ripe fruits, also demonstrated antivenom efficacy comparable to d-limonene.
888 The compound achieved 64% inhibition of PLA₂-mediated coagulation from *N. naja karachiensis*
889 venom, with a coagulation time of 109 ± 1.00 seconds. In parallel, it mitigated the lethal effect of
890 *L. muta* venom in vitro, with an ED₅₀ of 710 µg per mouse. These findings support its role as a
891 potential	lead	compound	in	venom	neutralization	strategies(10).	7-α-H-
892 Cyclopenta[a]cyclopropa[f]cycloundecene-2,4,7,7a,10,11-hexol **(200)**, derived from aqueous root
893 extracts of *Cynodon dactylon*, exhibited significant antihemolytic activity against crude *Naja naja*
894 venom, ranging from 84.3% to 90.6% inhibition across concentrations of 200 to 1,500 µg/ml. Such
895 potent membrane-stabilizing effects suggest its potential as a protective agent against venom-
896 induced	hemolysis	(10).	α-D-Glucopyranoside,	O-α-D-glucopyranosyl-(1→3)-α-D-
897 fructofuranosyl **(201)** a disaccharide compound, isolated from aqueous extracts of *C. dactylon*
898 roots, demonstrated comparable antihemolytic activity (84.3%–90.6%) against *N. naja* venom.
899 The sugar moiety's bioactivity may stem from its ability to chelate venom components or interfere
900 with cell membrane interactions, thus reducing venom-induced erythrocyte lysis(10).
901 9-Octadecenoic Acid (2-phenyl-1,3-dioxolan-4-yl)methyl Ester **(202),** identified in *C. dactylon*
902 aqueous root extract, this lipid-based ester showed robust antihemolytic action (84.3%–90.6%)
903 against *N. naja* venom. The amphipathic nature of this molecule likely contributes to its
904 membrane-protective activity by interacting with venom enzymes or lipid bilayers.(10)
905 9,10-Secocholesta-5,7,10(19)-triene-1,3-diol,25-[(trimethylsilyl)oxy] (3α,5Z,7E) **(203)**, derived
906 from *C. dactylon* root extract, significantly protected against hemolysis caused by crude *N. naja*
907 venom, with inhibition levels between 84.3% and 90.6%. Its triene structure and hydroxyl
908 substitutions may facilitate effective neutralization of venom phospholipases or pore-forming
909 toxins(10). Octasiloxane, 1,1,3,3,5,5,7,7,9,9,11,11,13,13,15,15-hexadecamethyl **(204)** exhibited
910 high antihemolytic potential (84.3%–90.6%) in response to *N. naja* venom exposure. Its
911 hydrophobic silicon-based backbone may inhibit venom-induced hemolysis by disrupting protein–
912 membrane interactions or stabilizing erythrocyte membranes.(10) Oximino-2,7-diethoxyfluorene
913 **(205)** isolated from the aqueous extract of *C. dactylon* roots, this fluorene derivative significantly
914 prevented hemolysis caused by *N. naja* venom *in vitro*. Its inhibition percentage (84.3%–90.6%)
915 underscores its effectiveness, potentially due to interference with venom-induced oxidative or lytic
916 pathways (10).
917 Hexadecanoic acid, 1-(hydroxymethyl)-1,2-ethanediyl ester **(206)**, identified from aqueous root
918 extracts of *Cynodon dactylon*, demonstrated significant antihemolytic activity, with inhibition
919 rates ranging from 84.3% to 90.6% against *Naja naja* venom at concentrations between 200 and
920 1,500 µg/mL. The compound’s potent membrane-stabilizing potential is presumed to underlie its
921 hemolytic suppression, although the exact mechanism remains to be elucidated.(10) Hexasiloxane,
922 1,1,3,3,5,5,7,7,9,9,11,11-dodecamethyl **(207)** also derived from the aqueous extract of *Cynodon*
923 *dactylon* roots, hexasiloxane exhibited similar antihemolytic efficacy (84.3%–90.6%) against
924 crude *Naja naja* venom. As a siloxane-based compound, its interaction with venom components
925 may be attributed to its potential to interfere with lipid bilayers or protein conformation.(10)
926 Heptasiloxane, 1,1,3,3,5,5,7,7,9,9,11,11,13,13-tetradecamethyl **(208)** isolated from *C. dactylon*
927 aqueous root extract, heptasiloxane showed comparable antihemolytic activities (84.3% to 90.6%).
928 These findings highlight the potential therapeutic value of siloxane derivatives as inhibitors of
929 hemolysis, possibly via membrane-protective or surfactant-like behavior.(10)
930 Estra-1,3,5(10)-trien-17-α-ol **(209)** exhibited significant inhibition of venom-induced hemolysis
931 when tested in the same concentration range as other compounds from *Cynodon dactylon*. Its
932 structural similarity to hormones suggests possible interference with venom enzymes or oxidative
933 pathways. Further pharmacological evaluations are required to determine its systemic safety and
934 efficacy.(10) 3,3′,5′-Trimethoxy-4,5-methylenedioxy dihydrostilbene **(210)** isolated from the
935 hexane fraction of aerial parts of *Indigofera capitata* (Fabaceae), this novel dihydrostilbene
936 displayed *in vivo* antivenom activity against *Naja nigricollis* venom in murine models, with
937 survival rates of 50% and 16.5% at 1 mg/kg and 5 mg/kg, respectively. The compound is a
938 promising lead candidate for development of antivenom therapeutics due to its observed protective
939 efficacy.(135)
940 3,5-Dimethoxy-4-hydroxyphenylacetic acid **(211)** from *A. parvifolia*, this phenolic acid
941 constituted 5.30% of the extract’s content and likely contributes to the antioxidant and anti-PLA2
942 effects observed. Phenolic compounds are known to inhibit venom enzymatic activity and reduce
943 oxidative stress, enhancing their potential in venom neutralization.(136) Acetylmarinobufogenin
944 **(212)** a dominant compound in the bark extract (14.89%), acetylmarinobufogenin showed strong
945 inhibitory potential against PLA2 venom enzymes with docking energies (−7.22 to −9.59
946 kcal/mol). Its steroidal structure may enhance lipophilic interaction with enzymatic active sites,
947 aiding venom neutralization.(136) γ-Sitosterol **(213)** representing 10.44% of the extract’s content,
948 γ-sitosterol is a triterpenoid with known anti-inflammatory and membrane-stabilizing properties.
949 While its specific antivenom mechanism is yet to be fully elucidated, its presence contributed to
950 mitigating venom-induced pathophysiological changes.(136) (**Supplementary File; Table S1 and**

## 951 Figure S1).

952 **3.3 Computational Analysis of ADMET Properties, Medicinal Chemistry, and Drug-likeness.**
953 To evaluate and predict the ADMET profiles, drug-likeness, and medicinal chemistry properties
954 of 213 plant-derived compounds tested against various snake venoms and toxins targets, the
955 compounds were grouped based on their chemical classes. Table 1 provides a summary of the
956 number of molecules that progressed through each stage and the final selection of compounds with
957 strong pharmacokinetic and pharmacological potential.
958 **Table 1**. Assessment of drug-likeness, ADMET characteristics, and medicinal chemistry
959 parameters of plant-derived compounds with inhibitory potential against different snake
960 venom/toxin targets.

962

| **Compound Class** | **DL** | **A** | **D** | **M** | **E** | **T** | **MC** |
| --- | --- | --- | --- | --- | --- | --- | --- |
| **Compounds Failed/ Passed** | | | | | | | |
| Alkaloids (17) | **5/12** | **7/5** | **3/2** | **0/2** | **1/1** | **1/0** |  |
| Benzenoids (9) | **0/9** | **3/6** | **2/4** | **1/3** | **2/1** | **0/1** | **0/1** |
| Hydroxycinnamic Acids  (8) | **1/7** | **3/4** | **2/2** | **0/2** | **1/1** | **0/1** | **0/1** |
| Tannins (5) | **2/3** | **2/1** | **1/0** |  |  |  |  |
| Coumarins (3) | **0/3** | **0/3** | **1/2** | **1/1** | **0/1** | **1/0** |  |
| Flavonoids (32) | **12/20** | **10/10** | **5/5** | **2/3** | **2/1** | **1/0** |  |

| Isoflavonoids (10) | **1/9** | **3/6** | **2/4** | **3/1** | **0/1** | **0/1** | **1/0** |
| --- | --- | --- | --- | --- | --- | --- | --- |
| Modified Glycosides (8) | **7/1** | **1/0** |  |  |  |  |  |
| Polyketides (4) | **0/4** | **1/3** | **3/0** |  |  |  |  |
| Terpeneoids (37) | **29/8** | **4/4** | **2/2** | **0/2** | **0/2** | **0/2** | **0/2** |
| Saponins (10) | **10/0** |  |  |  |  |  |  |
| Other Compounds (70) | **49/21** | **13/8** | **4/4** | **1/3** | **0/3** | **2/1** | **0/1** |
| Total Compounds (213) | **97** | **50** | **25** | **17** | **11** | **6** | **5** |
| Best Compounds |  |  |  |  |  |  | **3** |

963

964	**Legend: (**DL) Druglikeness; (A) Absorption; (D) Distribution; (M) Metabolism; (E) Excretion;

965	(T) Toxicity; (MC) Medicinal chemistry parameters

966

## 967 Drug-Likeness Evaluation of the Compounds

968 Drug-likeness assessments were initially performed using SwissADME, ADMETlab and
969 DataWarrior. This analysis evaluated the molecules based on Lipinski, Veber, Ghose, Egan, and
970 Muegge rules.(17,137–139) Molecules failing to meet the drug-likeness criteria specifically those
971 violating more than one rule (Veber and Egan) or more than two rules (Lipinski, Ghose, and
972 Muegge) were excluded from further consideration. Out of the 213 molecules classified into 11
973 chemical groups in this study, 97 passed the drug-likeness evaluation. The highest success rates
974 were observed in the coumarin, Benzenoids, Polyketides (100%) and Isoflavonoids (90%) classes,
975 whereas the saponin (0%) class had the lowest number of passed compounds.
976 The high approval rate of coumarin, benzenoids, isoflavonoids, and polyketides can be attributed
977 to their relatively small and stable structures, which align with key pharmaceutical viability
978 criteria, enhancing their bioavailability and systemic distribution.(140,141) Moreover, the
979 structural diversity within these classes enables chemical modifications that can further improve
980 their pharmacokinetic properties.(141) In contrast, saponins exhibit large, complex molecular
981 structures, often surpassing 500 Da in molecular weight, with high topological polar surface area
982 (TPSA) values due to multiple hydrophilic functional groups.(139,142) Saponins also possess
983 numerous	hydrogen	bond	donors	and	acceptors,	which	significantly	limit	their
984 permeability.(141,143)

| 985 | The findings from this initial stage are highly significant, as molecules that fail to meet drug- |
| --- | --- |
| 986 | likeness criteria generally have a lower likelihood of becoming successful drug candidates and can |
| 987 | therefore be excluded early in the selection process. For the subsequent evaluations, absorption, |
| 988 | distribution, metabolism, and excretion, computational tools (SwissADME, pkCSM, and |
| 989 | ADMETlab) were employed. However, the specific parameters assessed were adjusted to match |
| 990 | the focus of each stage. At every step, any molecule that violated one or more criteria was |
| 991 | eliminated. |
| 992 | **Absorption Phase** |
| 993 | During the absorption assessment, key factors influencing a drug's effectiveness were examined, |
| 994 | including oral bioavailability, permeability in human colon epithelial carcinoma cells (Caco-2), |
| 995 | water solubility, and human intestinal absorption (HIA). About 50 compounds passed this |
| 996 | screening, with alkaloids showing the highest retention rates. Alkaloids, characterized by high |
| 997 | polarity and structural complexity, tend to have reduced permeability in Caco-2 cells, resulting in |
| 998 | low bioavailability.(144) |
| 999 | **Distribution Phase** |
| 1000 | The distribution phase was assessed using parameters such as volume of distribution (VDss), |
| 1001 | plasma protein binding (PPB), fraction unbound in plasma (Fu), and blood-brain barrier |
| 1002 | permeability (BBB). Following this screening, 25 molecules demonstrated favorable distribution |
| 1003 | properties and strong potential to reach their target sites. Compounds with optimal distribution |
| 1004 | typically exhibit moderate LogP values, balanced molecular weight, and adequate water solubility. |
| 1005 | These features indicate that a molecule has sufficient lipophilicity to cross biological membranes |
| 1006 | while avoiding excessive accumulation in fatty tissues. Additionally, these properties enhance |
| 1007 | cellular permeability and tissue distribution, allowing the compound to reach its intended site of |
| 1008 | action.(145,146) |
| 1009 | **Metabolism Phase** |
| 1010 | In the metabolism phase, the primary focus was on interactions with cytochrome P450 (CYP450) |
| 1011 | enzymes. Molecules that neither strongly inhibit nor induce major CYP isoforms (such as |

| 1012 | CYP1A2, CYP3A4, CYP2C9, CYP2C19, and CYP2D6) are considered preferable, as significant |
| --- | --- |
| 1013 | interference with these enzymes can result in harmful drug interactions. Additionally, compounds |
| 1014 | that undergo rapid metabolism may have reduced efficacy, while those that are metabolized too |
| 1015 | slowly can accumulate in the body, potentially leading to toxic effects.(147) At this stage, |
| 1016 | interactions with cytochrome P450 (CYP450) enzymes were analyzed, and compounds that |
| 1017 | exhibited significant interactions with three or more CYP isoforms were eliminated. As a result, |
| 1018 | the number of compounds in the screening process was reduced to 17. |
| 1019 | **Excretion Phase** |
| 1020 | Following metabolism evaluation, the excretion phase was assessed, focusing on elimination half- |
| 1021 | life and total clearance. In this final stage of the ADME evaluation, compounds with a moderate |
| 1022 | half-life (1–12 hours) and a balanced total clearance rate (2–5 mL/min/kg) were considered |
| 1023 | suitable, as these properties support both convenient dosing schedules and effective plasma |
| 1024 | concentration management. This balance enhances treatment adherence and minimizes the |
| 1025 | likelihood of adverse effects between doses.(148) Ultimately, only 11 molecules passed this stage. |
| 1026 | The reason for this is that compounds with either excessively long or very short half-lives pose |
| 1027 | significant challenges. Those with prolonged half-lives can accumulate in the body, increasing the |
| 1028 | risk of toxicity such as nephrotoxicity or hepatotoxicity due to inefficient elimination.(149,150) |
| 1029 | Conversely, compounds with extremely short half-lives are rapidly cleared, necessitating frequent |
| 1030 | administration to maintain therapeutic levels, which can negatively impact patient adherence.(149) |
| 1031 | Moreover, total clearance plays a critical role in drug efficacy and safety. A high clearance rate can |
| 1032 | lead to insufficient drug levels, reducing effectiveness, while a low clearance rate may result in |
| 1033 | accumulation and potential toxicity.(149) Understanding the excretion mechanisms of a drug is |
| 1034 | crucial for predicting and managing drug interactions, ensuring patient safety, and preventing |
| 1035 | adverse effects caused by inadequate elimination. (149,151)Therefore, maintaining an optimal |
| 1036 | range for both half-life and total clearance is essential for achieving safe and effective drug |
| 1037 | excretion, ensuring successful therapeutic outcomes. |
| 1038 | **Toxicity Assessment** |

| 1039 | In the final stage, the molecules underwent toxicity screening using pkCSM, ProTox3, Toxtree, |
| --- | --- |
| 1040 | and DataWarrior. This step was crucial to assess the safety of the compounds before proceeding |
| 1041 | with further analyses. Twelve potential toxic effects were considered, including hepatotoxicity, |
| 1042 | neurotoxicity, nephrotoxicity, cardiotoxicity, and respiratory toxicity, along with carcinogenicity, |
| 1043 | immunotoxicity, mutagenicity, cytotoxicity, hematotoxicity, predicted acute oral toxicity in rats, |
| 1044 | and clinical toxicity. Any compound exhibiting potential toxicity in four or more of these |
| 1045 | categories was eliminated. Following this assessment, the number of viable molecules was reduced |
| 1046 | to 6, resulting in the elimination of the alkaloid, coumarins and flavonoids. |
| 1047 | Natural products often struggle in the toxicity evaluation stage of ADMET due to their complex |
| 1048 | and diverse chemical structures, which can lead to a broad spectrum of adverse effects. Alkaloids, |
| 1049 | in particular, are known for their high biological activity, which is often linked to potential toxicity. |
| 1050 | (152) These compounds can undergo metabolic transformations that produce toxic metabolites and |
| 1051 | may interfere with liver enzymes and renal transport mechanisms, leading to organ damage.(153) |
| 1052 | Additionally, alkaloids, coumarins and flavonoids pose a significant risk of neurotoxicity and |
| 1053 | cardiotoxicity due to their ability to inhibit sodium and potassium channels, which are critical for |
| 1054 | neuromuscular and cardiac function.(152,153) Because of these risks, despite their therapeutic |
| 1055 | potential, these compounds require thorough toxicity screening to ensure their safe application in |
| 1056 | clinical settings.(144) |
| 1057 | **Medicinal Chemistry Filtering** |
| 1058 | Beyond toxicity assessments, additional medicinal chemistry filters were applied. These included |
| 1059 | screening for potential pan-assay interference compounds (PAINS) or invalid metabolic panaceas |
| 1060 | (IMPS), where compounds with two or more infractions were eliminated. Similarly, Brenk alerts |
| 1061 | with three or more violations and lead-likeness infractions of two or more were used as exclusion |
| 1062 | criteria. Furthermore, a synthetic accessibility score was evaluated on a scale from 1 (very easy) |
| 1063 | to 10 (very difficult), with molecules scoring 5 or higher being removed from consideration. These |
| 1064 | analyses were conducted using SwissADME, and DataWarrior. Any violation in any of these four |
| 1065 | categories led to the elimination of the compound. At this stage, one compound belonging to |
| 1066 | Isoflavonoids was excluded. Certain alerts suggested a high likelihood of false positives in |

| 1067 | biological assays, along with potential limitations in Isoflavonoids’ effectiveness as a lead |
| --- | --- |
| 1068 | molecule and other developmental challenges. |
| 1069 | Assessing medicinal chemistry parameters such as PAINS, Brenk alerts, lead-likeness, and |
| 1070 | synthetic accessibility is essential in drug development, as these factors help identify compounds |
| 1071 | prone to undesirable effects or obstacles in further development.(21) A rigorous evaluation of these |
| 1072 | parameters during the early screening phase reduces risks and costs, ensuring that only the most |
| 1073 | promising compounds advance to later drug development stages.(21) |
| 1074 | **Final Selection Based on Biological Activity** |
| 1075 | In the final selection, compounds with inhibition values of 50% or higher against at least one snake |
| 1076 | venom/toxin target were manually chosen. Only three molecules met these criteria: anisic acid **23** |
| 1077 | (benzenoid) as well as labdane lactone (**97)** and labdane trialdehyde (**98)** both classified as terpenes |
| 1078 | while hydroxycinnamic acids and other compounds classes failed this criterion leading to their |
| 1079 | elimination. These compounds demonstrated strong biological activity, anisic acid (**23**) |
| 1080 | demonstrated 100% neutralization of the lethal effects and defibrinogenation induced by the |
| 1081 | venoms of *D. russelii, E. carinatus, N. kaouthia*, and *O. hannah* in both *in vitro* and *in vivo* |
| 1082 | studies.(1) Compounds **97** and **98**, isolated from *C. antinaia* and *C. zedoaroides*, respectively, |
| 1083 | exhibited significant venom inhibition at a concentration of 10 µg/mL. Specifically, Compound **97** |
| 1084 | demonstrated an 83% inhibition rate, while Compound **98** showed a 62% inhibition rate against |
| 1085 | *O. hannah* venom.(1) Notably, compound **98**, preserved diaphragmatic contraction almost entirely, |
| 1086 | offering 99% protection. This makes it the most promising candidate for antivenom and anti- |
| 1087 | neurotoxic therapy.(106) |
| 1088 | These findings highlight that, despite numerous tested molecules showing promising inhibitory |
| 1089 | potential against certain snake venoms and toxins targets, only a few exhibited complete viabilities |
| 1090 | as drug candidates without requiring structural modifications. However, given the strong bioactive |
| 1091 | potential of some compounds, it is worth considering the cost-benefit of making structural |
| 1092 | adjustments to optimize their properties. By refining their core structures, these molecules could |
| 1093 | be adapted to meet essential criteria such as druglikeness, ADMET properties, and other medicinal |
| 1094 | chemistry parameters that may have led to their exclusion. This screening process enabled the |

| 1095 | precise selection of compounds, ensuring that the approved molecules not only possessed |
| --- | --- |
| 1096 | favorable ADMET characteristics but also had a strong likelihood of progressing into viable drug |
| 1097 | candidates. This step is essential for prioritizing compounds that are both biologically potent and |
| 1098 | chemically feasible for large-scale synthesis and pharmaceutical development. The filtering |
| 1099 | strategy effectively eliminated molecules with less favorable profiles, concentrating on those with |
| 1100 | significant inhibitory activity against snake venom targets while maintaining highly desirable |
| 1101 | pharmacological properties. |
| 1102 | **Conclusion** |
| 1103 | This systematic review explored a broad range of plant-derived metabolites with potential |
| 1104 | inhibitory effects on key components of snake venom. Many of these compounds demonstrated |
| 1105 | significant inhibition, with IC50 values below 10 μM in several cases, suggesting their strong |
| 1106 | therapeutic potential against venom and its toxic effects. However, despite their potent activity, the |
| 1107 | predicted *in silico* ADMET profiles of these compounds were unfavorable in this study. From an |
| 1108 | initial selection of 213 compounds, only three met the stringent criteria for druglikeness, ADMET |
| 1109 | properties, and essential medicinal chemistry and biological activity parameters, reinforcing their |
| 1110 | viability as drug candidates. While other chemical classes, including flavonoids, tannins, and |
| 1111 | alkaloids, have recognized antivenom properties, they were excluded due to suboptimal ADMET |
| 1112 | profiles in this study or insufficient inhibitory effects in the assays reviewed from the literature. |
| 1113 | In research that employs ADMET profiling as a screening tool, the elimination of many molecules |
| 1114 | due to unfavorable characteristics is common. However, this should not be seen as a definitive |
| 1115 | barrier. Compounds with poor ADMET profiles can still be optimized through various methods, |
| 1116 | such as refining therapeutic targets, modifying their chemical structures to improve |
| 1117 | pharmacokinetic properties, and implementing monitoring strategies to mitigate adverse effects. |
| 1118 | Moreover, advanced formulation techniques, including controlled-release delivery systems and |
| 1119 | nanocarriers, can enhance bioavailability and distribution. These strategies are widely used, even |
| 1120 | for clinically established drugs, demonstrating that molecules initially considered unsuitable can |
| 1121 | be modified for effective therapeutic use. The screening process in this review was designed to |
| 1122 | identify natural molecules with inherent biological activity, favorable druglikeness, optimized |
| 1123 | ADMET properties, and strong medicinal chemistry attributes. This approach streamlines the |

| 1124 | discovery of promising natural products with pharmaceutical potential, reducing both the financial |
| --- | --- |
| 1125 | burden and time investment needed for drug research and development. |
| 1126 | This study provided an in-depth evaluation of the pharmacokinetic and toxicological properties of |
| 1127 | these compounds, emphasizing their potential for drug development. The results underscore the |
| 1128 | relevance of natural products as promising sources for antivenom agents and provide valuable |
| 1129 | leads for the development of new antivenom treatments. Additionally, the study highlights the |
| 1130 | necessity of further exploration of plant-derived compounds, incorporating *in silico*, *in vitro*, and |
| 1131 | *in vivo* analyses to validate and refine their effectiveness. Ultimately, this research establishes a |
| 1132 | solid foundation for future investigations aimed at discovering and formulating effective |
| 1133 | antivenom agents from natural sources. The comprehensive literature review and rigorous |
| 1134 | screening of plant metabolites presented in this study contribute significantly to the field, offering |
| 1135 | critical insights into their potential therapeutic applications against venom toxicity. |
| 1136 | **Data Availability** |
| 1137 | All relevant data are within the manuscript and its Supporting Information files. |
| 1138 | **Conflicts of Interest** |
| 1139 | The authors affirm that there are no conflicts of interest. |
| 1140 | **Authors’ Contributions** |
| 1141 | Prince Ojuka: Conceived the idea, literature review, software, and drafted the original manuscript. |
| 1142 | George Simba Nyamato, and Njogu M. Kimani: Conception, software, methodology, manuscript |
| 1143 | review and editing. Lucy Ochola and George Omondi: Software, methodology and manuscript |
| 1144 | review and editing. All authors examined and gave their approval for the final version of the article |
| 1145 | for publication. Cleydson B.R. Santos was responsible for the *in silico* ADMET analysis, and |
| 1146 | interpreted computational findings. |
| 1147 |  |
| 1148 |  |

| 1149 | **References** |
| --- | --- |
| 1150 | 1. Adrião AAX, Dos Santos AO, de Lima EJSP, Maciel JB, Paz WHP, da Silva FMA, et al. |
| 1151 | Plant-derived toxin inhibitors as potential candidates to complement antivenom treatment |
| 1152 | in snakebite envenomations. Front Immunol. 2022;13:842576. |
| 1153 | 2. Williams DJ, Faiz MA, Abela-Ridder B, Ainsworth S, Bulfone TC, Nickerson AD, et al. |
| 1154 | Strategy for a globally coordinated response to a priority neglected tropical disease: |
| 1155 | Snakebite envenoming. PLoS Negl Trop Dis. 2019;13(2):e0007059. |
| 1156 | 3. Yue S, BoneBrake TC, GiBSon L. Human-snake conflict patterns in a dense urban-forest |
| 1157 | mosaic landscape. Herpetol Conserv Biol. 2019;14(1):143–54. |
| 1158 | 4. Chippaux J-P. Snakebite envenomation turns again into a neglected tropical disease! J |
| 1159 | Venom Anim Toxins Incl Trop Dis. 2017;23:38. |
| 1160 | 5. Bolon I, Durso AM, Botero Mesa S, Ray N, Alcoba G, Chappuis F, et al. Identifying the |
| 1161 | snake: First scoping review on practices of communities and healthcare providers |
| 1162 | confronted with snakebite across the world. PLoS One. 2020;15(3):e0229989. |
| 1163 | 6. Gutiérrez JM, Calvete JJ, Habib AG, Harrison RA, Williams DJ, Warrell DA. Snakebite |
| 1164 | envenoming. Nat Rev Dis Prim. 2017;3(1):1–21. |
| 1165 | 7. Habib AG, Kuznik A, Hamza M, Abdullahi MI, Chedi BA, Chippaux J-P, et al. Snakebite |
| 1166 | is under appreciated: appraisal of burden from West Africa. PLoS Negl Trop Dis. |
| 1167 | 2015;9(9):e0004088. |
| 1168 | 8. Resiere D, Monteiro W, Houcke S, Pujo JM, Mathien C, Mayence C, et al. Bothrops |
| 1169 | snakebite envenomings in the Amazon Region. Curr Trop Med Reports. 2020;7:48–60. |
| 1170 | 9. Gutiérrez JM, Albulescu L-O, Clare RH, Casewell NR, Abd El-Aziz TM, Escalante T, et |
| 1171 | al. The search for natural and synthetic inhibitors that would complement antivenoms as |
| 1172 | therapeutics for snakebite envenoming. Toxins (Basel). 2021;13(7):451. |

| 1173 | 10. Omara T, Kiwanuka Nakiguli C, Naiyl RA, Atieno Opondo F, Otieno SB, Ndiege ML, et |
| --- | --- |
| 1174 | al. Medicinal plants used as snake venom antidotes in East African Community: review |
| 1175 | and assessment of scientific evidences. 2021; |
| 1176 | 11. Opiyo SA, Njoroge PW. Plant Extracts and Terpenes with Antivenom Properties. |
| 1177 | 2024;17(3):31–41. |
| 1178 | 12. Maciel B, Monteiro WM, Sartim MA, Koolen HHF. Plant-Derived Toxin Inhibitors as |
| 1179 | Potential Candidates to Complement Antivenom Treatment in Snakebite Envenomations. |
| 1180 | 2022;13(May):1–28. |
| 1181 | 13. Araújo JL, Sousa LA de, Sousa AO, Bastos RS, Santos GT, Lage MR, et al. DFT, |
| 1182 | molecular docking, and ADME/Tox screening investigations of Market-Available drugs |
| 1183 | against SARS-CoV-2. J Braz Chem Soc. 2021;32(8):1628–41. |
| 1184 | 14. Srivastava V, Yadav A, Sarkar P. Molecular docking and ADMET study of bioactive |
| 1185 | compounds of Glycyrrhiza glabra against main protease of SARS-CoV2. Mater Today |
| 1186 | Proc. 2022;49:2999–3007. |
| 1187 | 15. Ojuka P, Ochieng CO, Ndarawit W, Nyongesa DW, Mukavi J, Nyariki JN, et al. |
| 1188 | Alkaloids Isolated from Vepris glandulosa with Antidiabetic Properties: An In Vitro and |
| 1189 | In Silico Analysis. Chem Biodivers. :e202401515. |
| 1190 | 16. Khaldan A, Bouamrane S, En-Nahli F, El-Mernissi R, Hmamouchi R, Maghat H, et al. |
| 1191 | Prediction of potential inhibitors of SARS-CoV-2 using 3D-QSAR, molecular docking |
| 1192 | modeling and ADMET properties. Heliyon. 2021;7(3). |
| 1193 | 17. Ojuka P, Kimani NM, Apollo S, Nyariki J, Ramos RS, Santos CBR. Phytochemistry of |
| 1194 | the Vepris genus plants: A review and in silico analysis of their ADMET properties. South |
| 1195 | African J Bot. 2023;157:106–14. |
| 1196 | 18. Jorge RJB, Martins RD, Araújo RM, da Silva MA, Monteiro HSA, Ximenes RM. Plants |
| 1197 | and phytocompounds active against Bothrops venoms. Curr Top Med Chem. |
| 1198 | 2019;19(22):2003–31. |

| 1199 | 19. Page MJ, McKenzie JE, Bossuyt PM, Boutron I, Hoffmann TC, Mulrow CD, et al. The |
| --- | --- |
| 1200 | PRISMA 2020 statement: an updated guideline for reporting systematic reviews. bmj. |
| 1201 | 2021;372. |
| 1202 | 20. Tian S, Wang J, Li Y, Li D, Xu L, Hou T. The application of in silico drug-likeness |
| 1203 | predictions in pharmaceutical research. Adv Drug Deliv Rev. 2015;86:2–10. |
| 1204 | 21. Ferreira LLG, Andricopulo AD. ADMET modeling approaches in drug discovery. Drug |
| 1205 | Discov Today. 2019;24(5):1157–65. |
| 1206 | 22. Kar S, Leszczynski J. Open access in silico tools to predict the ADMET profiling of drug |
| 1207 | candidates. Expert Opin Drug Discov. 2020;15(12):1473–87. |
| 1208 | 23. Calvete JJ, Lomonte B. Present and Future of Snake Venom Proteomics Profiling. In: |
| 1209 | Handbook of Venoms and Toxins of Reptiles. CRC Press; 2021. p. 19–28. |
| 1210 | 24. Sanhajariya S, Isbister GK, Duffull SB. The influence of the different disposition |
| 1211 | characteristics of snake toxins on the pharmacokinetics of snake venom. Toxins (Basel). |
| 1212 | 2020;12(3):188. |
| 1213 | 25. Tasoulis T, Isbister GK. A review and database of snake venom proteomes. Toxins |
| 1214 | (Basel). 2017;9(9):290. |
| 1215 | 26. Luna MS, Valente RH, Perales J, Vieira ML, Yamanouye N. Activation of Bothrops |
| 1216 | jararaca snake venom gland and venom production: a proteomic approach. J Proteomics. |
| 1217 | 2013;94:460–72. |
| 1218 | 27. Ferraz CR, Arrahman A, Xie C, Casewell NR, Lewis RJ, Kool J, et al. Multifunctional |
| 1219 | toxins in snake venoms and therapeutic implications: from pain to hemorrhage and |
| 1220 | necrosis. Front Ecol Evol. 2019;7:218. |
| 1221 | 28. Ojeda PG, Ramírez D, Alzate-Morales J, Caballero J, Kaas Q, González W. |
| 1222 | Computational studies of snake venom toxins. Toxins (Basel). 2017;10(1):8. |

| 1223 | 29. Oliveira ICF, de Paula MO, Lastra HCB, Alves B de B, Moreno DAN, Yoshida EH, et al. |
| --- | --- |
| 1224 | Activity of silver nanoparticles on prokaryotic cells and Bothrops jararacussu snake |
| 1225 | venom. Drug Chem Toxicol. 2019;42(1):60–4. |
| 1226 | 30. Monteiro WM, Contreras-Bernal JC, Bisneto PF, Sachett J, da Silva IM, Lacerda M, et al. |
| 1227 | Bothrops atrox, the most important snake involved in human envenomings in the amazon: |
| 1228 | How venomics contributes to the knowledge of snake biology and clinical toxinology. |
| 1229 | Toxicon X. 2020;6:100037. |
| 1230 | 31. Moura-da-Silva AM, Contreras-Bernal JC, Cirilo Gimenes SN, Freitas-de-Sousa LA, |
| 1231 | Portes-Junior JA, da Silva Peixoto P, et al. The relationship between clinics and the |
| 1232 | venom of the causative Amazon pit viper (Bothrops atrox). PLoS Negl Trop Dis. |
| 1233 | 2020;14(6):e0008299. |
| 1234 | 32. Akunyili DN, Akubue PI. Schumanniofoside, the antisnake venom principle from the stem |
| 1235 | bark of Schumanniophyton magnificum Harms. J Ethnopharmacol. 1986;18(2):167–72. |
| 1236 | 33. Maria de Fátima CB, Cintra ACO, Veronese ELG, Lavrador MAS, Giglio JR, Pereira PS, |
| 1237 | et al. Inhibition of the lethal and myotoxic activities of Crotalus durissus terrificus venom |
| 1238 | by Tabernaemontana catharinensis: identification of one of the active components. Planta |
| 1239 | Med. 2000;66(05):424–8. |
| 1240 | 34. Torres MCM, Pinto F das CL, Braz-Filho R, Silveira ER, Pessoa ODL, Jorge RJB, et al. |
| 1241 | Antiophidic solanidane steroidal alkaloids from Solanum campaniforme. J Nat Prod. |
| 1242 | 2011;74(10):2168–73. |
| 1243 | 35. Torres MCM, Jorge RJB, Ximenes RM, Alves NTQ, Santos JV de A, Marinho AD, et al. |
| 1244 | Solanidane and iminosolanidane alkaloids from Solanum campaniforme. Phytochemistry. |
| 1245 | 2013;96:457–64. |
| 1246 | 36. Chandra DN, Prasanth GK, Singh N, Kumar S, Jithesh O, Sadasivan C, et al. |
| 1247 | Identification of a novel and potent inhibitor of phospholipase A2 in a medicinal plant: |
| 1248 | crystal structure at 1.93 Å and Surface Plasmon Resonance analysis of phospholipase A2 |

| 1249 | complexed with berberine. Biochim Biophys Acta (BBA)-Proteins Proteomics. |
| --- | --- |
| 1250 | 2011;1814(5):657–63. |
| 1251 | 37. Bhattacharjee P, Bera I, Chakraborty S, Ghoshal N, Bhattacharyya D. Aristolochic acid |
| 1252 | and its derivatives as inhibitors of snake venom L-amino acid oxidase. Toxicon. |
| 1253 | 2017;138:1–17. |
| 1254 | 38. Fernandes CAH, Cardoso FF, Cavalcante WGL, Soares AM, Dal-Pai M, Gallacci M, et al. |
| 1255 | Structural basis for the inhibition of a phospholipase A2-like toxin by caffeic and |
| 1256 | aristolochic acids. PLoS One. 2015;10(7):e0133370. |
| 1257 | 39. Tijjani H, Egbuna C. Medicinal Plants with Antivenom Activities. In: Phytochemistry. |
| 1258 | Apple Academic Press; 2018. p. 207–56. |
| 1259 | 40. Shenoy PA, Nipate SS, Sonpetkar JM, Salvi NC, Waghmare AB, Chaudhari PD. Anti- |
| 1260 | snake venom activities of ethanolic extract of fruits of Piper longum L.(Piperaceae) |
| 1261 | against Russell’s viper venom: characterization of piperine as active principle. J |
| 1262 | Ethnopharmacol. 2013;147(2):373–82. |
| 1263 | 41. Gomes A, Das R, Sarkhel S, Mishra R, Mukherjee S, Bhattacharya S, et al. Herbs and |
| 1264 | herbal constituents active against snake bite. 2010; |
| 1265 | 42. Sebastin Santhosh M, Hemshekhar M, Sunitha K, M Thushara R, Jnaneshwari S, |
| 1266 | Kemparaju K, et al. Snake venom induced local toxicities: plant secondary metabolites as |
| 1267 | an auxiliary therapy. Mini Rev Med Chem. 2013;13(1):106–23. |
| 1268 | 43. Alam MI, Gomes A. An experimental study on evaluation of chemical antagonists |
| 1269 | induced snake venom neutralization. Indian J Med Res. 1998;107:142. |
| 1270 | 44. Alam MI, Auddy B, Gomes A. Isolation, purification and partial characterization of viper |
| 1271 | venom inhibiting factor from the root extract of the Indian medicinal plant sarsaparilla |
| 1272 | (Hemidesmus indicus R. Br.). Toxicon. 1994;32(12):1551–7. |
| 1273 | 45. Alam MI, Gomes A. Viper venom-induced inflammation and inhibition of free radical |

| 1274 | formation by pure compound (2-hydroxy-4-methoxy benzoic acid) isolated and purified |
| --- | --- |
| 1275 | from anantamul (Hemidesmus indicus R. BR) root extract. Toxicon. 1998;36(1):207–15. |
| 1276 | 46. Nargotra A, Sharma S, Alam MI, Ahmed Z, Bhagat A, Taneja SC, et al. In silico |
| 1277 | identification of viper phospholipaseA2 inhibitors: validation by in vitro, in vivo studies. J |
| 1278 | Mol Model. 2011;17:3063–73. |
| 1279 | 47. Alam MI, Alam MA, Alam O, Nargotra A, Taneja SC, Koul S. Molecular modeling and |
| 1280 | snake venom phospholipase A2 inhibition by phenolic compounds: Structure–activity |
| 1281 | relationship. Eur J Med Chem. 2016;114:209–19. |
| 1282 | 48. Uma B, Gowda TV. Molecular mechanism of lung hemorrhage induction by VRV-PL- |
| 1283 | VIIIa from Russell’s viper (Vipera russelli) venom. Toxicon. 2000;38(8):1129–47. |
| 1284 | 49. H Mahadeswaraswamy Y, S Kumar M, J Gowtham Y, Nagaraju S, S Girish K, Kemparaju |
| 1285 | K. The polyphenol 3, 4, 5-tri-hydroxy benzoic acid inhibits Indian Daboia russelli venom |
| 1286 | and its hemorrhagic complex induced local toxicity. Curr Top Med Chem. |
| 1287 | 2011;11(20):2520–30. |
| 1288 | 50. Pereanez JA, Nunez V, Patino AC, Londono M, Quintana JC. Inhibitory effects of plant |
| 1289 | phenolic compounds on enzymatic and cytotoxic activities induced by a snake venom |
| 1290 | phospholipase A2. Vitae. 2011;18(3):295–304. |
| 1291 | 51. Dhananjaya BL, Nataraju A, Rajesh R, Gowda CDR, Sharath BK, Vishwanath BS, et al. |
| 1292 | Anticoagulant effect of Naja naja venom 5′ nucleotidase: demonstration through the use of |
| 1293 | novel specific inhibitor, vanillic acid. Toxicon. 2006;48(4):411–21. |
| 1294 | 52. Ticli FK, Hage LIS, Cambraia RS, Pereira PS, Magro ÂJ, Fontes MRM, et al. Rosmarinic |
| 1295 | acid, a new snake venom phospholipase A2 inhibitor from Cordia verbenacea |
| 1296 | (Boraginaceae): antiserum action potentiation and molecular interaction. Toxicon. |
| 1297 | 2005;46(3):318–27. |
| 1298 | 53. Aung HT, Nikai T, Komori Y, Nonogaki T, Niwa M, Takaya Y. Biological and |
| 1299 | pathological studies of rosmarinic acid as an inhibitor of hemorrhagic Trimeresurus |

| 1300 | flavoviridis (habu) venom. Toxins (Basel). 2010;2(10):2478–89. |
| --- | --- |
| 1301 | 54. Aung HT, Furukawa T, Nikai T, Niwa M, Takaya Y. Contribution of cinnamic acid |
| 1302 | analogues in rosmarinic acid to inhibition of snake venom induced hemorrhage. Bioorg |
| 1303 | Med Chem. 2011;19(7):2392–6. |
| 1304 | 55. Aung HT, Nikai T, Niwa M, Takaya Y. Rosmarinic acid in Argusia argentea inhibits |
| 1305 | snake venom-induced hemorrhage. J Nat Med. 2010;64:482–6. |
| 1306 | 56. Mendes MM, Vieira S, Gomes MSR, Paula VF, Alcântara TM, Homsi-Brandeburgo MI, |
| 1307 | et al. Triacontyl p-coumarate: An inhibitor of snake venom metalloproteinases. |
| 1308 | Phytochemistry. 2013;86:72–82. |
| 1309 | 57. Shukla PK, Gautam L, Sinha M, Kaur P, Sharma S, Singh TP. Structures and binding |
| 1310 | studies of the complexes of phospholipase A2 with five inhibitors. Biochim Biophys Acta |
| 1311 | (BBA)-Proteins Proteomics. 2015;1854(4):269–77. |
| 1312 | 58. Pereira NA, Pereira BMR, Nascimento MC, Parente JP, Mors WB. Pharmacological |
| 1313 | screening of plants recommended by folk medicine as snake venom antidotes; IV. |
| 1314 | Protection against Jararaca venom by isolated constituents1. Planta Med. 1994;60(02):99– |
| 1315 | 100. |
| 1316 | 59. Zalewski CA, Passero LFD, Melo ASRB, Corbett CEP, Laurenti MD, Toyama MH, et al. |
| 1317 | Evaluation of anti-inflammatory activity of derivatives from aerial parts of Baccharis |
| 1318 | uncinella. Pharm Biol. 2011;49(6):602–7. |
| 1319 | 60. Kuppusamy UR, Das NP. Protective effects of tannic acid and related natural compounds |
| 1320 | on Crotalus adamenteus subcutaneous poisoning in mice. Pharmacol Toxicol. 1993;72(4– |
| 1321 | 5):290–5. |
| 1322 | 61. Da Silva SL, Calgarotto AK, Chaar JS, Marangoni S. Isolation and characterization of |
| 1323 | ellagic acid derivatives isolated from Casearia sylvestris SW aqueous extract with anti- |
| 1324 | PLA2 activity. Toxicon. 2008;52(6):655–66. |

| 1325 | 62. Rodrigues CFB, Ferreira MJP, Belchor MN, Costa CRC, Novaes DP, dos Santos Junior |
| --- | --- |
| 1326 | AB, et al. Evaluation of the inhibitory potential of casuarictin, an ellagitannin isolated |
| 1327 | from white mangrove (Laguncularia racemosa) leaves, on snake venom secretory |
| 1328 | phospholipase A2. Mar Drugs. 2019;17(7):403. |
| 1329 | 63. Leanpolchareanchai J, Pithayanukul P, Bavovada R, Saparpakorn P. Molecular docking |
| 1330 | studies and anti-enzymatic activities of Thai mango seed kernel extract against snake |
| 1331 | venoms. Molecules. 2009;14(4):1404–22. |
| 1332 | 64. Lindahl M, Tagesson C. Flavonoids as phospholipase A 2 inhibitors: importance of their |
| 1333 | structure for selective inhibition of group II phospholipase A 2. Inflammation. |
| 1334 | 1997;21:347–56. |
| 1335 | 65. Min HM, Aye M, Taniguchi T, Miura N, Monde K, Ohzawa K, et al. A structure and an |
| 1336 | absolute configuration of (+)-alternamin, a new coumarin from Murraya alternans having |
| 1337 | antidote activity against snake venom. Tetrahedron Lett. 2007;48(35):6155–8. |
| 1338 | 66. Vander dos Santos R, Villalta-Romero F, Stanisic D, Borro L, Neshich G, Tasic L. Citrus |
| 1339 | bioflavonoid, hesperetin, as inhibitor of two thrombin-like snake venom serine proteases |
| 1340 | isolated from Crotalus simus. Toxicon. 2018;143:36–43. |
| 1341 | 67. Vander dos Santos R, Grillo G, Fonseca H, Stanisic D, Tasic L. Hesperetin as an inhibitor |
| 1342 | of the snake venom serine protease from Bothrops jararaca. Toxicon. 2021;198:64–72. |
| 1343 | 68. Gómez-Betancur I, Pereañez JA, Patiño AC, Benjumea D. Inhibitory effect of pinostrobin |
| 1344 | from Renealmia alpinia, on the enzymatic and biological activities of a PLA2. Int J Biol |
| 1345 | Macromol. 2016;89:35–42. |
| 1346 | 69. Kuppusamy UR, Das NP. Inhibitory effects of flavonoids on several venom |
| 1347 | hyaluronidases. Experientia. 1991;47:1196–200. |
| 1348 | 70. Nascimento LS, Nogueira-Souza PD, Rocha-Junior JRS, Monteiro-Machado M, Strauch |
| 1349 | MA, Prado SAL, et al. Phytochemical composition, antisnake venom and antibacterial |
| 1350 | activities of ethanolic extract of Aegiphila integrifolia (Jacq) Moldenke leaves. Toxicon. |

| 1351 | 2021;198:121–31. |
| --- | --- |
| 1352 | 71. Coriolano de Oliveira E, Alves Soares Cruz R, de Mello Amorim N, Guerra Santos M, |
| 1353 | Carlos Simas Pereira Junior L, Flores Sanchez EO, et al. Protective effect of the plant |
| 1354 | extracts of erythroxylum sp. against toxic effects induced by the venom of lachesis muta |
| 1355 | snake. Molecules. 2016;21(10):1350. |
| 1356 | 72. Lindahl M, Tagesson C. Selective inhibition of group II phospholipase A 2 by quercetin. |
| 1357 | Inflammation. 1993;17:573–82. |
| 1358 | 73. Cotrim CA, de Oliveira SCB, Diz Filho EBS, Fonseca FV, Baldissera Jr L, Antunes E, et |
| 1359 | al. Quercetin as an inhibitor of snake venom secretory phospholipase A2. Chem Biol |
| 1360 | Interact. 2011;189(1–2):9–16. |
| 1361 | 74. de Oliveira Toyama D, dos Santos Diz Filho EB, Cavada BS, da Rocha BAM, de Oliveira |
| 1362 | SCB, Cotrim CA, et al. Umbelliferone induces changes in the structure and |
| 1363 | pharmacological activities of Bn IV, a phospholipase A2 isoform isolated from Bothrops |
| 1364 | neuwiedi. Toxicon. 2011;57(6):851–60. |
| 1365 | 75. Girish KS, Kemparaju K. Inhibition of Naja naja venom hyaluronidase by plant-derived |
| 1366 | bioactive components and polysaccharides. Biochem. 2005;70:948–52. |
| 1367 | 76. Preciado LM, Rey-Suárez P, Henao IC, Pereañez JA. Betulinic, oleanolic and ursolic |
| 1368 | acids inhibit the enzymatic and biological effects induced by a PI snake venom |
| 1369 | metalloproteinase. Chem Biol Interact. 2018;279:219–26. |
| 1370 | 77. Gómez-Betancur I, Gogineni V, Salazar-Ospina A, León F. Perspective on the |
| 1371 | therapeutics of anti-snake venom. Molecules. 2019;24(18):3276. |
| 1372 | 78. Gopi K, Anbarasu K, Renu K, Jayanthi S, Vishwanath BS, Jayaraman G. Quercetin-3-O- |
| 1373 | rhamnoside from Euphorbia hirta protects against snake Venom induced toxicity. Biochim |
| 1374 | Biophys Acta (BBA)-General Subj. 2016;1860(7):1528–40. |
| 1375 | 79. Lu Y-N, Chai X-Y, Xu Z-R, Bi D, Ren H-Y, Zhao M, et al. Three new phenolic |

| 1376 | glycosides and a new triterpenoid from the stems of Scolopia chinensis. Planta Med. |
| --- | --- |
| 1377 | 2009;358–61. |
| 1378 | 80. Vale HF, M Mendes M, S Fernandes R, R Costa T, IS Hage-Melim L, A Sousa M, et al. |
| 1379 | Protective effect of Schizolobium parahyba flavonoids against snake venoms and isolated |
| 1380 | toxins. Curr Top Med Chem. 2011;11(20):2566–77. |
| 1381 | 81. Devi A, Namsa ND, Doley R. In silico and in vitro neutralization of PLA2 activity of |
| 1382 | Daboxin P by butein, mimosine and bakuchiol. Int J Biol Macromol. 2020;165:1066–78. |
| 1383 | 82. Omale S, Auta A, Amagon KI, Ighagbon M V. Anti-snake venom activity of flavonoids |
| 1384 | from the root bark extract of Parinari curatellifolia in Mice. IJPR. 1955; |
| 1385 | 83. Omara T, Kagoya S, Openy A, Omute T, Ssebulime S, Kiplagat KM, et al. Antivenin |
| 1386 | plants used for treatment of snakebites in Uganda: ethnobotanical reports and |
| 1387 | pharmacological evidences. Trop Med Health. 2020;48:1–16. |
| 1388 | 84. Gbolade AA. Nigerian medicinal plants with anti-snake venom activity—A review. J |
| 1389 | Malar Res Phytomedicine. 2021;1(1). |
| 1390 | 85. Soares AM, Ticli FK, Marcussi S, Lourenco M V, Januario AH, Sampaio S V, et al. |
| 1391 | Medicinal plants with inhibitory properties against snake venoms. Curr Med Chem. |
| 1392 | 2005;12(22):2625–41. |
| 1393 | 86. Nishijima CM, Rodrigues CM, Silva MA, Lopes-Ferreira M, Vilegas W, Hiruma-Lima |
| 1394 | CA. Anti-hemorrhagic activity of four Brazilian vegetable species against Bothrops |
| 1395 | jararaca venom. Molecules. 2009;14(3):1072–80. |
| 1396 | 87. Yusuf AJ, Aleku GA, Bello UR, Liman DU. Prospects and Challenges of Developing |
| 1397 | Plant‐Derived Snake Antivenin Natural Products: A Focus on West Africa. |
| 1398 | ChemMedChem. 2021;16(24):3635–48. |
| 1399 | 88. Ximenes RM, Alves RS, Pereira TP, Araújo RM, Silveira ER, Rabello MM, et al. |
| 1400 | Harpalycin 2 inhibits the enzymatic and platelet aggregation activities of PrTX-III, a D49 |

| 1401 | phospholipase A 2 from Bothrops pirajai venom. BMC Complement Altern Med. |
| --- | --- |
| 1402 | 2012;12:1–10. |
| 1403 | 89. Ximenes RM, Rabello MM, Araujo RM, Silveira ER, Fagundes FHR, Diz-Filho EBS, et |
| 1404 | al. Inhibition of neurotoxic secretory phospholipases A2 enzymatic, edematogenic, and |
| 1405 | myotoxic activities by harpalycin 2, an isoflavone isolated from Harpalyce brasiliana |
| 1406 | benth. Evidence‐Based Complement Altern Med. 2012;2012(1):987517. |
| 1407 | 90. Ferraz MC, Yoshida EH, Tavares RVS, Cogo JC, Cintra ACO, Dal Belo CA, et al. An |
| 1408 | isoflavone from Dipteryx alata Vogel is active against the in vitro neuromuscular paralysis |
| 1409 | of Bothrops jararacussu snake venom and bothropstoxin I, and prevents venom-induced |
| 1410 | myonecrosis. Molecules. 2014;19(5):5790–805. |
| 1411 | 91. Reyes-Chilpa R, Gómez-Garibay F, Quijano L, Magos-Guerrero GA, Ríos T. Preliminary |
| 1412 | results on the protective effect of (-)-edunol, a pterocarpan from Brongniartia |
| 1413 | podalyrioides (Leguminosae), against Bothrops atrox venom in mice. J Ethnopharmacol. |
| 1414 | 1994;42(3):199–203. |
| 1415 | 92. da Silva AJM, Coelho AL, Simas ABC, Moraes RAM, Pinheiro DA, Fernandes FFA, et |
| 1416 | al. Synthesis and pharmacological evaluation of prenylated and benzylated pterocarpans |
| 1417 | against snake venom. Bioorg Med Chem Lett. 2004;14(2):431–5. |
| 1418 | 93. Nakagawa M, Nakanishi K, Darko LL, Vick JA. Structures of cabenegrins AI and A-II, |
| 1419 | potent anti-snake venoms. Tetrahedron Lett. 1982;23(38):3855–8. |
| 1420 | 94. Mors WB, Do Nascimento MC, Parente J, Da Silva MH, Melo PA, Suarez-Kurtz G. |
| 1421 | Neutralization of lethal and myotoxic activities of South American rattlesnake venom by |
| 1422 | extracts and constituents of the plant Eclipta prostrata (Asteraceae). Toxicon. |
| 1423 | 1989;27(9):1003–9. |
| 1424 | 95. Melo PA, Do Nascimento MC, Mors WB, Suarez-Kurtz G. Inhibition of the myotoxic and |
| 1425 | hemorrhagic activities of crotalid venoms by Eclipta prostrata (Asteraceae) extracts and |
| 1426 | constituents. Toxicon. 1994;32(5):595–603. |

| 1427 | 96. Diogo LC, Fernandes RS, Marcussi S, Menaldo DL, Roberto PG, Matrangulo PVF, et al. |
| --- | --- |
| 1428 | Inhibition of snake venoms and phospholipases A2 by extracts from native and genetically |
| 1429 | modified Eclipta alba: isolation of active coumestans. Basic Clin Pharmacol Toxicol. |
| 1430 | 2009;104(4):293–9. |
| 1431 | 97. Cedro RCA, Menaldo DL, Costa TR, Zoccal KF, Sartim MA, Santos-Filho NA, et al. |
| 1432 | Cytotoxic and inflammatory potential of a phospholipase A 2 from Bothrops jararaca |
| 1433 | snake venom. J Venom Anim Toxins Incl Trop Dis. 2018;24:33. |
| 1434 | 98. da Silva Jr NJ, Aird SD. Prey specificity, comparative lethality and compositional |
| 1435 | differences of coral snake venoms. Comp Biochem Physiol Part C Toxicol Pharmacol. |
| 1436 | 2001;128(3):425–56. |
| 1437 | 99. Chai X-Y, Ren H-Y, Xu Z-R, Bai C-C, Zhou F-R, Ling S-K, et al. Investigation of two |
| 1438 | Flacourtiaceae plants: Bennettiodendron leprosipes and Flacourtia ramontchi. Planta Med. |
| 1439 | 2009;75(11):1246–52. |
| 1440 | 100. Lu Y-N, Chai X-Y, Xu Z-R, Bi D, Ren H-Y, Zhao M, et al. Three new phenolic |
| 1441 | glycosides and a new triterpenoid from the stems of Scolopia chinensis. Planta Med. |
| 1442 | 2010;76(04):358–61. |
| 1443 | 101. Ahmad VU, Abbasi MA, Hussain H, Akhtar MN, Farooq U, Fatima N, et al. Phenolic |
| 1444 | glycosides from Symplocos racemosa: natural inhibitors of phosphodiesterase I. |
| 1445 | Phytochemistry. 2003;63(2):217–20. |
| 1446 | 102. Strauch MA, Tomaz MA, Monteiro-Machado M, Cons BL, Patrao-Neto FC, Teixeira- |
| 1447 | Cruz J da M, et al. Lapachol and synthetic derivatives: in vitro and in vivo activities |
| 1448 | against Bothrops snake venoms. PLoS One. 2019;14(1):e0211229. |
| 1449 | 103. Vieira SAPB, Dos Santos BM, Júnior CDS, de Paula VF, Gomes MSR, Ferreira GM, et |
| 1450 | al. Isohemigossypolone: Antiophidic properties of a naphthoquinone isolated from Pachira |
| 1451 | aquatica Aubl. Comp Biochem Physiol Part C Toxicol Pharmacol. 2021;245:109028. |
| 1452 | 104. Selvanayagam ZE, Gnanavendhan SG, Balakrishna K, Rao RB, Sivaraman J, |

| 1453 | Subramanian K, et al. Ehretianone, a novel quinonoid xanthene from Ehretia buxifolia |
| --- | --- |
| 1454 | with antisnake venom activity. J Nat Prod. 1996;59(7):664–7. |
| 1455 | 105. Hung Y-C, Sava V, Hong M-Y, Huang GS. Inhibitory effects on phospholipase A2 and |
| 1456 | antivenin activity of melanin extracted from Thea sinensis Linn. Life Sci. |
| 1457 | 2004;74(16):2037–47. |
| 1458 | 106. Salama R, Sattayasai J, Gande AK, Sattayasai N, Davis M, Lattmann E. Identification and |
| 1459 | evaluation of agents isolated from traditionally used herbs against Ophiophagus hannah |
| 1460 | venom. Drug Discov Ther. 2012;6(1):18–23. |
| 1461 | 107. Magalhães SFV, Peixoto HM, de Almeida Gonçalves Sachett J, Oliveira SS, Alves EC, |
| 1462 | dos Santos Ibiapina HN, et al. Snakebite envenomation in the Brazilian Amazon: a cost- |
| 1463 | of-illness study. Trans R Soc Trop Med Hyg. 2020;114(9):642–9. |
| 1464 | 108. Ferreira LAF, Henriques OB, Andreoni AAS, Vital GRF, Campos MMC, Habermehl GG, |
| 1465 | et al. Antivenom and biological effects of ar-turmerone isolated from Curcuma longa |
| 1466 | (Zingiberaceae). Toxicon. 1992;30(10):1211–8. |
| 1467 | 109. Lattmann E, Sattayasai J, Sattayasai N, Staaf A, Phimmasone S, Schwalbe CH, et al. In- |
| 1468 | vitro and in-vivo antivenin activity of 2-[2-(5, 5, 8a-trimethyl-2-methylene-decahydro- |
| 1469 | naphthalen-1-yl)-ethylidene]-succinaldehyde against Ophiophagus hannah venom. J |
| 1470 | Pharm Pharmacol. 2010;62(2):257–62. |
| 1471 | 110. Januário AH, Santos SL, Marcussi S, Mazzi M V, Pietro RCLR, Sato DN, et al. Neo- |
| 1472 | clerodane diterpenoid, a new metalloprotease snake venom inhibitor from Baccharis |
| 1473 | trimera (Asteraceae): anti-proteolytic and anti-hemorrhagic properties. Chem Biol |
| 1474 | Interact. 2004;150(3):243–51. |
| 1475 | 111. Fatima N, Tapondjou LA, Lontsi D, Sondengam BL, Atta-Ur-Rahman, Choudhary MI. |
| 1476 | Quinovic acid glycosides from Mitragyna stipulosa-first examples of natural inhibitors of |
| 1477 | snake venom phosphodiesterase I. Nat Prod Lett. 2002;16(6):389–93. |
| 1478 | 112. Fernandes FFA, Tomaz MA, El-Kik CZ, Monteiro-Machado M, Strauch MA, Cons BL, et |

| 1479 | al. Counteraction of Bothrops snake venoms by Combretum leprosum root extract and |
| --- | --- |
| 1480 | arjunolic acid. J Ethnopharmacol. 2014;155(1):552–62. |
| 1481 | 113. Chatterjee I, Chakravarty AK, Gomes A. Daboia russellii and Naja kaouthia venom |
| 1482 | neutralization by lupeol acetate isolated from the root extract of Indian sarsaparilla |
| 1483 | Hemidesmus indicus R. Br. J Ethnopharmacol. 2006;106(1):38–43. |
| 1484 | 114. do Rosário Daros M, de Abreu Matos FJ, Parente JP. A new triterpenoid saponin, |
| 1485 | Bredemeyeroside B, from the roots of Bredemeyera floribunda. Planta Med. |
| 1486 | 1996;62(06):523–7. |
| 1487 | 115. Pereira BMR, Daros M do R, Parente JP, Matos FJ de A. Bredemeyeroside D, a novel |
| 1488 | triterpenoid saponin from Bredemeyera floribunda: a potent snake venom antidote activity |
| 1489 | on mice. Phyther Res. 1996;10(8):666–9. |
| 1490 | 116. da Silva JO, Fernandes RS, Ticli FK, Oliveira CZ, Mazzi M V, Franco JJ, et al. |
| 1491 | Triterpenoid saponins, new metalloprotease snake venom inhibitors isolated from |
| 1492 | Pentaclethra macroloba. Toxicon. 2007;50(2):283–91. |
| 1493 | 117. Mostafa M, Nahar N, Mosihuzzaman M, Sokeng SD, Fatima N, Atta-ur-Rahman, et al. |
| 1494 | Phosphodiesterase-I inhibitor quinovic acid glycosides from Bridelia ndellensis. Nat Prod |
| 1495 | Res. 2006;20(7):686–92. |
| 1496 | 118. MS V, More VS, Zameer F, Muddapur U, More SS. Ethnomedicinal plants and isolated |
| 1497 | compounds against Snake venom activity: A review. 2021; |
| 1498 | 119. Fozing CDA, Ali Z, Ngadjui BT, Choudhary MI, Kapche GDWF, Abegaz BM, et al. |
| 1499 | Phosphodiesterase I-inhibiting Diels-Alder adducts from the leaves of Morus mesozygia. |
| 1500 | Planta Med. 2012;78(02):154–9. |
| 1501 | 120. Mahadeswaraswamy YH, Manjula B, Devaraja S, Girish KS, Kemparaju K. Daboia |
| 1502 | russelli venom hyaluronidase: purification, characterization and inhibition by β-3-(3- |
| 1503 | hydroxy-4-oxopyridyl) α-amino-propionic Acid. Curr Top Med Chem. 2011;11(20):2556– |
| 1504 | 65. |

| 1505 | 121. Sarkhel S, Chakravarty AK, Das R, Gomes A, Gomes A. Snake venom neutralising factor |
| --- | --- |
| 1506 | from the root extract of Emblica officinalis Linn. Orient Pharm Exp Med. 2011;11:25–33. |
| 1507 | 122. Núñez V, Castro V, Murillo R, Ponce-Soto LA, Merfort I, Lomonte B. Inhibitory effects |
| 1508 | of Piper umbellatum and Piper peltatum extracts towards myotoxic phospholipases A2 |
| 1509 | from Bothrops snake venoms: isolation of 4-nerolidylcatechol as active principle. |
| 1510 | Phytochemistry. 2005;66(9):1017–25. |
| 1511 | 123. Venkatesan C, Sarathi M, Balasubramanian G, Thomas J, Balachander V, Babu VS, et al. |
| 1512 | Antivenom activity of triterpenoid (C34H68O2) from Leucas aspera Linn. against Naja |
| 1513 | naja naja venom induced toxicity: Antioxidant and histological study in mice. Hum Exp |
| 1514 | Toxicol. 2014;33(4):336–59. |
| 1515 | 124. Raghavamma ST V, Rao NR, Rao GD. Inhibitory potential of important phytochemicals |
| 1516 | from Pergularia daemia (Forsk.) chiov., on snake venom (Naja naja). J Genet Eng |
| 1517 | Biotechnol. 2016;14(1):211–7. |
| 1518 | 125. Dharmappa KK, Mohamed R, Shivaprasad H V, Vishwanath BS. Genistein, a potent |
| 1519 | inhibitor of secretory phospholipase A 2: a new insight in down regulation of |
| 1520 | inflammation. Inflammopharmacology. 2010;18:25–31. |
| 1521 | 126. H Mahadeswaraswamy Y, Manjula B, Devaraja S, S Girish K, Kemparaju K. Daboia |
| 1522 | russelli venom hyaluronidase: purification, characterization and inhibition by β-3-(3- |
| 1523 | hydroxy-4-oxopyridyl) α-amino-propionic Acid. Curr Top Med Chem. 2011;11(20):2556– |
| 1524 | 65. |
| 1525 | 127. Sakthivel G, Dey A, Nongalleima K, Chavali M, Rimal Isaac RS, Singh NS, et al. In vitro |
| 1526 | and in vivo evaluation of polyherbal formulation against Russell’s viper and cobra venom |
| 1527 | and screening of bioactive components by docking studies. Evidence‐Based Complement |
| 1528 | Altern Med. 2013;2013(1):781216. |
| 1529 | 128. Opiyo SA, Njoroge PW. Plant extracts and terpenes with antivenom properties. 2024; |
| 1530 | 129. Halilu EM, October N, Ugwah-Oguejiofor CJ, Jega AY, Nefai MS. Anti-snake venom and |

| 1531 | analgesic activities of extracts and betulinic and oleanolic acids isolated from Parinari |
| --- | --- |
| 1532 | curatellifolia. J Med plants Econ Dev. 2020;4(1):1–8. |
| 1533 | 130. Samy RP, Gopalakrishnakone P, Chow VTK. Therapeutic application of natural inhibitors |
| 1534 | against snake venom phospholipase A2. Bioinformation. 2012;8(1):48. |
| 1535 | 131. Adamu M, Uttu AJ, Ajala A, Obansa RM, Madumelu M. A short review on plants used as |
| 1536 | anti-snake venom. J Chem Rev. 2023;5(3):341–52. |
| 1537 | 132. Singh P, Yasir M, Hazarika R, Sugunan S, Shrivastava R. A review on venom enzymes |
| 1538 | neutralizing ability of secondary metabolites from medicinal plants. J pharmacopuncture. |
| 1539 | 2017;20(3):173. |
| 1540 | 133. de Sousa Ferreira S, da Silva DP, Torres-Rêgo M, da Silva-Júnior AA, de Freitas |
| 1541 | Fernandes-Pedrosa M. The potential of phenolic acids in therapy against snakebites: A |
| 1542 | review. Toxicon. 2022;208:1–12. |
| 1543 | 134. Xiao H, Pan H, Liao K, Yang M, Huang C. Snake Venom PLA2, a Promising Target for |
| 1544 | Broad‐Spectrum Antivenom Drug Development. Biomed Res Int. 2017;2017(1):6592820. |
| 1545 | 135. Dauda MM, Dauda G, Abdullahi SM, Sada H, Sule MI, Hassan HS, et al. Isolation and |
| 1546 | characterization of a novel dihydrostilbene from Indigofera capitata kotschy with |
| 1547 | antivenin activity against Naja nigricollis venom. Discov Chem. 2025;2(1):43. |
| 1548 | 136. Maria TAN, CARRANZA M, MALABED R, REYES Y, FRANCO F, LINIS V, et al. |
| 1549 | Antioxidant, cytotoxic, and anti-venom activity of Alstonia parvifolia Merr. Bark. Asian |
| 1550 | Pac J Trop Biomed. 2021;460–8. |
| 1551 | 137. Kibet S, Kimani NM, Mwanza SS, Mudalungu CM, Santos CBR, Tanga CM. Unveiling |
| 1552 | the Potential of Ent -Kaurane Diterpenoids : Multifaceted Natural Products for Drug |
| 1553 | Discovery. 2024;1–20. |
| 1554 | 138. Wekesa EN, Kimani NM, Kituyi SN, Omosa LK, Santos CBR. Therapeutic potential of |
| 1555 | the genus Zanthoxylum phytochemicals: A theoretical ADME/Tox analysis. South |

| 1556 | African J Bot. 2023;162:129–41. |
| --- | --- |
| 1557 | 139. Falade VA, Adelusi TI, Adedotun IO, Abdul-Hammed M, Lawal TA, Agboluaje SA. In |
| 1558 | silico investigation of saponins and tannins as potential inhibitors of SARS-CoV-2 main |
| 1559 | protease (M pro). Silico Pharmacol. 2021;9:1–15. |
| 1560 | 140. Żołek T, Maciejewska D. Theoretical evaluation of ADMET properties for coumarin |
| 1561 | derivatives as compounds with therapeutic potential. Eur J Pharm Sci. 2017;109:486–502. |
| 1562 | 141. Durán-Iturbide NA, Díaz-Eufracio BI, Medina-Franco JL. In Silico ADME/Tox Profiling |
| 1563 | of Natural Products: A Focus on BIOFACQUIM. ACS Omega. 2020;5(26):16076–84. |
| 1564 | 142. Fatima S, Gupta P, Sharma S, Sharma A, Agarwal SM. ADMET profiling of |
| 1565 | geographically diverse phytochemical using chemoinformatic tools. Future Med Chem. |
| 1566 | 2020;12(1):69–87. |
| 1567 | 143. Bildziukevich U, Wimmerová M, Wimmer Z. Saponins of selected triterpenoids as |
| 1568 | potential therapeutic agents: A review. Pharmaceuticals. 2023;16(3):386. |
| 1569 | 144. Mortuza MG, Roni MAH, Kumer A, Biswas S, Saleh MA, Islam S, et al. A |
| 1570 | Computational Study on Selected Alkaloids as SARS‐CoV‐2 Inhibitors: PASS Prediction, |
| 1571 | Molecular Docking, ADMET Analysis, DFT, and Molecular Dynamics Simulations. |
| 1572 | Biochem Res Int. 2023;2023(1):9975275. |
| 1573 | 145. Han S, Mei L, Quach T, Porter C, Trevaskis N. Lipophilic conjugates of drugs: a tool to |
| 1574 | improve drug pharmacokinetic and therapeutic profiles. Pharm Res. 2021;38(9):1497– |
| 1575 | 518. |
| 1576 | 146. Wanat K. Biological barriers, and the influence of protein binding on the passage of drugs |
| 1577 | across them. Mol Biol Rep. 2020;47(4):3221–31. |
| 1578 | 147. Zuo H-L, Huang H-Y, Lin Y-C-D, Cai X-X, Kong X-J, Luo D-L, et al. Enzyme activity of |
| 1579 | natural products on cytochrome P450. Molecules. 2022;27(2):515. |

1580

1581

148. Smith DA, Beaumont K, Maurer TS, Di L. Relevance of half-life in drug design: Miniperspective. J Med Chem. 2017;61(10):4273–82.

1582

1583

1584

1585

149. Oliveira CA, Mercês ÉAB, Portela FS, Malheiro LFL, Silva HBL, De Benedictis LM, et al. An integrated view of cisplatin-induced nephrotoxicity, hepatotoxicity, and cardiotoxicity: characteristics, common molecular mechanisms, and current clinical management. Clin Exp Nephrol. 2024;28(8):711–27.

1586

1587

150. Dobrek L. A synopsis of current theories on drug-induced nephrotoxicity. Life. 2023;13(2):325.

1588

1589

1590

1591

151. Zhang D, Wei C, Hop CECA, Wright MR, Hu M, Lai Y, et al. Intestinal excretion, intestinal recirculation, and renal tubule reabsorption are underappreciated mechanisms that drive the distribution and pharmacokinetic behavior of small molecule drugs. J Med Chem. 2021;64(11):7045–59.

1592

1593

152. Adibah KZM, Azzreena MA. Plant toxins: alkaloids and their toxicities. GSC Biol Pharm Sci. 2019;6(2):21–9.

1594

1595

1596

1597

1598

153. Rajput A, Sharma R, Bharti R. Pharmacological activities and toxicities of alkaloids on human health. Mater Today Proc. 2022;48:1407–15.

Supporting figure

# [Click here to access/download](https://www2.cloud.editorialmanager.com/pntd/download.aspx?id=1433288&guid=f8223fd8-b91f-4c3b-a999-e6ec0915cf1f&scheme=1)

[**Supporting Information** Supplimentary File Figure S1.docx](https://www2.cloud.editorialmanager.com/pntd/download.aspx?id=1433288&guid=f8223fd8-b91f-4c3b-a999-e6ec0915cf1f&scheme=1)

Supporting Information

# [Click here to access/download](https://www2.cloud.editorialmanager.com/pntd/download.aspx?id=1433289&guid=7c067b2c-f244-461f-a8bc-0d78f4786d9c&scheme=1)

[**Supporting Information** Supplimentary File Table S1.docx](https://www2.cloud.editorialmanager.com/pntd/download.aspx?id=1433289&guid=7c067b2c-f244-461f-a8bc-0d78f4786d9c&scheme=1)
