# Supplementary material for: A review and in silico screening of plant-derived snake venom/toxin inhibitors: ADMET, drug-likeness, and medicinal chemistry profiling
Source: PLoS Negl Trop Dis. 2025 Oct 9;19(10):e0013579. doi: 10.1371/journal.pntd.0013579 (PMC12527186; doi:10.1371/journal.pntd.0013579)
Supplement: Attachment — Submitted filename: Response to Reviewers (4).docx [file pntd.0013579.s008.docx]

Dear Editor,

We value the time you and the reviewers spent reviewing our article and offering insightful comments. Your insightful and helpful remarks inspired potential improvements to the present version. The authors have given great thought to the comments and did our best to respond to each one. After thorough revisions, the work will live up to your high standards. The point-by-point responses are given below. The yellow highlights in the manuscript indicate all the changes.

**Response to reviewer #1**

**Methods**

**[Comment ]: This manuscript presents a comprehensive in silico evaluation of plant-derived compounds for potential antivenom application. The authors conducted an extensive literature review to curate a library of over 200 phytochemicals, which were subsequently screened using ADMET (Absorption, Distribution, Metabolism, Excretion, and Toxicity) profiling tools. The study is well-structured and addresses an important area in drug discovery, particularly for underserved conditions like snake envenomation. The strength of the manuscript lies in its methodical approach to compound selection and its rigorous computational assessment. The use of drug-likeness filters and ADMET parameters provides a rational basis for identifying lead candidates. Notably, only three compounds met the criteria for favorable pharmacokinetic and safety profiles, emphasizing both the selectivity and the utility of in silico tools in early-stage drug development.**

**Response:** We sincerely thank you for the positive and encouraging feedback.

**Results**

**[Comment]** **The authors evaluated over 200 plant compounds using established ADMET profiling tools. The computational analysis filtered out the majority of compounds due to poor pharmacokinetic or toxicity parameters. Ultimately, only three compounds met the threshold for drug-likeness, with acceptable properties for absorption, metabolism, and toxicity. These compounds showed favorable bioavailability and pharmacological potential based on the in silico models employed. The table needs improvement so that it appears professional like.**

**Response:** Thank you for your constructive feedback. We have addressed your comment by improving the table's format to ensure a more professional appearance. Please see the yellow highlights in the revised manuscript for the specific changes made.

**Conclusions**

**[comment] The study demonstrates that rigorous computational screening can effectively narrow down large libraries of natural products to a small number of promising drug-like candidates. The identification of three phytochemicals with favorable ADMET profiles supports the feasibility of plant-derived inhibitors as leads for antivenom therapy. The authors rightly suggest that these compounds should undergo further in vitro and in vivo validation to confirm their therapeutic potential.**

**Response:** Thank you for your valuable feedback.

**Editorial and Data Presentation Modifications**

**[Comment] Include a short discussion on the known or predicted biological activities of the three shortlisted compounds, especially in the context of venom toxin classes (e.g., metalloproteinases, PLA₂, neurotoxins). A brief description of the authors’ future plans for in vitro or in vivo validation would improve the translational value of the study. Highlight the structures of the 3 compounds in the main manuscript.**

**Response:** Thank you for the insightful suggestions. We have now included a short discussion on the known or predicted biological activities of the three prioritized compounds. Additionally, we have outlined our future plans for in vitro and in vivo validation to enhance the translational value of the study. The chemical structures of the three compounds have also been highlighted in the main manuscript as requested. Please refer to the revised sections marked in yellow.

**Summary and General Comments**

**[comment] This manuscript presents a rigorous and relevant computational strategy to identify drug-like phytochemicals with antivenom potential. With minor revisions, the work will be a strong contribution to the fields of toxicology and natural product drug discovery.**

**Response:** Thank you for the encouraging feedback. We appreciate your recognition of the relevance and rigor of our computational approach. We have addressed the suggested minor revisions to enhance the clarity and impact of the manuscript.

**Response to reviewer #2**

**Methods**

**[Comment]: Properly done.**

**Response:** Thank you for the positive feedback.

**Results**

**[Comment]** **Appropriately presented.**

**Response:** Thank you for your positive comment.

**Conclusions**

**[comment] Needs minor modifications.**

**Response:** Thank you for the feedback. We have addressed the minor modifications as requested.

**Editorial and Data Presentation Modifications**

**[Comment] Minor revision**

**Response:** Thank you. We have implemented the minor revisions accordingly. Please refer to the revised sections marked in yellow.

**Summary and General Comments**

**[comment] Prince Ojuka et al., have systematically executed the job of compiling the plant compounds exhibiting snake venom/toxins inhibiting/neutralizing property. It is an elaborate study highlighting the drug-likeness of compounds such as coumarins, benzenoids, polyketides, and isoflavanoids for treating venomous snake bite. The manuscript is well written, however may be accepted with minor modifications.**

**Response:** Thank you for the positive feedback. We appreciate your remarks and have made the suggested minor modifications in the revised manuscript.

**[comment] f= mention the author`s affiliation.**

**Short title: It is not appropriate, require modification suitably.**

**Response:** Thank you for the valuable feedback. This has been updated

**Summary and General Comments**

**[comment] The analysis revealed 11distinct chemical classes (Figure 2), with Terpenoids (37), Flavonoids (32), Benzenoids (9), Alkaloids (17), Isoflavonoids (10), Hydroxycinnamic Acids (8), Saponins (10), Modified Glycosides (8), Polyketides (4), Tannins (5), and coumarins (3). Additionally, other identified compounds were 70.**

**Comment: In addition to Figure 2, assigning the plant compounds presented in Figure S1 under the above mentioned respective categories appear more meaningful and convincing.**

**Response:** We appreciate your insightful suggestion. As advised, we have now assigned the plant compounds presented in Figure S1 to their respective chemical classes, corresponding to those identified in Figure 2. Please see the revised supplementary material figure S1.

**[comment] (Lines 1127 and 1128) The results underscore the 1128 relevance of natural products as promising sources for antivenom agents and provide valuable leads for the development of new antivenom treatments.**

**Line 1133, antivenom agents**

**Comment: Though the word antivenom may be used, it is better to use a different word, may be therapeutic agents to avoid confusions.**

**Response:** Thank you for this observation. We have revised accordingly.

**[comment] The authors have to specify in their conclusion, whether to use compounds 23, 97, and 98 independently/together in what ratios, and further, compounds alone or as auxiliary agents along with antivenom to treat fatal snake bite.**

**Response:** We thank you for this insightful suggestion. At the current stage, our study is primarily computational and exploratory, aimed at identifying promising plant-derived compounds with potential antivenom activity. While compounds 23, 97, and 98 have demonstrated favorable medicinal properties, experimental validation (in vitro and in vivo) is still required to determine their individual or synergistic efficacy, safety, and appropriate dosing strategies. Therefore, we believe it would be premature to propose specific ratios or combinational use, or to recommend their application as standalone or adjunctive agents with conventional antivenom. However, we have updated the conclusion to acknowledge this critical next step and state that future studies will be directed toward such experimental validations and the results will enable us propose appropriate dosing ratios.

**Response to reviewer #3**

**Methods**

**[Comment]: Lack of regional diversity: Authors presented plethora of compounds from medicinal plants against snake venoms mainly from Asia and South America continents. However, there are several researches from other continents on phytocompounds against snake venom toxins that meet the criteria set by the authors. It will make a more balanced and globally representative review if studies from other regions are also included to reflect regional diversity.**

**Response:** We sincerely thank you for highlighting the importance of regional diversity in our review. We acknowledge that a more globally representative inclusion of studies could enhance the comprehensiveness of our manuscript. However, as outlined in Section 2.2 (Search Strategy) and Sections 2.3–2.4 (Inclusion and Exclusion Criteria), our review was based on a rigorous and predefined selection process. We conducted an extensive literature search across major scientific databases (Google Scholar, ScienceDirect, Web of Science, Scopus, Springer, and PubMed), using a broad combination of keywords and Boolean operators relevant to snake venom neutralization by plant-derived compounds. Only peer-reviewed studies published in English up to December 2024 and those reporting bioactivity data of isolated plant compounds tested in in vitro and/or in vivo models were considered. This approach, while methodologically robust, may have inadvertently favored research from regions where such detailed compound-level studies are more prevalent particularly Asia and South America, where traditional medicinal plant use and snakebite incidence are high. It was not our intention to underrepresent other regions, but rather a reflection of the current availability of studies that met our strict inclusion criteria.

We acknowledge this as a limitation of our study. As discussed in the updated Section 4 (Methodological Limitations), our reliance on specific keywords and Boolean combinations guided by the PRISMA framework may have led to the exclusion of some relevant studies that did not meet these descriptors despite their merit. We recognize the importance of regional representation in building a globally inclusive understanding of snakebite therapeutics, and future iterations of this work will aim to integrate a more geographically balanced selection by refining search parameters and including a broader spectrum of regional literature. Nonetheless, we believe that the current review makes a significant contribution by consolidating plant-derived compounds with demonstrated antivenom activities and offering insights into their drug-likeness and ADMET properties. We are confident that this foundation will stimulate further investigation into phytotherapeutics from underrepresented regions.

**Results**

**[Comment]** **Mechanistic organization: Toxins and their inhibitors should be discussed based on molecular targets (e.g., PLA2, SVMPs, neurotoxins) to enhance clarity and mechanistic insight. Also, the underlying mechanisms, pharmacokinetics, and pharmacodynamics were not discussed and these are highlighted by authors in the justification as the major gaps in previous reviews.**

**Response:** Thank you for your feedback. The manuscript has been revised to organize the discussion based on molecular targets (e.g., PLA₂, SVMPs, neurotoxins) and to include the mechanisms of action. Additionally, we have incorporated relevant pharmacokinetic and pharmacodynamic in the discussion section. These changes are highlighted in yellow in the revised manuscript.

**Conclusions**

**[comment] In silico overinterpretation: The assertion that some compounds are ready for drug development is premature and should be rephrased. Authors should emphasize the predictive not confirmatory nature of the computational analysis.**

**Response:** Thank you for your valuable observation. We agree that in silico analyses are predictive and not confirmatory. The relevant statements have been rephrased to reflect the exploratory nature of the computational findings, avoiding any implication that the compounds are ready for drug development. These revisions are highlighted in yellow in the manuscript.

**Summary and General Comments**

**[comment] This study has the potential to make a significant contribution to venom research and natural antivenom discovery. However, substantial revisions are necessary to enhance the manuscript's scientific rigor, global balance, and structural clarity. I recommend a major revision.**

**Response:** We sincerely appreciate your thoughtful feedback and recognition of the potential contribution of our study to venom research and natural antivenom discovery. In response, we have undertaken substantial revisions to improve the manuscript’s scientific rigor, and enhance structural clarity. All major changes have been highlighted in yellow within the revised manuscript for your convenience. We hope the updated version meets your expectations.
