# Supplementary material for: A review and in silico screening of plant-derived snake venom/toxin inhibitors: ADMET, drug-likeness, and medicinal chemistry profiling
Source: PLoS Negl Trop Dis. 2025 Oct 9;19(10):e0013579. doi: 10.1371/journal.pntd.0013579 (PMC12527186; doi:10.1371/journal.pntd.0013579)
Supplement: Attachment — Submitted filename: Comment.docx [file pntd.0013579.s007.docx]

Notably, anisic acid
achieved 100% neutralization of lethality and defibrinogenation caused by N. kaouthia,
D. russelii, O. hannah, and E. carinatus venoms in both in vivo and in vitro studies.
Labdane lactone and labdane trialdehyde, isolated from C. antinaia and C.
zedoaroides, respectively, exhibited significant venom inhibition at a concentration of
10 µg/mL.

**Comment:** The above statement in the abstract indicates that all the plant components included in this study are in pure form. Thus, their molecular mass is known, therefore, it is better to present their molar concentrations instead of micrograms/ml. This may be extended to rest of the text wherever necessary.
